# A Timescale for Evolution, Population Expansion, and Spatial Spread of an Emerging Clone of Methicillin-Resistant *Staphylococcus aureus*

**DOI:** 10.1371/journal.ppat.1000855

**Published:** 2010-04-08

**Authors:** Ulrich Nübel, Janina Dordel, Kevin Kurt, Birgit Strommenger, Henrik Westh, Sanjay K. Shukla, Helena Žemličková, Raphaël Leblois, Thierry Wirth, Thibaut Jombart, François Balloux, Wolfgang Witte

**Affiliations:** 1 Robert Koch Institut, Wernigerode, Germany; 2 Hvidovre Hospital, Hvidovre, Denmark; University of Copenhagen, Faculty of Health, Copenhagen, Denmark; 3 Marshfield Clinic Research Foundation, Molecular Microbiology Laboratory, Marshfield, Wisconsin, United States of America; 4 National Institute of Public Health, Prague, Czech Republic; 5 Muséum National d'Histoire Naturelle - Ecole pratique des Hautes Etudes - Centre National de la Recherche Scientifique, Department of Systematics and Evolution, Paris, France; 6 Imperial College Faculty of Medicine, Department of Infectious Disease Epidemiology, Medical Research Council Centre for Outbreak Analysis and Modelling, London, United Kingdom; The University of Texas-Houston Medical School, United States of America

## Abstract

Due to the lack of fossil evidence, the timescales of bacterial evolution are largely unknown. The speed with which genetic change accumulates in populations of pathogenic bacteria, however, is a key parameter that is crucial for understanding the emergence of traits such as increased virulence or antibiotic resistance, together with the forces driving pathogen spread. Methicillin-resistant *Staphylococcus aureus* (MRSA) is a common cause of hospital-acquired infections. We have investigated an MRSA strain (ST225) that is highly prevalent in hospitals in Central Europe. By using mutation discovery at 269 genetic loci (118,804 basepairs) within an international isolate collection, we ascertained extremely low diversity among European ST225 isolates, indicating that a recent population bottleneck had preceded the expansion of this clone. In contrast, US isolates were more divergent, suggesting they represent the ancestral population. While diversity was low, however, our results demonstrate that the short-term evolutionary rate in this natural population of MRSA resulted in the accumulation of measurable DNA sequence variation within two decades, which we could exploit to reconstruct its recent demographic history and the spatiotemporal dynamics of spread. By applying Bayesian coalescent methods on DNA sequences serially sampled through time, we estimated that ST225 had diverged since approximately 1990 (1987 to 1994), and that expansion of the European clade began in 1995 (1991 to 1999), several years before the new clone was recognized. Demographic analysis based on DNA sequence variation indicated a sharp increase of bacterial population size from 2001 to 2004, which is concordant with the reported prevalence of this strain in several European countries. A detailed ancestry-based reconstruction of the spatiotemporal dispersal dynamics suggested a pattern of frequent transmission of the ST225 clone among hospitals within Central Europe. In addition, comparative genomics indicated complex bacteriophage dynamics.

## Author Summary

Because fossils of bacteria do not exist or are morphologically indeterminate, the timescales of bacterial evolution are widely unknown. We have investigated the short-term evolution of a particular strain of methicillin-resistant *Staphylococcus aureus* (MRSA), a notorious cause of hospital-associated infections. By comparing 118 kilobases of DNA from MRSA isolates that had been collected at different points in time, we demonstrate that this strain has accumulated measurable DNA sequence variation within two decades. Further, we exploited this sequence diversity to estimate the short-term evolutionary rate and to date divergence times without paleontological calibration, and to reconstruct the recent demographic expansion and spatial spread of this MRSA.

## Introduction

Clinical microbiologists have frequently been astonished by the impressive capability of pathogenic bacteria to acquire novel traits such as antimicrobial resistance. However, the actual speed at which nucleotide substitutions, entire genes, or complex mobile genetic elements are gained and lost in bacterial populations has rarely been determined [1],[2],[3],[4]. A measure of the real-time nucleotide substitution rate in natural populations of pathogenic bacteria would enable the dating of evolutionary events and the reconstruction of a pathogen's demographic history based on DNA sequence variation, which ultimately could provide fundamental insights into the forces driving pathogen emergence and spread [2],[5].

Methicillin-resistant *Staphylococcus aureus* (MRSA) are a common cause of hospital-acquired infections, imposing a heavy burden on patients and health care resources [6]. The prevention and treatment of such infections has become increasingly difficult due to this bacterium's ability to acquire resistance against all classes of antibiotics. *Staphylococcus aureus* has long been known to cause local outbreaks and regional epidemics of hospital infections, where the causative strains – identified through bacterial typing – may spread both within and across hospital wards, and among different hospitals [7]. Contemporary typing of *S. aureus* is performed by using molecular techniques, including DNA macrorestriction (pulsed field gel electrophoresis) and DNA sequence-based methods. Among the latter, multilocus sequence typing (MLST), which indexes variation at seven slowly evolving genetic loci, has been extremely useful to gain a basic understanding of the population structure of *S. aureus* [8]. While more than 1,400 MLST-based sequence types (ST) have been reported for *S. aureus* to date, most of this diversity is clustered in a limited number of clonal complexes [8]. The worldwide predominance of a few clonal lineages among MRSA has resulted in the conception that MRSA strains may spread globally very rapidly [9],[10]. However, by investigating the diversity and phylogeography of one such clone (ST5) in greater detail, we have recently detected considerable spatial subdivision among populations from different localities, indicating that the dispersal of this clone over long distances happens rarely in comparison to the frequency at which novel MRSA arise through acquisition of the genetic methicillin-resistance island SCC*mec* [11].

In the present study, we have investigated the evolutionary history of an MRSA strain that recently emerged in Central Europe. By MLST, this strain is identified as sequence type ST225 (allelic profile, 1-4-1-4-12-25-10), which is a single locus variant of ST5, the presumed ancestor of clonal complex CC5 [8]. While ST225 had been discovered first among isolates collected during the 1990s in the USA [8],[12], it was not detected in any European country before the year 2000 [13],[14],[15],[16],[17],[18]. Since 2001, however, its reported proportional abundance in Germany increased very rapidly [14], and it was also reported from hospitals in neighboring countries [19],[20]. Hence, this strain has a demonstrated ability to spread rapidly and to become predominant in the hospital environment, thereby replacing other MRSA strains that heretofore had been established for years [14]. At the same time, ST225 seems almost entirely restricted to the hospital environment, since it has not been reported from asymptomatic *S. aureus* carriage outside of hospitals and it is very rarely found among isolates from community-associated MRSA infections; in the latter, sporadic cases, close contacts to hospital patients or staff could not be excluded [21],[22].

We analyzed an international sample of MRSA type ST225 sequenced at 118,804 basepairs per isolate. Based on serial, time-structured samples of DNA sequences, we observed the accumulation of genetic diversity over a few years. By using coalescent (i. e., genealogy-based) methods, we calculated divergence times and reconstructed the pathogen's past demography. Our results are consistent with a scenario of a recent reduction in population size that has caused losses of genetic variation, and a subsequent population expansion of ST225 within Central Europe.

## Results/Discussion

### Variation within ST225

Isolates affiliated to ST225 – including both, MRSA and methicillin-susceptible *S. aureus* (MSSA) – display very limited genotypic and phenotypic variability based on contemporary, molecular typing techniques and antimicrobial resistance (Table S2). We used denaturing high-perfomance liquid chromatography (dHPLC) to screen for sequence polymorphisms at 269 genetic loci (predominantly randomly chosen housekeeping genes) from each of 73 *S. aureus* isolates (Tables S2, S3). Genome fragments investigated were scattered along the *S. aureus* chromosome and altogether comprised 4.2% (118,804 basepairs) of the genome (Table S3). Polymorphisms were ascertained through subsequent sequence analysis (Table S4a). All isolates belonged to sequence type ST225 or a single locus variant thereof (ST710) and had been isolated between 1994 and 2007 in the USA, the Czech Republic, Denmark, Switzerland, and Germany (Table S2). These analyses revealed 48 bi-allelic polymorphisms (BiPs; i. e., polymorphic sites at which exactly two alleles were observed), including 11 synonymous base substitutions in protein-coding regions, 26 non-synonymous substitutions, 10 substitutions in intergenic regions, and one insertion of a single nucleotide (Tables S4a, S5). The nucleotide diversity, π (the average number of nucleotide differences per site between sequences from two isolates), was 0.00001 for coding regions and 0.00003 for non-coding regions (Table S1). This level of diversity is extremely low; in a similar study on a global sample of *S. aureus* sequence type ST5 (the founder of clonal complex CC5), we recently discovered ten-fold higher diversity in both, protein-coding and intergenic regions [11]. In 70 ST225 isolates from Europe, we found 41 BiPs, which corresponds to 0.6 BiPs per isolate or 28 differences between any two 2.8 Mbp genomes. A similar level of divergence was recently reported for community-associated MRSA strain ‘USA300’, which, on average, displayed 35 differences between any two out of eight re-sequenced genomes [23]. The dN/dS value (the ratio of changes at non-synonymous sites to changes at synonymous sites) for protein-coding genes in ST225 was 0.77, hence, similar to the value found for ST5 [11]. This high proportion of non-synonymous substitutions is unlikely to represent a signal of selective pressures, but is a consequence of the dynamics of short-term evolution (i. e., evolution which occurs within a few years, see below) [11],[24],[25].

**Figure 1:**
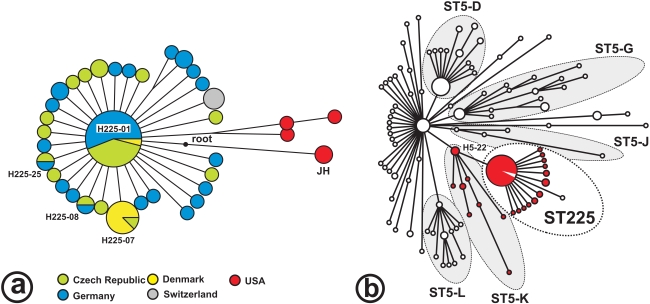
Radiation of ST225. Minimum spanning trees; the tree in Figure 1a is based on 269 loci investigated in 73 ST225 isolates, and the position of the JH strain was resolved based on published genome sequences for isolates JH1 and JH9 [28]. The ancestral node (‘root’) was determined by comparison to genome sequences from more distantly related isolates, including N315 (GenBank accession number BA000018), COL (CP000046), and MW2 (BA000033). Colours indicate the isolates' countries of origin. The tree in Figure 1b is based on 108 loci to show the relationship of ST225 to the previously reported ST5 radiation [11]. Haplotype H5-22 represents two ST5 isolates [11] and the JH strain. Red colour labels isolates harboring prophage ΦSaST5K.

The 48 BiPs enabled the discrimination of 36 haplotypes (i. e., unique combinations of BiP alleles) among the 73 isolates investigated (Table S2). There were only five parsimony informative sites (where derived alleles occurred in >1 haplotype), and four of these were found in isolates from the USA. Consequently, most of the variation was unique to individual haplotypes, and little phylogenetic structure was discerned among European ST225 isolates (Figure 1). The minimum spanning tree based on these BiPs shows a star-like radiation that is rooted at a hypothetical node representing the most recent common ancestor of ST225 and the JH strain (ST105; Figure 1a). This ancestor is affiliated to lineage ST5-K within the ST5 radiation (Figure 1b). It carries a number of derived alleles (listed in Table S4b) that distinguish it from ST5 haplotypes, in agreement with the previous presumption that ST5 was the ancestral genotype within the clonal complex CC5 [8].

### Recent origin and long-distance dissemination of ST225-MRSA

All ST225 MRSA isolates that we have investigated, including those from the US, carry a unique 997-basepair deletion in their SCC*mec* cassettes, which encompasses a 0.3-kb open reading frame (N315-SA0035) and the adjacent direct repeat unit (*dru*) locus. Deletions of the *dru* locus have rarely been reported [26],[27]. The presence of this characteristic feature in SCC*mec* indicates that the most recent common ancestor of the ST225 radiation had already been methicillin-resistant, which suggests that the entire radiation is younger than a few decades. The same *dru* deletion was present in the genome of the closely related JH strain (ST105, represented by isolates JH1 and JH9 [28], Figure 1), indicating it also existed in the common ancestor of ST225 and ST105, which, hence, already was methicillin-resistant. In addition, we found identical recombinase (*ccrB*) and helicase (*cch*) gene sequences in SCC*mec* from all ST225 MRSA isolates and from the JH genome (not shown), supporting the notion of a common origin. The *dru* deletion in international isolates also indicates a history of long-distance dissemination of MRSA, since sequence identity in this region would be unlikely if SCC*mec* elements had been imported repeatedly into locally endemic, methicillin-susceptible ST225 strains. Notably, our methicillin-susceptible isolates could not be distinguished from MRSA based on BiPs (Table S2), lending support to the presumption that they represent strains that have lost methicillin resistance together with parts of their SCC*mec* elements. Three of these MSSA carried SCC*mec* remnants in their chromosomes which we detected by PCR and sequencing, including the region with the *dru* deletion (Table S2). Even those isolates with no detectable traces of SCC*mec* may be secondary MSSA, however, since spontaneous, precise excision of SCC*mec* from the staphylococcal chromosome has been reported [29],[30].

There are several arguments why our American isolates of ST225 represent the ancestral population of the European clade. First, US ST225 isolates have been observed as early as 1994 (Table S2), whereas this clone was not encountered before 2000 in Europe. Second, considerable genetic diversity is observed among US isolates even from a single federal state (Wisconsin), with seven SNPs including four parsimony informative sites observed in only three isolates (Figure 1a). This is in stark contrast with the extremely low genetic diversity in European isolates, which suggests a recent population bottleneck (i. e., a brief reduction in population size) associated with the introduction of ST225 into Europe. A population bottleneck occurs, for example, when a small number of individuals founds a new population (‘founder effect’), and may result in a significant loss of genetic variation. Third, American ST225 carry a *spa* sequence (*spa* type t002) that is presumably ancestral to *spa* from European ST225 (t003, t045, t456, t1107; Tables S2a, S2b); the latter *spa* sequences may have arisen from t002 through deletions of individual repeat units, a frequent phenomenon during DNA replication, whereas the opposite (regain of unique repeats) appears less likely. *Spa* type t002 was also previously considered ancestral to other *spa* types based on the presence of a large number of single-repeat variants [31]. Finally, the ST225 radiation branches off from the ST5-K lineage (Figure 1b), to which the majority of ST5 isolates from the USA had been affiliated as reported in our previous study [11].

Taken together, we conclude that ST225 evolved from an MRSA that already carried the *dru* deletion in its SCC*mec* element. The novel clone spread to Europe somewhat later, where it rapidly became highly prevalent. The hypothesis of a single transmission event from the US is further supported by the low diversity and the monophyletic structure of the European ST225 radiation (Figure 1). However, current data do not preclude the existence of an ancestral ST225 population outside the US, although no such isolate has been observed so far.

### Temporal signal in DNA sequences and dates of divergence

**Figure 2:**
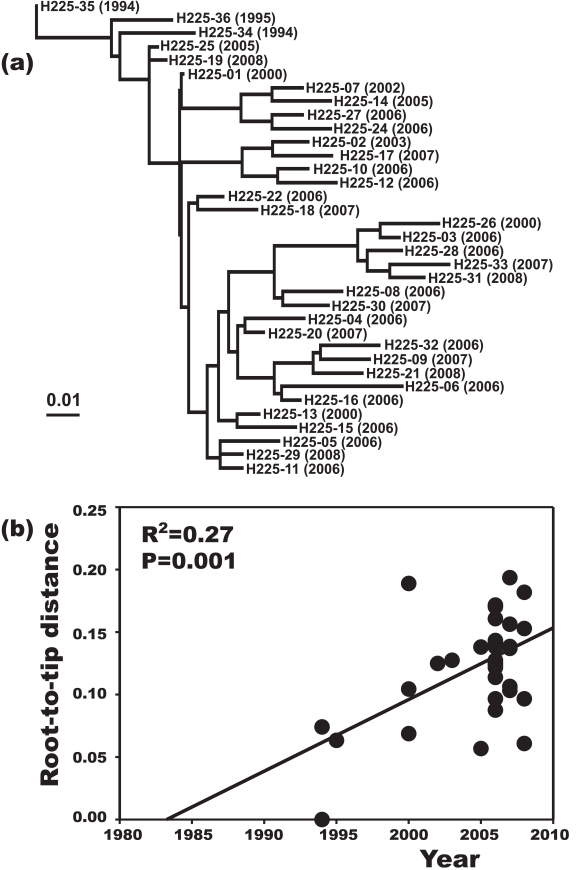
Increase of DNA sequence variation over the sampling time interval. Maximum-likelihood phylogenetic tree based on sequence variation among haplotypes (a). In this tree, each haplotype has a particular distance to the root. In (b), these root-to-tip genetic distances are plotted against sampling dates. The figure illustrates a positive correlation of divergence with sampling date, and, hence, a significant increase of DNA sequence variation over the sampling time interval.

**Figure 3:**
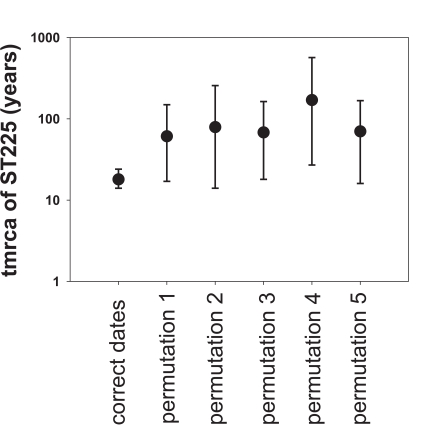
Effect of date permutation on the estimate of divergence time. Age of the ST225 clade (tmrca, time since the most recent common ancestor) and 95% confidence intervals on a log scale, determined by Bayesian phylogenetics analysis with the tips constrained by the correct dates and with the dates switched across isolates (permutations 1 to 5).

A plot of genetic distance from a common ancestor against sampling time gave a first indication of a measurable accumulation of DNA sequence variation over the sampling time interval (Figures 2a, 2b). Such sets of temporally spaced molecular sequences with a statistically significant number of genetic differences can be used to simultaneously estimate divergence times, temporal changes of population size, and nucleotide substitution rates by applying suitable statistical methods [32]. Based on the sequence variation ascertained, we calculated the age (divergence time) of ST225 by applying a Bayesian coalescent method of phylogenetic inference that incorporated a strict molecular clock model [33]. The relaxed molecular clock model was ruled out as it yielded a posterior distribution of clock rates showing negligible variation (with the standard deviation abutting zero), and was not statistically supported (likelihood ratio test, P = 0.99). Based on our dataset of 73 sequences, the most recent common ancestor of ST225 was estimated to 1990 (95% confidence intervals, 1987 to 1994) (Table 1). The age of the American ST225 clade coincides with the age of the entire ST225 radiation, and the European clade was estimated to have diverged since 1995 (95% confidence intervals, 1991 to 1999) (Table 1). Alternative tree priors (i. e., prior probability distributions) for the Bayesian analysis resulted in very similar time spans (Table 1). Sampling from the prior distribution, in contrast, resulted in hugely inflated divergence times (Table 1), suggesting our results are not mere artefacts reflecting the priors. While it may seem surprising that the little sequence variation discovered may suffice to calculate divergence times with such tight confidence intervals, a test based on random permutation of sampling times across isolates resulted in much older dates and much larger credible intervals (Figure 3), indicating our age calculations were based on a genuine signal in the data [34].

**Table 1:** Results of Bayesian analyses.

Tree prior	Clock model	Mean clock rate (95% confidence intervals)*	Mean tmrca of ST225 (95% confidence intervals)**	Mean tmrca of European radiation (95% confidence intervals)**
Bayesian skyline	relaxed (lognormal)	2.2×10^−6^ (1.3×10^−6^–3.1×10^−6^)	18 (15–22) years	13 (9–17) years
Bayesian skyline	strict	2.0×10^−6^ (1.2×10^−6^–2.9×10^−6^)	18 (15–23) years	13 (9–17) years
Constant size	strict	1.9×10^−6^ (1.0×10^−6^–3.1×10^−6^)	22 (15–29) years	16 (10–23) years
Exponential growth	strict	1.2×10^−6^ (5.8×10^−7^–1.8×10^−6^)	17 (15–21) years	15 (12–19) years
Logistic growth	strict	1.2×10^−6^ (5.7×10^−7^–2.0×10^−6^)	18 (15–23) years	15 (11–19) years
*empty alignment (sampled from prior):*				
Bayesian skyline	relaxed (lognormal)	12 (2.1^−14^–66)	5,746 (19–29,000) years	5,746 (17–29,000) years
Constant size	strict	49 (5–99)	1,100,000 (18–8.6×10^21^) years	1,100,000 (14–8.7×10^21^) years

*Clock rates are given in substitutions per nucleotide site and per year.

**The time since the most recent common ancestor (tmrca) is indicated in years before 2008.

### Demographic expansion

**Figure 4:**
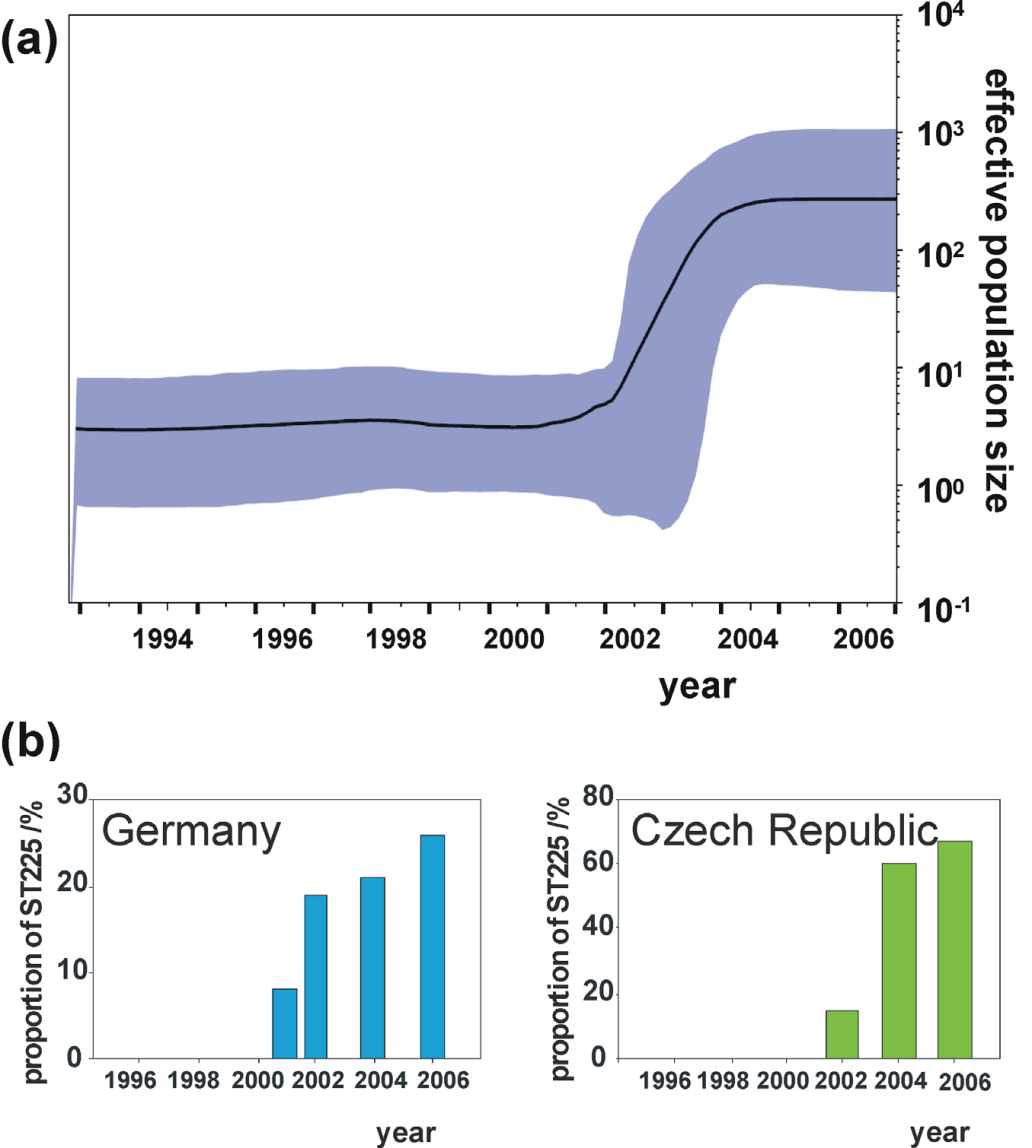
Effective population size through time in comparison to surveillance data. Bayesian skyline plot (Figure 4a), showing the effective population size of ST225 through time (black line), estimated from the concatenated dataset. The shaded area represents 95% confidence intervals. Proportional abundance of ST225 among MRSA in Germany and the Czech Republic (Figure 4b). Data for Germany are based on 2,000 MRSA isolates on average typed per year at the national reference centre for staphylococci. These isolates were received from all over the country and were associated with approximately 10% of all MRSA infections in Germany [66]. Data for the Czech Republic are based on 142 MRSA isolates recovered from blood samples in 13 different hospitals throughout the country.

The Bayesian skyline plot indicates a very sharp increase of the effective population size starting in 2001, with strong growth continuing for about three years and levelling off thereafter (Figure 4a). This demographic expansion, including the timing of events, is in full agreement with our observation of ST225 abundance in Central Europe (Figure 4b). This scenario is also consistent with a rampant expansion of the clone after its trans-Atlantic spread. The skyline plot (Figure 4a) was not unduly affected by heterogeneity in sample size per year, as indicated by the analyses of ten random subsamples of sequences from each year (Figure S1). However, we cannot exclude that population growth may have been more stochastic during the 1990s than is suggested by the current skyline plot (Figure 4a). To gain more detailed insights into the population structure during this time period, it would be particularly useful to investigate additional American ST225 isolates collected between 1990 and today, which are unfortunately not available at present. The composition of our sample seems to reflect the worldwide population structure of ST225 quite well, since many thousands of MRSA isolates have been genotyped to date in many countries, but no ST225 has ever been found outside Central Europe or the US. In a recent survey based on MLST typing of over 2,000 MRSA isolates sampled from Wisconsin, we did not find a single additional ST225 isolate (unpublished results of SKS). To probe the abundance of ST225 in Germany during the 1990s, we randomly chose 200 isolates from 1997 from the culture archive of the German national reference center for staphylococci and characterized them by *spa* typing and MLST. None of them was affiliated to ST225, suggesting that, at the time, the strain had been either absent or very rare in Germany.

### High rates of short-term evolution

The mean nucleotide substitution rate within ST225 was estimated at 2.0×10^−6^ substitutions per nucleotide site and year (95% confidence intervals, 1.2×10^−6^ to 2.9×10^−6^) (Table 1). This short-term evolutionary rate varied only slightly depending on clock model and choice of priors (Table 1), and was also largely confirmed by an alternative method based on a full likelihood model assuming a perfect star genealogy, which gave a rate of 1.1×10^−6^ (95% confidence intervals, 7.5×10^−7^ to 1.4×10^−6^). Even higher upper limits of substitution rates in bacteria have previously been estimated for *Neisseria gonorrhoeae* (4.6×10^−5^; [2]), *Helicobacter pylori* (4.1×10^−5^; [4]), and *Campylobacter jejuni* (6.6×10^−5^; [3]). In contrast to *S. aureus*, however, these three species are characterized by extremely high rates of homologous recombination, and, hence, part of the polymorphisms observed might have resulted from recombination rather than mutation [2],[3],[4]. Therefore, those reported rates had been considered maximal estimates; in the case of *H. pylori*, 100-fold lower rates were equally likely [2],[4].

Our rate for MRSA ST225 exceeds an evolutionary rate estimate that had been proposed for *Escherichia coli* in the past (3×10^−8^ substitutions per nucleotide site and year) by almost two orders of magnitude [35]. That previous estimate had been based on a laboratory mutation rate of 10^−10^ per nucleotide site and generation, and the assumption of approximately 300 generations elapsing per year [35]. Mutation frequencies measured *in vitro* (i. e., the average fraction of individuals carrying a particular resistance mutation in a laboratory culture) are very similar in *E. coli* and *S. aureus* [36],[37], suggesting comparable underlying mutation rates (the probability of a mutation to occur in each generation). While ‘mutator’ strains with elevated mutation frequencies have been described, they seem to be uncommon among clinical isolates [38]. A mutator phenotype for ST225 is also not supported by a comparison of whole genome sequences from 04-02981 (ST225, accession number CP001844, see below) and related isolates, including N315, JH1 and JH9 (Figure S2), and additional isolates (our unpublished data). In the genome from 04-02981, we detected no inactivating mutations in any genes involved in DNA replication fidelity, DNA repair mechanisms, or recombination, which are commonly associated with mutator phenotypes [38],[39]. Instead, it seems likely that the massive clonal expansion of ST225 was associated with short bacterial generation times and frequent transmission to new hosts. During rapid demographic expansions, both genetic drift and natural selection will be reduced, thus leading to an increase in the number of mutations segregating in a population, at least transiently [40].

Our results pointing to a rapid clonal evolution of *S. aureus* suggest that other bacteria may evolve faster than previously acknowledged. It must be considered, however, that observed molecular clock rates are time-dependent [41]. Generally, clock rates decline from initial mutation rates to long-term substitution rates, because the majority of mutations get eliminated with time due to genetic drift and selection [41]. Such rate curves have not yet been determined for bacteria. However, our results imply that recent divergence times of bacteria were possibly overestimated with dating based on the molecular clock rate suggested by Guttman and Dykhuizen [35],[42],[43]. It will be interesting to investigate short-term evolutionary rates in additional clones of *S. aureus* and other bacterial species. The time dependency of these rates may be established by comparing radiations at different levels of divergence.

Interestingly, the high rates of evolutionary change we found in MRSA caused the accumulation of DNA sequence variation within a few years, a feature that heretofore had been found only in highly recombinant (panmictic) gonococcus [2] and in rapidly evolving viruses [44]. Importantly, the time-structured sampling of DNA sequences within evolutionary timescales enables the application of sophisticated analytical methods, which opens up exciting prospects for investigations of the recent evolutionary history of bacterial pathogens, together with the forces that have shaped their spatial distribution.

### Dispersal among hospitals within Central Europe

**Figure 5:**
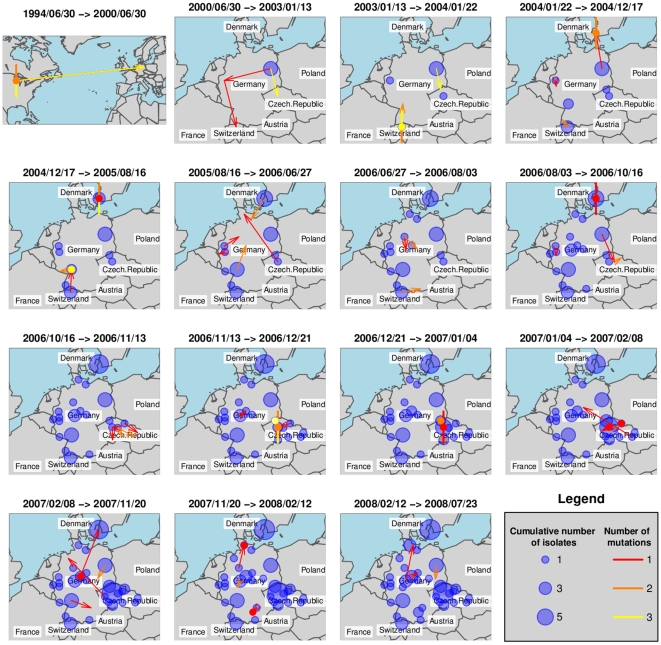
Ancestry-based scenario for the spatial spread of ST225. This figure shows the spatial spread of ST225, inferred for successive time windows by applying the *SeqTrack* algorithm. Each arrow represents an inferred ancestry, pointing from the ancestor to its descendent. Local ancestries are represented by colored dots for single isolates and dots with additional segments representing multiple ancestry events (one segment per isolate). Cumulative numbers of isolates are indicated with blue bubbles.

We have investigated ST225 isolates from four European countries (Table S2, Figure 1a) by reconstructing the most likely ancestry path between isolates to reveal the spatiotemporal dynamic of ST225 spread by applying the *SeqTrack* algorithm [45]. Interestingly, our results indicate that multiple haplotypes have been introduced into several countries (Figure 1a). Figure 5 represents the cumulative number of isolates from any location (bubbles) and the inferred ancestries (arrows) for successive time windows. Note that while Figure 5 represents the best-supported ancestry path given the sampled isolates, some ancestries might not correspond to actual transmission events, as the true ancestral population might not have been sampled. To avoid any overinterpretation of the results, we restrict our interpretation to the global pattern and some specific unambiguous features of the inferred ancestries.

After initial seeding into Europe, ST225 was transmitted to other locations in Germany and to additional European countries (the Czech Republic, Switzerland and Denmark). Some local ancestries (i. e., within the same city) are characterized by a relatively large genetic differentiation (Figure 5, colored dots) suggesting long-term persistence of ST225 within the same location. Another interesting feature of the reconstruction of the spatiotemporal dynamics of ST225 lies in the repeated transmission events between countries. For instance, one isolate from Denmark is assigned an ancestor from Germany with high likelihood (same genotype) at least three years after the first transmission from Germany to Denmark. The first transmission (Figure 5, time window 2004/01/22–2004/12/17) could be traced back epidemiologically to an index patient that had been transferred from a hospital in Germany into a hospital in Copenhagen, Denmark, in 2004, where the carried MRSA strain (haplotype H225-07; Table S2, Figure 1a) later caused an outbreak involving multiple patients and staff. Two additional isolates collected from the same hospital in 2006 and 2007 were affiliated to the same haplotype (Table S2), indicating the clone was still present three years after the initial outbreak. However, a second haplotype (H225-01) was indicated to have been introduced from Germany into Denmark (Figure 5, time window 2007/02/08–2007/11/20), and this is unlikely to be an artefact due to insufficient sampling within Denmark, as several local ancestry events were identified earlier within Denmark.

The *SeqTrack* results indicated 15 transfers among different countries within Europe (Figure 5). Considering the low informative diversity discovered, the limited number of isolates and countries investigated, and the short time span since emergence of ST225 has started in Europe, this number of detected international transfers of clones is very high. It indicates that cross-border spread of MRSA between the countries considered must have occurred frequently, and, more generally, that the turnover of hospital-associated MRSA is quite rapid even within a larger geographic region (Central Europe). Hence, the question arises how efficient geographic dissemination may be mediated. Abundant international travel will result in occasional hospitalization outside the country of residency, and potential subsequent cross-border patient transfers into the respective home countries. This route is exemplified by the introduction of haplotype H225-07 from Germany into Denmark, with the subsequent establishment of this clone in the hospital for several years. In addition, it is well documented that colonized health-care personnel may promote the spread of MRSA [46]. It is also possible that some spread of ST225 occurs outside of hospitals, even though the lack of community-associated isolates suggests the prevalence to be low [47]. Efficient containment of MRSA spread requires pro-active surveillance and eradication of colonization [46],[48].

### Clues from the genome sequence

It is unclear at present, if the success of particular MRSA strains such as ST225 may be due to fortuitous stochastic events or adaptive genetic changes. To reveal any genetic traits that distinguish ST225 from other strains of MRSA and may enable its massive expansion within short time, we sequenced the genome from one representative isolate, MRSA 04-02981 (haplotype H225-01, sequence accession number CP001844). We used both 454 (Roche) and Solexa (Illumina) technology, and closed the genome sequence by using long-PCR and Sanger-sequencing. The final genome sequence likely contains very few sequencing errors, if any, since the application of two independent sequencing approaches resulted in only six conflicting SNP calls. The genome from isolate 04-02981 was found to be co-linear with previously sequenced genomes from related isolates N315 (ST5) and JH1 (ST105) [28],[49]. There was no indication for the presence of any plasmids in isolate 04-02981.

Base substitutions were distributed evenly among genes of different functional categories (not shown). The effects that individual missense mutations may have on protein function are hard to predict in most cases. In the genomes from both, 04-02981 and the JH strain (including isolates JH1 and JH9), two open reading frames were truncated, one of which encodes an unknown, hypothetical protein (N315-SAS092) and another (N315-SA1092) encodes Smf, a protein that has been suspected to be associated with transformation competence. In addition, two open reading frames were uniquely truncated in the genome from 04-02981, encoding an adhesion factor (N315-SA1267) and the transcription regulator *norG* (N315-SA0104). The latter pseudogene initially appeared particularly interesting, because experimental disruption of this gene had been shown previously to result in a fourfold increase of *in vitro* resistance to beta-lactam antibiotics [50]. However, after applying a deletion-specific PCR (Table S6), we found that none of the other ST225 isolates in our collection had this deletion. Hence, truncation of *norG* is not a common trait of ST225, but rather is an idiosyncrasy of isolate 04-02981, which just happened to be the one we had chosen for genome sequencing.

### Complex phage dynamics

The genome of isolate 04-02981 contains a stretch of 44 kilobases of DNA that is inserted in a non-coding region downstream of the *sufB* gene (N315-SA0778), resulting in a duplication of the 67-basepair sequence upstream of the integration site. The inserted sequence is highly similar (sequence identity, 99.5%) to an as yet unnamed prophage previously found in the JH strain at the same genomic position [28]. It shares 50% or less overall sequence similarity to other phage genomes sequenced previously, including Φ11 from *S. aureus* NCTC8325 [28]. The prophage contains 68 predicted open reading frames, 19 of which encode proteins for basic phage functionality, and 49 of which have unknown functions. None of them has similarities to any known or presumed virulence factors.

By using PCRs targeting five specific regions (Table S6), we detected the presence of this prophage in all European ST225 isolates investigated and in other isolates affiliated to lineage ST5-K, but not in any other ST5 strains (Figure 1b). Thus, this particular prophage is specific to lineage ST5-K and its descendants, and we thus named it ΦSaST5K. Of note, prophage ΦSaST5K was not detected in any of our three ST225 isolates from the US, and, hence, it must have been lost by their common ancestor. There is a second phage – ΦN315 – in the genome of 04-02981, which it shares with isolate N315, an MRSA from Japan that is affiliated to lineage ST5-G [11]. In the JH strain, however, ΦN315 has been replaced apparently by another, dissimilar phage [28], and JH1 and JH9 harbor two additional prophages that have as yet not been seen in any other sequenced *S. aureus* genomes (Figure S2, Table S8b). This comparison of only three closely related MRSA genomes already points to the existence of complex phage dynamics, with varying apparent half-lives of prophages in their respective bacterial host chromosomes.

Our data indicates that several phages are associated to ST225 and its ancestral lineage, and may have played a role for its evolution. Bacteriophages have been suspected to promote the spread of pathogenic bacteria, by using various potential mechanisms. For example, phage genes may be directly implicated in immune evasion or virulence [51], or indirectly by affecting *in trans* the activity of bacterial genes outside the prophage, which in turn may enhance transmission or affect other fitness-related traits [52]. Alternatively, phages may possibly impact on competition between strains of staphylococci by driving lysis of bacterial cells that do not carry a related lysogenic phage.

### Prospects

We have shown that a strain of MRSA has accumulated measurable genetic change within an epidemiological timescale. The high short-term evolutionary rate in this MRSA enabled the estimation of divergence times and analyses of past changes in population size based on time-structured, serial DNA sequence samples, which heretofore had been possible only for highly recombinant gonococci and viruses. Moreover, ancestry reconstruction revealed the history of geographic spread of this MRSA at unprecedented detail. Confirmation of higher than expected short-term substitution rates in a wider range of bacterial pathogens, together with the tangible prospect of whole-genome sequences for large numbers of related isolates [53],[54] could prefigure a golden age for bacterial epidemiology. Presumably, bacterial pathogens will soon be amenable to detailed investigation of their recent evolutionary history and spread. At the same time, abundant polymorphisms will be discovered that will be useful for bacterial typing in epidemiological surveillance [55],[56],[57].

## Methods

### Bacterial isolates

Sources and properties of 73 isolates of *S. aureus* are listed in Table S2a. Susceptibilty to antibiotics was tested by using the broth microdilution method according to the DIN58940 instructions [58] and bacterial typing was performed as described previously [31].

### Genome sequencing

Draft genome sequences were generated and assembled commercially. 454 sequencing was performed on a GS FLX machine at 454/Roche in Branford, CT, USA, providing 32-fold average coverage of the staphylococcal chromosome and resulting in 42 initial contigs with >500 basepairs. Solexa sequencing was performed on a Genome Analyzer System at GATC in Konstanz, Germany, generating paired-end reads that were mapped onto the N315 genome sequence at 49-fold average coverage. Remaining gaps between contigs were closed by PCR using Hot Taq DNA polymerase (Peqlab, Germany) or long PCR using the Expand Long Template PCR System (Roche), respectively, and subsequent Sanger sequencing (primers in Table S7). Comparisons of contigs and genomes were performed by using Kodon software (Applied Maths, Belgium). After correcting sequences at contig ends and within repetitive elements, there were 468 sequence differences to N315, including base substitutions, insertions, and deletions (Tables S8A–S8D, Figure S2). Sequence differences to N315 that were shared between ST225 and the JH strain were considered correct since matching data had been generated in an independent study [28]. For insertions in the sequenced genome, we relied on 454 data, since they could not be detected among Solexa reads mapped against the N315 genome (Tables S8A–S8D). Gene annotation was performed automatically using the RAST server [59] and corrected manually using Kodon and Artemis software [60]. The annotated genome sequence from isolate 04-02981 was submitted to GenBank (accession number CP001844).

### Mutation discovery by dHPLC

Mutation discovery was performed as described previously [11]. PCR primers used for amplification and sequencing are listed in Table S3. A minimum spanning tree based on BiPs was constructed with Bionumerics 5.1. The ancestral node was determined by comparison to genome sequences from isolates N315 and JH1.

### PCR

PCR amplification of regions including the *dru* deletion, the four-basepair deletion within *norG*, SCC*mec* remnants, and prophage-specific fragments, respectively, were performed by using *Hot Taq* DNA polymerase (Peqlab, Germany) according to the manufacturer's instructions and by using the primers listed in Table S6.

### Regression of root-to-tip distances against sampling dates

Based on an alignment of polymorphic sites in protein-coding sequences, a maximum likelihood tree was calculated by using Treefinder software (available at www.treefinder.de), applying the HKY model of DNA substitution. Rooting of the tree and linear regression of root-to-tip distances against dates of first haplotype appearance was performed by using Path-O-Gen software (available at http://tree.bio.ed.ac.uk/software/pathogen/), and the significance of the correlation was determined with SigmaPlot 11.0 (SPSS).

### Likelihood ratio test

To assess whether nucleotide substitution rates in protein-coding sequences departed significantly from expectations under a strict molecular clock, we used a likelihood ratio test, based on a comparison of likelihood scores for maximum-likelihood trees calculated by using PAUP, with and without a molecular clock enforced. The statistical significance of the difference between likelihood scores was determined by assuming a chi-square distribution and *s*-2 degrees of freedom, where *s* was the number of sequences [61].

### Bayesian analyses

Evolutionary rates, divergence times, and Bayesian skyline plots were computed with the BEAST software (available at http://beast.bio.ed.ac.uk/) [62], using the HKY model of nucleotide substitution and a strict clock model (unless stated otherwise), with concatenated protein-coding sequences (108,261 basepairs) dated based on the year of isolate sampling, and with 10^8^ iterations after a burn-in phase of 10^6^ iterations. Markov chain Monte Carlo samples from three independent analyses were combined for estimation of posteriors, resulting in effective sample size values greater than 1,000 for all parameters. Various prior sets were used as indicated (Table 1). To test if date estimates were unduly influenced by prior assumptions, analyses were re-run (5×10^7^ iterations) on each of five datasets generated by randomly switching sampling dates across isolates. To sample from the prior distributions, analyses were run on an empty alignment. Further, to test if the resulting Bayesian skyline plot was confounded by temporal variation in sample size, we generated and analysed (10^7^ iterations) a series of datasets by subsampling from time classes and randomly drawing four isolates from each year.

### Nucleotide substitution-rate estimate assuming a star genealogy

For an alternative rate estimate, we used a full likelihood model assuming that demographic expansion was strong enough to result in a perfect star genealogy (i.e., without any coalescent events). To avoid violation of this assumption, we analysed protein-encoding loci (108,261 basepairs) from 58 European isolates exclusively, including only one isolate from each haplotype, except for the ancestral haplotype H225-01. Likelihood of the model for each locus was then given by the binomial probability of the number of mutations observed in all isolates, given the sum of the genealogical branch lengths for all isolates (i. e., date of isolate collection - date of expansion start) and a substitution rate parameter per locus and per year. A point multilocus substitution rate estimate (per nucleotide site and per year) and its 95% confidence interval were inferred based on the product of the above-described likelihood function for all loci, considering that all loci had a specific number of sites, were independent, and had a single, constant mutation rate. The procedure was written in *R* [63] and is available upon request to R. Leblois.

### Analysis of spatiotemporal dynamics of spread

The *SeqTrack* algorithm [45] was used to reconstruct the most plausible scenario for the spatiotemporal spread of the ST225 clone. This new method has been developed to study the dispersal and transmission of emerging pathogens during disease outbreaks, such as the 2009 swine-origin influenza A/H1N1 pandemic [45]. *SeqTrack* reconstructs the most likely ancestries among sampled strains using their genotype and sampling dates. This method differs fundamentally from phylogenetics in that it does not attempt to infer hypothetical (and unobserved) common ancestors, but rather seeks to reconstruct ancestries directly from the sampled isolates. Because of the low level of genetic variability in ST225 (most strains differ by a single nucleotide from each other), we used a maximum parsimony approach to infer ancestries. Thus, the most likely ancestry path was searched for by minimizing the number of mutations between ancestors and descendents. Whenever several strains were equally likely ancestors of the isolate under consideration, we retained the one that was geographically closest. All analyses were performed using the R software [63]. Raw genetic distances between isolates (in terms of number of point mutations) were computed using the *ape* package [64]. *SeqTrack* analysis was then run using the *seqtrack* function implemented in the *adegenet* package [65].

## Supporting Information

Figure S1 Negligible effect of sample size heterogeneity. Bayesian skyline plots based on analyses of ten random subsamples of DNA sequences from each year (2000 to 2008).
(1.64 MB TIF)

Figure S2 Phylogeny based on whole genome sequences. Maximum likelihood phylogenetic tree based on SNPs ascertained from whole genome sequences. Repetitive regions and mobile genetic elements were excluded. Branch designations A to D correspond to Tables S8A to S8D, which list genetic traits (base substitutions, insertions, deletions) that were derived along these branches. In the minimum spanning tree in Figure 4b, branches are shortened and branch B is collapsed entirely to a single point, because only approximately 4% of the genome was analysed in the larger set of isolates.
(1.25 MB TIF)

Table S1 Nucleotide variation.
(0.04 MB PDF)

Table S2 (a) Bacterial isolates. (b) Alignment of *spa* repeat successions.
(0.21 MB DOC)

Table S3 Genetic loci and PCR primers.
(0.07 MB PDF)

Table S4 Polymorphisms newly discovered in 269 genome fragments from 73 S. aureus isolates
(0.01 MB PDF)

Table S5 BiP alleles in each haplotype.
(0.46 MB DOC)

Table S6 Additional PCR primers.
(0.08 MB DOC)

Table S7 (a) Oligonucleotide primers for genome sequence gap closure. (b) Sequencing primers for genome sequence gap closure.
(0.20 MB DOC)

Table S8 Genomic differences between 04-02981, JH1/JH9, and N315. (a) Derived traits in the genome of 04-02981. (b) Derived traits in the JH strain. (c) Derived traits shared by 04-02981 and the JH strain.
(0.09 MB PDF)
