# Supplementary figures and images for: A Timescale for Evolution, Population Expansion, and Spatial Spread of an Emerging Clone of Methicillin-Resistant Staphylococcus aureus
Source: PLoS Pathog. 2010 Apr 8;6(4):e1000855. doi: 10.1371/journal.ppat.1000855 (PMC2851736; doi:10.1371/journal.ppat.1000855)

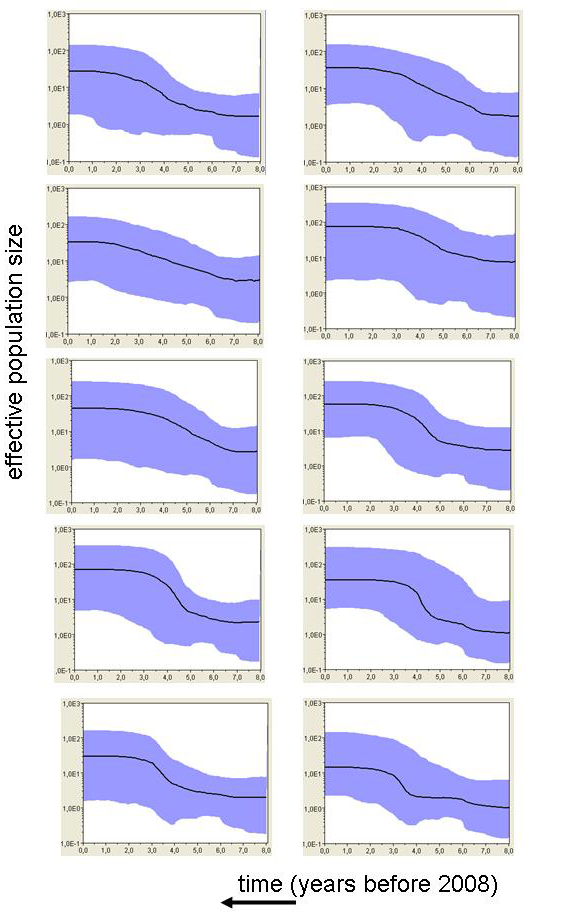

Supplement: Figure S1 — Negligible effect of sample size heterogeneity. Bayesian skyline plots based on analyses of ten random subsamples of DNA sequences from each year (2000 to 2008). (1.64 MB TIF) [file ppat.1000855.s001.tif]

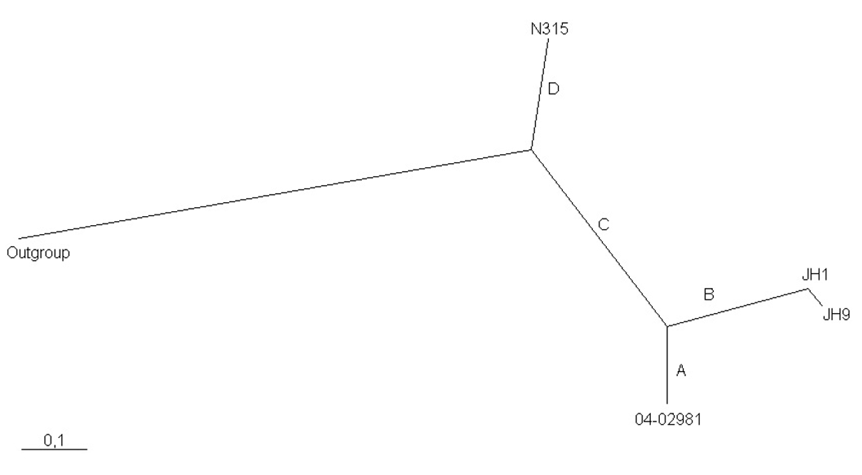

Supplement: Figure S2 — Phylogeny based on whole genome sequences. Maximum likelihood phylogenetic tree based on SNPs ascertained from whole genome sequences. Repetitive regions and mobile genetic elements were excluded. Branch designations A to D correspond to Tables S8A to S8D, which list genetic traits (base substitutions, insertions, deletions) that were derived along these branches. In the minimum spanning tree in Figure 4b, branches are shortened and branch B is collapsed entirely to a single point, because only approximately 4% of the genome was analysed in the larger set of isolates. (1.25 MB TIF) [file ppat.1000855.s002.tif]
