# Supplementary material for: A Timescale for Evolution, Population Expansion, and Spatial Spread of an Emerging Clone of Methicillin-Resistant Staphylococcus aureus
Source: PLoS Pathog. 2010 Apr 8;6(4):e1000855. doi: 10.1371/journal.ppat.1000855 (PMC2851736; doi:10.1371/journal.ppat.1000855)
Supplement: Table S1 — Nucleotide variation. (0.04 MB PDF) [file ppat.1000855.s003.pdf]

Table S1. Nucleotide variation*.

Locus category	basepairs	π	mean *dS*	mean *dN*	*dN/dS*
Protein coding	108261	0.00001 ± 0.0000000	0.0000169	0.0000130	0.77
Non-coding	7909	0.00003 ± 0.0000000	n. a.	n. a.	n. a.

* data based on 73 isolates; loci containing indels were excluded from these calculations;
π, the average number of nucleotide differences per site between sequences from two isolates; *dS*, the number of synonymous substitutions per synonymous site; *dN*, the number of non-synonymous substitutions per non-synonymous site;
