# Supplementary material for: A Timescale for Evolution, Population Expansion, and Spatial Spread of an Emerging Clone of Methicillin-Resistant Staphylococcus aureus
Source: PLoS Pathog. 2010 Apr 8;6(4):e1000855. doi: 10.1371/journal.ppat.1000855 (PMC2851736; doi:10.1371/journal.ppat.1000855)
Supplement: Table S2 — (a) Bacterial isolates. (b) Alignment of spa repeat successions. (0.21 MB DOC) [file ppat.1000855.s004.doc]

| **Table S2a. Bacterial isolates.** | | |  |  |  |  |  |  |  |
| --- | --- | --- | --- | --- | --- | --- | --- | --- | --- |
| **Isolate** | **Synonym** | **MLST** | **Spa type** | **SCC*mec **^2^** | **Country** | **City** | **Isolation date** | **Haplotype *^3^** | **Resistance *^4^** |
| 07-01590 | 4599-003A | ST225 | t003 | SCC*mec*II, *dru* deletion | Czech Republic | Praha | 24.12.2006 | H225-06 | PEN, OXA, ERY, CLI, CIP, MFL |
| 07-01591 | 85130 | ST225 | t003 | SCC*mec*II, *dru* deletion | Czech Republic | Strakonice | 04.01.2007 | H225-09 | PEN, OXA, ERY, CLI, CIP, MFL, GEN, SXT |
| 07-01592 | 833 | ST225 | t003 | SCC*mec*II, *dru* deletion | Czech Republic | Praha | 21.12.2006 | H225-01 | PEN, OXA, ERY, CLI, CIP, MFL |
| 07-01593 | B9883 | ST225 | t003 | SCC*mec*II, *dru* deletion | Czech Republic | České Budějovice | 13.11.2006 | H225-25 | PEN, OXA, ERY, CLI, CIP, MFL |
| 07-01594 | 81981 | ST225 | t003 | SCC*mec*II, *dru* deletion | Czech Republic | Praha | 13.12.2006 | H225-01 | PEN, OXA, ERY, CLI, CIP, MFL |
| 07-01595-1 | 126 | ST225 | t003 | SCC*mec*II, *dru* deletion | Czech Republic | Náchod | 09.01.2007 | H225-01 | PEN, OXA, ERY, CLI, CIP, MFL |
| 07-01596 | I158838 | ST225 | t003 | SCC*mec*II, *dru* deletion | Czech Republic | Plzeň | 10.01.2007 | H225-01 | PEN, OXA, ERY, CLI, CIP, MFL |
| 07-01597 | B10817 | ST225 | t003 | SCC*mec*II, *dru* deletion | Czech Republic | České Budějovice | 17.12.2006 | H225-10 | PEN, OXA, ERY, CLI, CIP, MFL |
| 07-01598 | 16377 | ST225 | t003 | SCC*mec*II, *dru* deletion | Czech Republic | Ústí nad Labem | 21.12.2006 | H225-11 | PEN, OXA, ERY, CLI, CIP, MFL, GEN |
| 07-01599 | 14137 | ST225 | t003 | SCC*mec*II, *dru* deletion | Czech Republic | Praha | 16.01.2007 | H225-01 | PEN, OXA, ERY, CLI, CIP, MFL |
| 07-01600 | 16521 | ST225 | t003 | SCC*mec*II, *dru* deletion | Czech Republic | Ústí nad Labem | 27.12.2006 | H225-01 | PEN, OXA, ERY, CLI, CIP, MFL, CMP |
| 07-01601 | 1485/HEM | ST225 | t003 | SCC*mec*II, *dru* deletion | Czech Republic | Třebíč | 03.11.2006 | H225-08 | PEN, OXA, ERY, CLI, CIP, MFL |
| 07-01602 | 2666 | ST225 | t003 | SCC*mec*II, *dru* deletion | Czech Republic | Náchod | 25.09.2006 | H225-01 | PEN, OXA, ERY, CLI, CIP, MFL |
| 07-01603 | 4599-042A | ST225 | t003 | SCC*mec*II, *dru* deletion | Czech Republic | Nový Jičín | 31.10.2006 | H225-12 | PEN, OXA, ERY, CLI, CIP, MFL |
| 07-01604 | 13443 | ST225 | t003 | SCC*mec*II, *dru* deletion | Czech Republic | Kolín | 15.09.2006 | H225-16 | PEN, OXA, ERY, CLI, CIP, MFL |
| 07-01605 | 3897 | ST225 | t003 | SCC*mec*II, *dru* deletion | Czech Republic | Praha | 11.11.2006 | H225-03 | PEN, OXA, ERY, CLI, CIP, MFL |
| 07-01606 | 1696 | ST225 | t003 | SCC*mec*II, *dru* deletion | Czech Republic | Hradec Králové | 08.02.2007 | H225-17 | PEN, OXA, ERY, CLI, CIP, MFL |
| 07-01607 | R13717 | ST225 | t003 | SCC*mec*II, *dru* deletion | Czech Republic | Praha | 29.11.2006 | H225-15 | PEN, OXA, ERY, CLI, CIP, MFL |
| 07-01608 | HEM4393 | ST225 | t003 | SCC*mec*II, *dru* deletion | Czech Republic | Zlín | 10.11.2006 | H225-01 | PEN, OXA, ERY, CLI, CIP, MFL |
| 07-01609 | N402168/1 | ST225 | t003 | SCC*mec*II, *dru* deletion | Czech Republic | Praha | 22.12.2006 | H225-01 | PEN, OXA, ERY, CLI, CIP, MFL |
| 07-03329 | TS 4152 | ST225 | t003 | SCC*mec*II, *dru* deletion | Czech Republic | Praha | 06.12.2002 | H225-07 | PEN, OXA, ERY, CLI, CIP, MFL |
| 07-03330 | TS 862 | ST225 | t003 | SCC*mec*II, *dru* deletion | Czech Republic | Ústí nad Labem | 10.02.2003 | H225-01 | PEN, OXA, ERY, CLI, CIP, MFL |
| 07-03027 | M 124 | ST225 | t003 | SCC*mec*II, *dru* deletion | Denmark | Copenhagen | 21.10.2004 | H225-07 | PEN, OXA, ERY, CLI, CIP, MFL |
| 07-03028 | M 145 | ST225 | t003 | SCC*mec*II, *dru* deletion | Denmark | Copenhagen | 30.11.2004 | H225-07 | PEN, OXA, ERY, CLI, CIP, MFL |
| 07-03029 | M 296 | ST225 | t003 | SCC*mec*II, *dru* deletion | Denmark | Copenhagen | 16.08.2005 | H225-07 | PEN, OXA, ERY, CLI, CIP, MFL |
| 07-03030 | M 597 | ST225 | t003 | SCC*mec*II, *dru* deletion | Denmark | Copenhagen | 18.08.2006 | H225-01 | PEN, OXA, ERY, CLI, CIP, MFL |
| 07-03031 | M 615 | ST225 | t003 | SCC*mec*II, *dru* deletion | Denmark | Copenhagen | 13.09.2006 | H225-07 | PEN, OXA, ERY, CLI, CIP, MFL |
| 07-03032 | M 704 | ST225 | t003 | SCC*mec*II, *dru* deletion | Denmark | Copenhagen | 14.02.2007 | H225-07 | PEN, OXA, ERY, CLI, CIP, MFL |
| 07-03033 | M 176 | ST225 | t003 | SCC*mec*II, *dru* deletion | Denmark | Copenhagen | 12.01.2005 | H225-07 | PEN, OXA, ERY, CLI, CIP, MFL |
| 07-03034 | M 126 | ST225 | t003 | SCC*mec*II, *dru* deletion | Denmark | Copenhagen | 27.10.2004 | H225-07 | PEN, OXA, ERY, CLI, CIP, MFL |
| 01-04209-1 | Va 248338 | ST225 | t003 | SCC*mec*II, *dru* deletion | Germany | Oberhausen | 20.12.2001 | H225-01 | PEN, OXA, ERY, CLI, CIP, MFL, CMP |
| 03-02595 | 98 | ST225 | t003 | SCC*mec*II, *dru* deletion | Germany | Heidelberg | 18.11.2003 | H225-01 | PEN, OXA, ERY, CLI, CIP, MFL, CMP, FUS |
| 04-00194-2 | 178 | ST225 | t003 | SCC*mec*II, *dru* deletion | Germany | Heidelberg | 20.01.2004 | H225-01 | PEN, OXA, ERY, CLI, CIP, MFL, CMP |
| 04-02981 | G 466 | ST225 | t003 | SCC*mec*II, *dru* deletion | Germany | Köln | 04.10.2004 | H225-01 | PEN, OXA, ERY, CLI, CIP, MFL, CMP |
| 05-00043 | MRSA 2122 | ST225 | t003 | SCC*mec*II, *dru* deletion | Germany | Freiburg | 17.12.2004 | H225-14 | PEN, OXA, ERY, CLI, CIP, MFL, CMP |
| 05-00941 | 369 | ST225 | t003 | SCC*mec*II, *dru* deletion | Germany | Heidelberg | 06.04.2005 | H225-25 | PEN, OXA, ERY, CLI, CIP, MFL, CMP |
| 05-01932-2 | 396 | ST225 | t003 | SCC*mec*II, *dru* deletion | Germany | Heidelberg | 29.07.2005 | H225-01 | PEN, OXA, ERY, CLI, CIP, MFL |
| 05-02010 | Y 3210-05 | ST710 *^1^ | t003 | SCC*mec*II, *dru* deletion | Germany | Saarbrücken | 03.08.2005 | H225-01 | PEN, OXA, ERY, CLI, CIP, MFL |
| 05-02212-1 | 8880 | ST225 | t003 | SCC*mec*II, *dru* deletion | Germany | Rendsburg | 30.08.2005 | H225-01 | PEN, OXA, ERY, CLI, CIP, MFL |
| 06-01100 | 235041 | ST225 | t003 | SCC*mec*II, *dru* deletion | Germany | Göttingen | 23.05.2006 | H225-05 | PEN, OXA, ERY, CLI, CIP, MFL |
| 06-01124 | 88 619B 115 | ST225 | t1107 | SCC*mec*II, *dru* deletion | Germany | Lübeck | 24.05.2006 | H225-28 | PEN, OXA, ERY, CLI, CIP, MFL |
| 06-01166-1 | 6279560 | ST225 | t045 | SCC*mec*II, *dru* deletion | Germany | Minden | 31.05.2006 | H225-27 | PEN, OXA, ERY, CLI, CIP, MFL |
| 06-01349 | 25331/06 | ST225 | t003 | MSSA, SCCmec remnants | Germany | Aachen | 27.06.2006 | H225-04 | ERY, CLI, CIP, MFL |
| 06-01400 | 91355 | ST225 | t003 | SCC*mec*II, *dru* deletion | Germany | Murnau | 04.07.2006 | H225-01 | PEN, OXA, ERY, CLI, CIP, MFL |
| 06-01506 | 600-132 | ST225 | t003 | SCC*mec*II, *dru* deletion | Germany | Bad Wildungen | 19.07.2006 | H225-24 | PEN, OXA, ERY, CLI, CIP, MFL |
| 06-01509 | 600-135 | ST225 | t003 | SCC*mec*II, *dru* deletion | Germany | Bad Wildungen | 19.07.2006 | H225-22 | PEN, OXA, ERY, CLI, CIP, MFL |
| 06-01602 | BA 432 | ST225 | t003 | SCC*mec*II, *dru* deletion | Germany | Blankenburg | 01.08.2006 | H225-32 | PEN, OXA, ERY, CLI, CIP, MFL |
| 06-01609 | 522-47 | ST225 | t045 | SCC*mec*II, *dru* deletion | Germany | Bad Wildungen | 03.08.2006 | H225-08 | PEN, OXA, ERY, CLI, CIP, MFL |
| 06-02150 | G9-15 | ST225 | t003 | SCC*mec*II, *dru* deletion | Germany | Rheinbach | 16.10.2006 | H225-01 | PEN, OXA, ERY, CLI, CIP, MFL |
| 06-03006-1 | 242070 | ST225 | t003 | MSSA | Germany | Göttingen | 18.12.2006 | H225-01 | PEN, ERY, CLI, CIP, MFL |
| 07-00265 | BA 5330 | ST225 | t003 | SCC*mec*II, *dru* deletion | Germany | Wernigerode | 25.01.2007 | H225-32 | PEN, OXA, ERY, CLI, CIP, MFL |
| 07-00952 | K021/197 | ST225 | t003 | MSSA, SCCmec remnants | Germany | Ingolstadt | 30.03.2007 | H225-20 | PEN, ERY, CLI, CIP, MFL |
| 07-01228 | 2451032 | ST225 | t003 | MSSA - | Germany | Göttingen | 02.05.2007 | H225-01 | PEN, ERY, CLI, CIP, MFL |
| 07-02236 | BK 203919 | ST225 | t003 | SCC*mec*II, *dru* deletion | Germany | Leipzig | 10.08.2007 | H225-18 | PEN, OXA, ERY, CLI, CIP, MFL |
| 07-03224 | 88 743B 1481 | ST225 | t003 | SCC*mec*II, *dru* deletion | Germany | Oldenburg | 20.11.2007 | H225-30 | PEN, OXA, ERY, CIP, MFL |
| 07-03397 | G 65905 | ST225 | t003 | MSSA - | Germany | Giessen | 12.12.2007 | H225-24 | PEN, ERY, CLI, CIP, MFL |
| 08-00002 | 17.12.07 - 258 | ST225 | t003 | SCC*mec*II, *dru* deletion | Germany | Augsburg | 07.01.2008 | H225-01 | PEN, OXA, ERY, CLI, CIP, MFL |
| 08-00035 | 17037 | ST225 | t003 | SCC*mec*II, *dru* deletion | Germany | Rendsburg | 07.01.2008 | H225-19 | PEN, OXA, ERY, CLI, CIP, MFL |
| 08-00123 | 550 | ST225 | t003 | SCC*mec*II, *dru* deletion | Germany | Rendsburg | 18.01.2008 | H225-01 | PEN, OXA, ERY, CLI, CIP, MUP |
| 08-00392 | AB 8063-200 | ST225 | t003 | MSSA, SCCmec remnants | Germany | Augsburg | 12.02.2008 | H225-21 | ERY, CLI, CIP, TGC, FUS, MUP, MFL |
| 08-00463 | BA 7276 | ST225 | t003 | SCC*mec*II, *dru* deletion | Germany | Wernigerode | 20.02.2008 | H225-31 | PEN, OXA, ERY, CLI, CIP, MFL |
| 08-01148 | 37276400 | ST225 | t003 | MSSA - | Germany | Riesa | 20.05.2008 | H225-29 | CIP, MFL |
| 08-01752 | 10011002 | ST225 | t003 | MSSA - | Germany | Kiel | 23.07.2008 | H225-22 | ERY, CLI, OTE, CIP, MFL |
| 08-02863 | H81-SAU-1219 | ST225 | t003 | SCC*mec*II, *dru* deletion | Germany | Berlin | 2000 | H225-13 | PEN, OXA, ERY, CLI, CIP, MFL |
| 08-02864 | H81-SAU-1085 | ST225 | t003 | SCC*mec*II, *dru* deletion | Germany | Berlin | 2000 | H225-01 | PEN, OXA, CIP, MFL |
| 08-02865 | H81-SAU-1176 | ST225 | t456 | SCC*mec*II, *dru* deletion | Germany | Berlin | 2000 | H225-26 | PEN, OXA, ERY, CLI, CIP, MUP, MFL |
| 08-02866 | H81-SAU-1082 | ST225 | t456 | SCC*mec*II, *dru* deletion | Germany | Berlin | 2000 | H225-26 | PEN, OXA, ERY, CLI, CIP, MUP, MFL |
| 07-03458 | ZH100 | ST225 | t003 | SCC*mec*II, *dru* deletion | Switzerland | Zürich | 22.01.2004 | H225-02 | PEN, OXA, ERY, CLI, CIP, MFL |
| 07-03459 | ZH16 | ST225 | t003 | SCC*mec*II, *dru* deletion | Switzerland | Zürich | 13.01.2003 | H225-02 | PEN, OXA, ERY, CLI, CIP, MFL |
| 07-03462 | ZH87 | ST225 | t003 | SCC*mec*II, *dru* deletion | Switzerland | Zürich | 24.11.2003 | H225-02 | PEN, OXA, ERY, CLI, CIP, MFL |
| 09-00824 | US-172 | ST225 | t002 | SCC*mec*II, *dru* deletion | USA | Marshfield, Wis. | July 1994 | H225-34 | PEN, OXA, ERY, CLI, CIP, MFL |
| 09-00825 | US-196 | ST225 | t002 | SCC*mec*II, *dru* deletion | USA | Marshfield, Wis. | December 1994 | H225-35 | PEN, OXA, ERY, CLI, CIP, MFL |
| 09-00826 | US-213 | ST225 | t002 | SCC*mec*II, *dru* deletion | USA | Marshfield, Wis. | March 1995 | H225-36 | PEN, OXA, ERY, CLI, CIP, MFL |
|  |  |  |  |  |  |  |  |  |  |
| JH1 (genome sequence) | | ST105 | t002 | SCC*mec*II, *dru* deletion | USA |  | 2000 | H225-00 | see Mwangi *et al*., PNAS 104: 9451 |
| JH9 (genome sequence) | | ST105 | t002 | SCC*mec*II, *dru* deletion | USA |  | 2000 | H225-00 | see Mwangi *et al.*, PNAS 104: 9451 |
|  |  |  |  |  |  |  |  |  |  |
| *^1^ ST710 is a single-locus variant of ST225 (allelic profile, 1-4-1-4-12-96-10) | | | | |  |  |  |  |  |
| *^2^ SCC*mec* elements carry a deletion of the *dru* region; | | | | |  |  |  |  |  |
| remnants of SCCmec were detected in MSSA by one or more of the following PCRs: SCC*mec* 3'-extremity/orfX, *dru* deletion, *ccrB*, *ccu*  *^3^ A 'haplotype' is a unique combination of BiP alleles on a staphylococcal chromosome.  ***^4^ Resistance against the following antibiotics was tested: penicillin G (PEN), oxacillin (OXA), phosphomycin (PHO), gentamicin (GEN), linezolid (LNZ), erythromycin (ERY), clindamycin (CLI), tetracycline (TET), tigecycline (TGC), vancomycin (VAN), teicoplanin (TPL), ciprofloxacin (CIP), mupirocin (MUP), moxifloxacin (MFL); | | | | | | | | |  |
|  |  |  |  |  |  |  |  |  |  |

| **Table S2b. Alignment of *spa* repeat successions *^1^.** | |
| --- | --- |
| ***spa* type** | **Repeat succession *^2^** |
| t002 | 26-23-17-34-17-20-17-12-17-...16 |
| t003 | 26-.........17-20-17-12-17-17-16 |
| t045 | 26-.........17-20-17-12-17-...16 |
| t456 | 26-.........17-...17-12-17-17-16 |
| t1107 | 26-.........17-20-17-12-......16 |
|  |  |
| *^1^ For nomenclature of *spa* types and repeats, see http://spa.ridom.de. | |
| *^2^ Dots indicate gaps in the alignment. | |
