# Supplementary material for: A Timescale for Evolution, Population Expansion, and Spatial Spread of an Emerging Clone of Methicillin-Resistant Staphylococcus aureus
Source: PLoS Pathog. 2010 Apr 8;6(4):e1000855. doi: 10.1371/journal.ppat.1000855 (PMC2851736; doi:10.1371/journal.ppat.1000855)
Supplement: Table S3 — Genetic loci and PCR primers. (0.07 MB PDF) [file ppat.1000855.s005.pdf]

Table S3. Genetic loci and PCR primers.

Locus	ORF	Annotation	Gene category	Left Primer	Right Primer	Left Position	Right Position	Product size
au200	SA0016	adenylosuccinate synthase	housekeeping (II)	TTCGTTTCGAAGGTAATTGG	CGTGCATTTTCAGGTAATTCTT	23216	23628	413
au201	SA0098	hypothetical protein, similar to aminoacylase	housekeeping (II)	TGAAGATGAAACGGCGAAAT	CCTGTTTTCATTGTGCTCAT	110529	110948	420
au202	SA0113	hypothetical protein, similar to ornithine cyclodeaminase (EC 4.3.1.12)	housekeeping (II)	AAACGCGTGTTTGTTTACGA	TTTTGTTGTTGTGCCTGTTGA	130244	130731	488
au203	SA0123	hypothetical protein, similar to UDP-glucose 4-epimerase (gale-1)	housekeeping (II)	TGGTTTTATTGGGTCGCATT	AATTTAATGTCGTCCGTTCG	142173	142618	446
au204	SA0171	NAD-dependent formate dehydrogenase	housekeeping (II)	TGCGCATGAATTACAACACA	TCTTCAATGCGTTTTTGTGC	194535	195002	468
au205	SA0182	hypothetical protein, similar to indole-3-pyruvate decarboxylas	housekeeping (II)	TTAGTTGCAATGAGCCATCA	TTCAGCAACAGCAGGATTTT	213236	213690	455
au206	SA0202	hypothetical protein, similar to gamma-glutamyltranspeptidase precursor	housekeeping (II)	TCTTGCGGCTAAAATTGGTAA	TGGCGCGAATATTTTTCAGT	239439	239855	417
au207	SA0220	hypothetical protein, similar to glycerophosphodiester phosphodiesterase	housekeeping (II)	TGAAAAGCGCATTGTTCTTC	TCCATTTTGACGCATTTTCA	263008	263471	464
au208	SA0256	6-phospho-beta-glucosidase	housekeeping (II)	CCGCAAATCAATTTGAAGGT	GCGCAAAATGCACAAAATAA	310060	310488	429
au209	SA0299	hypothetical protein, similar to carbohydrate kinase, PfkB family	housekeeping (II)	TTGCAGAAACATCAAATCCTG	TCATTTTTGGGGAAGAAACC	355123	355563	441
au210	SA0311	hypothetical protein, similar to trimethylamine dehydrogenase (EC 1.5.99.7)	housekeeping (II)	TCGAAATTTCCACACCATCA	CCGTAAACGCATCAACAAAA	369364	369789	426
au212	SA0346	hypothetical protein, similar to cystathionine beta-lyase	housekeeping (II)	TGACAGCACCAGCAATTAAA	ATTCGCTTTTGCTTTTGCAT	405743	406170	428
au213	SA0376	GMP synthase	housekeeping (II)	AAGTTGAACGTGCCAATGAA	TTGATCCCCAATTGCTTTATG	434800	435239	440
au214	SA0419	cystathionine gamma-synthase	housekeeping (II)	CAAACATGGCTTTGCGTTTA	ATGCCACCTGTTGAATTTGA	480802	481293	492
au215	SA0431	NADH-glutamate synthase small subunit	housekeeping (II)	AAGCGGGCATTACTTTCATT	AACGCATTGTTTGAACACCA	495821	496294	474
au218	SA0507	hypothetical protein, similar to N-acyl-L-amino acid amidohydrolase	housekeeping (II)	TCAAATTTGGGATTATGGTGTG	CAAACCTCATGAAACCATTGA	592338	592835	498
au219	SA0512	branched-chain amino acid aminotroansferase homologue	housekeeping (II)	AAATTTCACCTGCTGCACAA	TGCAACTTTTGCAAAGCCTA	599411	599841	431
au220	SA0537	hypothetical protein, similar to phosphomethylpyrimidine kinase, thiD homologue	housekeeping (II)	AAGCTTTGCCACCTTTAATGA	TGCAAGCAGATTTGAAAACG	631634	632116	483
au221	SA0646	hypothetical protein, similar to deoxyribodipyrimidine photolyase	housekeeping (II)	AAATGGTCGCAAAATGAAGC	TTCAATCCCCTGTTCAAAAA	741132	741557	426
au222	SA0697	hypothetical protein, similar to glycerate kinase	housekeeping (II)	CAGTTGCAAGCCAAATTGAA	TCGACTTGAAAAATCCGACA	795048	795547	500
au223	SA0730	2, 3-diphosphoglycerate-independentphosphoglycerate mutase	housekeeping (II)	CGGAAATTTTTGCGAACAGA	TTCGATTGCTTTGATTGTCG	836703	837148	446
au224	SA0791	hypothetical protein, similar to glycerate dehydrogenase	housekeeping (II)	TGACGTTGTTATGTGGCAAAA	TTAAACCCTTGCAACCTTCTT	896974	897414	441
au225	SA0859	thimet oligopeptidase homologue	housekeeping (II)	TACTGAATTGCGCAAAGAATTG	ATGCAACTTCAGCGACAAAA	974341	974741	401
au226	SA0945	dihydrolipoamide S-acetyltransferase component of pyruvate dehydrogenase complex E2	housekeeping (II)	AAAGAAATCGCAGCTGAACAA	TTTTGAGCAATACGGCCAAT	1073758	1074161	404
au227	SA1044	dihydroorotase	housekeeping (II)	ATGAAGGGAAACGCAGTAAA	CAATTCCGAATGGTGCTTTT	1181330	1181731	402

au228	SA1115	riboflavin kinase / FAD synthase ribC	housekeeping (II)	CAATGGCATTCGGATTTTTC	CATTCGCTTTTTGCAACTCA	1264780	1265262	483
au229	SA1177	transketolase	housekeeping (II)	TTGGTTCACCGAATAAAGCA	TGGATTTGTTTGAACCAGCA	1339372	1339785	414
au230	SA1226	asd, aspartate semialdehyde dehydrogenase	housekeeping (II)	AAAAAGCTGGTGCAATCGTT	TCGTTTCTTTGTCAAGCGTTA	1401265	1401747	483
au231	SA1308	30S ribosomal protein S1	housekeeping (II)	ATGCACCAAAGTTTGCCAAT	TTCCGGCTTCACTAATTTCAA	1513993	1514478	486
au232	SA1412	oxygen-independent coproporphyrinogen oxidase III	housekeeping (II)	TTCATGGCCATCTAATGCAA	CATTTGAAGCAAATCCTGATGA	1617553	1618016	464
au233	SA1496	glutamyl-tRNA reductase	housekeeping (II)	ATTTCCCCTGCACCAATAAT	CGAATTGCCCATGAAGATTT	1704604	1705096	493
au234	SA1545	D-3-phosphoglycerate dehydrogenase	housekeeping (II)	TGCAGTGAATGCACCTAAAA	TAACAATGCGCCAGTTTTTC	1766721	1767216	496
au235	SA1681	glutamate-1-semialdehyde aminotransferase	housekeeping (II)	TTCAAGAACAAGCAGCCAAA	TGAGGCATTACCATTCCAAA	1926891	1927314	424
au236	SA1814	hypothetical protein, similar to succinyl-diaminopimelate desuccinylase	housekeeping (II)	ATGTTGCACCCATGTTCAAA	CATCGATTTGATGTGCCATT	2053853	2054311	459
au237	SA1924	hypothetical protein, similar to aldehyde dehydrogenase	housekeeping (II)	GCAAATGCTGCTGCTAATTTT	TTAATGGCGAATGGGTAGAAA	2173216	2173675	460
au238	SA1991	6-phospho-beta-galactosidase	housekeeping (II)	TTTTGTTGGCAATGCGTGTA	ATTTCAATTGCATGGTCACG	2263407	2263853	447
au239	SA2120	hypothetical protein, similar to amino acid amidohydrolase	housekeeping (II)	GCAATTGCTTTATGCGTCAAT	TCAAAACAGGGGCAATTACA	2383969	2384377	409
au240	SA2214	adenosylmethionine-8-amino-7-oxononanoate aminotransferase	housekeeping (II)	ACCCTGCGATTTCATCATTT	TAAAATTGCCCATTCTACGC	2485384	2485811	428
au241	SA2304	fructose-bisphosphatase	housekeeping (II)	TTTCGAAATGGAAGAACGTC	ATCGCGCGTTTACCAAATAA	2589863	2590336	474
au242	SA2410	nrdD, anaerobic ribonucleoside-triphosphate reductase	housekeeping (II)	ACTCGTGCGCAAATTTCTTT	ACGGATCGTTTTTGTCGTTT	2701426	2701886	461
au243	SA2470	hypothetical protein, similar to histidinol dehydrogenase	housekeeping (II)	TGTTTGAATCGACGCATGTT	TTGCTGCATTGACTTATGGAA	2781905	2782326	422
au244	SA0177	argJ, arginine biosynthesis bifunctional protein homologue	housekeeping (II)	TGGCAAATCACCAAGTTGAA	TGCGTTAATACGCACATAATCA	206898	207388	491
au245	SA0240	sorbitol dehydrogenase homologue	housekeeping (II)	TTGAAAGTGCGAATGACGTT	CAAATATTCGTGCCCATTGA	291411	291896	486
au247	SA0361	truncated hypothetical protein, similar to phosphoglycerate mutase; Gpm3p	housekeeping (II)	CATGGCGAATCAAAATCGAA	CCATCAAACAACGAATGACAA	418661	419144	484
au248	SA0527	probable glucosamine-6-phosphate isomerase	housekeeping (II)	AAGAGATGGCATTTAATCAGCA	AATGGCTTGCTTTGGAACAT	622181	622659	479
au250	SA0572	hypothetical protein, similar to esterase/lipase	housekeeping (II)	TTTAAACGATCACGTTGAGGA	AAAGGATCATGTTCCCCATT	665507	665947	441
au251	SA0671	hypothetical protein, similar to urea amidolyase	housekeeping (II)	GAAATTGTGCCATCAGAATCA	TTTAAGCGTGCGAAATCTGA	764197	764696	500
au252	SA0821	argH, argininosuccinate lyase	housekeeping (II)	TGCATTTTCAACTGGCTCAT	TTTGACGTCGTTTCAAGCAA	926012	926505	494
au253	SA0896	menD, menaquinone biosynthesis protein	housekeeping (II)	GGTTAGAAAAATGGCAATGCT	GCCAAACAACCGTTCAAAAT	1017529	1017968	440
au254	SA0924	purN, phosphoribosylglycinamide formyltransferase	housekeeping (II)	CGATTTTTGCATCAGGTTCA	TTTTGAATCGTCCGGTCTAA	1049523	1050001	479
au255	SA1089	sucD, succinyl-CoA synthetase	housekeeping (II)	AAAAATGAAACTGGGGCAAC	CCGATTTCACCAATCATAACAA	1232627	1233078	452
au256	SA1150	glnA, glutamine-ammonia ligase	housekeeping (II)	TGAAAAAGGGGAACCAACTT	AATCCGCGTGCATTTTTAAG	1307593	1308024	432
au257	SA1366	glycine dehydrogenase subunit 1	housekeeping (II)	TCGCATTGCAAGCAAGAATA	CAATTGAACCGTAAAAGTTTGG	1575017	1575507	491
au258	SA1487	folC, folylpolyglutamate synthase	housekeeping (II)	TTTACGTCGCCGTTTATTGA	CAATTGGCTTTGCATGTTGT	1693267	1693720	454
au259	SA1524	malate dehydrogenase homolog	housekeeping (II)	CGCAATTTTTGAAGGACGTT	CTTTAGCAACATTACGAGCAA	1739355	1739845	491
au260	SA1554	acsA, acetyl-CoA synthetase	housekeeping (II)	ATTGTTGGGCCGTTATTTGA	TTTGTAGCGCCATTTAACCA	1781281	1781729	449
au261	SA1585	proline dehydrogenase homolog	housekeeping (II)	TTTAGGGGAATTTGTCGGTA	GAAATTGCGTGCGTTTAACA	1824023	1824497	475

au262	SA1669	citG, fumarate hydratase, class-II	housekeeping (II)	ACACATTTACAAGATGCAACG	TGCAACCATTGTTAACATTTCA	1905900	1906352	453
au263	SA1735	manganese-dependent inorganic pyrophosphatase	housekeeping (II)	CCTGAAATTGCCGGTTTAAT	TTTTTCGAGAAACAACACCAGA	1987761	1988244	484
au264	SA1864	3-isopropylmalate dehydratase large subunit	housekeeping (II)	TGCATTTGGAATTGGAACAA	TGATTTCAGGGAATGGTTCA	2105510	2106003	494
au265	SA1965	glmM, femD, phosphoglucosamine-mutase	housekeeping (II)	ACAAAGCGCTTGAACAAGAA	TTGTGCAAATCTTTCAGCATC	2225913	2226346	434
au266	SA2008	alsS, alpha-acetolactate synthase	housekeeping (II)	AAAATCATTTCTTCGGTCGTG	TGAAATTACGTGCCATCCAA	2280271	2280729	459
au267	SA2071	moeB, molybdopterin biosynthesis protein moeB	housekeeping (II)	TGCGTTGATTATTGGTATGG	TTTAATGCTGGCAATTGTGG	2332305	2332760	456
au268	SA2099	hypothetical protein, similar to monooxygenase	housekeeping (II)	ATCCGGATTGTGCAAAAGAA	TGGGCAATTTTACCGATTTT	2359332	2359800	469
au269	SA2240	hypothetical protein, similar to para-nitrobenzyl esterase chain A	housekeeping (II)	TGCACGCAATAAAGCACAAT	TGCAAATTCAGCAAGCCATA	2517061	2517511	451
au270	SA2260	hypothetical protein, similar to glucose 1-dehydrogenase	housekeeping (II)	TGCGTGGGACAATTTTAATG	TTCCACGAATCATCACTTAAC	2537713	2538172	460
au271	SA2327	hypothetical protein, similar to pyruvate oxidase	housekeeping (II)	ATGTACGATGCCAAAATGGA	TCATCCGGCAAGATTGTTTT	2612925	2613400	476
au272	SA2348	crtN, squalene desaturase	housekeeping (II)	TTAATGCCAGATTTTGCACCT	TTTCGCATGATACGTTTGCT	2638403	2638864	462
au273	SA2393	hypothetical protein, similar to 2-dehydropantoate 2-reductase	housekeeping (II)	AGACACGCCAAAACAATAACA	ATGCATGATTGCAACTGTCT	2680864	2681340	477
au274	SA2490	pckA, phosphoenolpyruvate carboxykinase	housekeeping (II)	TTTCAACTTTCGACGACACAA	TGCTTTAAAATGTGGTGCAGA	1844849	1845280	432
au275	SA0969	hypothetical protein, similar to glycerophosphoryl diester phosphodiesterase	housekeeping (II)	CGCACCACAAATTATGTTTGA	TTCGGGATTGTTTGTTTGAA	1099466	1099873	408
au276	SA1334	hypothetical protein, similar to pyrroline-5-carboxylate reductase	housekeeping (II)	AACACGCATCAAACCACATA	TTTTTCTTAATTGAGCCATGC	1540736	1541178	443
au277	SA2439	sasF, conserved hypothetical protein (Cooper + Feil)	hypothetical (V)	AATGCCTCAACGCAACATTA	CAACGATTTGGCGATTTGAT	2743068	2743554	487
au278	SA1619	hypothetical protein (Cooper + Feil)	hypothetical (VI)	AAAAGCAAATGACACCACAAGA	TTGCATGGTTATCATGTTTTTC	1853647	1854096	450
au279	SA0740	hypothetical protein (Cooper + Feil)	hypothetical (VI)	TTGGAACATTTATTATGGCTTT	ACGGTCGATTTGACCTTTTA	847062	847504	443
au280	SA0817	hypothetical protein, similar to NADH-dependent flavin oxidoreductase (Cooper + Feil)	cell envelope (I)	TGAAGAAGTGCTTAAAGCGAAA	AATTTCATCTTCCCGTCCAT	921075	921477	403
au281	SA2445	hypothetical protein (Cooper + Feil)	hypothetical (VI)	TTAAAATGGCCGGATGATGT	TTTCGCTGGAATATGATTGG	2753158	2753616	459
au282	SA2121	hutI, imidazolonepropionase (Cooper + Feil)	housekeeping (II)	CCCATTTAACATTTGGTGGA	GCTTCTTTGGCTTTTTGCAT	2385678	2386152	475
au283	SA1843	agrC, accessory gene regulator C (Cooper + Feil)	information pathways (III)	ATTGGTCTACGCGCTTATTT	TTGTCTGCATTATCAGCAATTT	2080947	2081401	455
au284	SA0015	dnaC, replicative DNA helicase (Cooper + Feil)	information pathways (III)	AAAGTTGCAACGCATGAAGA	TTGCTCAATCGAACCAGATT	21445	21939	495
au285	SA0119	hypothetical protein, similar to diaminopimelate	housekeeping (II)	TTGAATGTGGGCGCTTTATT	TCCTCCTTTGTTTGCGTTAAA	138812	139214	403
au286	SA0223	hypothetical protein, similar to UDP-glucose 4-epimerase	housekeeping (II)	TGCAATCGTTTGAGAAGCAA	TGGTCGGTTCAAAGATGAAA	267449	267872	424
au287	SA0416	hypothetical protein, similar to carboxylesterase	housekeeping (II)	ATTAAAATTGGCGCAGCACT	GCATTGAAAAAGCGAATAACA	477346	477781	436
au288	SA0545	phosphotransacetylase	housekeeping (II)	GCGCAAAAACTTGATCTTGA	TGCCAAAGCTTAATGCTGATT	638508	638919	412
au289	SA0656	probable N-acetylglucosamine-6-phosphate deacetylase	housekeeping (II)	ATTGTTGCAATTGGAGAAGG	ACGTTTCAAGCGCTTCTTTT	750368	750863	495
au290	SA0843	3-oxoacyl- synthase	housekeeping (II)	TATGGCAATTGCAGGTTTCA	TTGCTTCAATTCCACCTGTT	956696	957156	461
au291	SA0881	hypothetical protein, similar to nucleotidase	housekeeping (II)	GGCATCAAAACGTTGGATTA	GCGAAAATGGATGTGTTTGA	1001452	1001936	485
au292	SA0902	HisC homolog	housekeeping (II)	TGGAACGATTGCTAAATGTGTT	TCCTTCGCTGAATTTGAAGA	1023317	1023721	405

au293	SA0958	myo-inositol-1(or 4)-monophosphatase homolog	housekeeping (II)	TTTATGGATTTGCCCAAGGA	TTCAATGGTTTCAAGGGTTG	1084827	1085247	421
au294	SA1121	hypothetical protein, similar to processing proteinase	housekeeping (II)	AAGAAATGGCCAACCGATAA	TTGCCGACAACATAAACAGAA	1273973	1274433	461
au295	SA1163	aspartate kinase homolog	housekeeping (II)	AACAAAAATACCCGCTTCCTT	AAAGTTTCCAAATTCGGTGGT	1324852	1325298	447
au296	SA1199	hypothetical protein, similar to anthranilate synthase	housekeeping (II)	AAATCCAACCATTCATGCCTA	TTCGCTGCATTCTTTTGGAT	1373094	1373518	425
au297	SA1298	3-dehydroquinate synthase	housekeeping (II)	TTGCACTACAATATCGCCAAA	TGAATCAACGACGCAAGAAA	1504486	1504930	445
au298	SA1349	dihydrolipoamide dehydrogenase	housekeeping (II)	TTGATCCAACATGTGCCAAT	TGGTGTTATCGGAATGGAAT	1559232	1559653	422
au299	SA1377	glucokinase	housekeeping (II)	TCAAATTGAGCAGGTGCAAA	GCGCAGAAATAGGTCATTTT	1582274	1582676	403
au300	SA1652	uroporphyrinogen decarboxylase	housekeeping (II)	AAGCAAACCATGTTGCTTCA	TTCGCAACCAGAATATCGAA	1886778	1887220	443
au301	SA1685	hypothetical protein, similar to A/G-specific adenine	housekeeping (II)	AATTGCCACATACCATGCAA	TCCCAAAGATCCTGATCAATTT	1932656	1933123	468
au302	SA1724	adenylosuccinate lyase	housekeeping (II)	ACGGAAAGGTGTTTTCGTTT	AAATTTGCCGTTGAAATTCG	1974092	1974559	468
au303	SA1749	hypothetical protein, similar to aspartate transaminase	housekeeping (II)	GCCCAAAGCTTAAATGAACAA	ATGTTTGAAGGTTTGCACCA	2001475	2001949	475
au304	SA1858	dihydroxy-acid dehydratase	housekeeping (II)	TTGGTTTGACGGCGTATTTT	ATGCATCATCAATTGCTTCG	2097921	2098372	452
au305	SA1874	alanine racemase	housekeeping (II)	TCGCATTGATGACCATTTACA	CTTGTATTTGAAGGCGTGTTT	2122432	2122892	461
au306	SA1963	mannitol-1-phosphate 5-dehydrogenase	housekeeping (II)	TTGCAGACGTCAATGAAGAAA	TCTTTTTCAACAACCCATTCG	2216805	2217249	445
au307	SA2001	hypothetical protein, similar to oxidoreductase	housekeeping (II)	TTCTTCTTGTTGATGGAATGGA	AAACAGCAGAAGCGGTTAAA	2272199	2272623	425
au308	SA2140	hypothetical protein, similar to esterase	housekeeping (II)	TGCCATTAATTGGCGATATG	TTCCATTGGAGATTGTTGGA	2404832	2405246	415
au309	SA2188	nitrite reductase	housekeeping (II)	TGCATTTCCATCAACCATTC	TAACCGCATCATGCTTTCAA	2459852	2460274	423
au310	SA0112	hypothetical protein, similar to cysteine synthase	housekeeping (II)	TTGCAAAGGTGCAAGAACTG	CTGCTCAATCGCTGCAATAA	129169	129617	449
au311	SA0169	hypothetical protein, similar to acyl-CoA dehydrogenase family protein	housekeeping (II)	AAGCAGTTTGCGGCAACTAT	ACCCCTTCAACTCTGCTTCA	192914	193334	421
au312	SA0178	argC, N-acetylglutamate gamma-semialdehyde dehydrogenase	housekeeping (II)	CTGTAGGGAAACAGCCAGGA	TAGGTATCGTTGGCGGTAGC	208717	209157	441
au313	SA0225	hypothetical protein, similar to glutaryl-CoA dehydrogenase	housekeeping (II)	AGCGCGTCCAATTACTAAGG	GCGGATCGCTTACAAAACAT	270927	271391	465
au314	SA0225	hypothetical protein, similar to glutaryl-CoA dehydrogenase	housekeeping (II)	CAATGCGAAGTGCGATTTTA	CAAACGCCAAAATGCCTAGT	271436	271873	438
au315	SA0258	rbsK, probable ribokinase	housekeeping (II)	CGCCGTGTTTTTGTACAGTG	GAAGTACCCATCCCGGCTAT	312996	313443	448
au316	SA0317	hypothetical protein, similar to dihydroflavonol-4-reductase	housekeeping (II)	TTGCAATTTTTGGACCATCA	TTCTTCTTGTGTCGCAATCG	375150	375571	422
au317	SA0344	5-methyltetrahydropteroyltriglutamate- homocysteine methyltransferase	housekeeping (II)	GCGTTTTCAAACCACAGTCA	GCACTTGAAGCTGCAGGAAT	401246	401678	433
au318	SA0344	5-methyltetrahydropteroyltriglutamate- homocysteine methyltransferase	housekeeping (II)	ATTGCACCCAACCGAATTTA	ATTGCGTCGCCTATTGAATC	401901	402345	445
au319	SA0344	5-methyltetrahydropteroyltriglutamate- homocysteine methyltransferase	housekeeping (II)	TTGCCCATACATTACGACCA	ACGTTAACGCTCACCCTGTC	402524	402944	421
au320	SA0347	hypothetical protein, similar to cystathionine gamma-synthase	housekeeping (II)	TTTGACGGTTACGACACCTG	GCATGGTATTGCATCATTCG	406844	407288	445
au321	SA0375	inositol-monophosphate dehydrogenase	housekeeping (II)	CTATGGCTCGTCAAGGTGGT	ATACCAATTGCTGCGGCTAC	433198	433696	499
au322	SA0375	inositol-monophosphate dehydrogenase	housekeeping (II)	GAAGCGGGTGCAGATATTGT	TAAGCCGTACGTCCTTCGAT	433884	434287	404
au323	SA0418	cysM, cysteine synthase homologue	housekeeping (II)	TGCGTGCAGGTCAAACTATT	ACATTGCACGTGATGTTGCT	479873	480276	404

au324	SA0508	hypothetical protein, similar to glycine C-acetyltransferase	housekeeping (II)	GTGCAAACGGACCAGAAATC	TTTCGCACGTAAATCATCCA	593690	594090	401
au325	SA0511	hypothetical protein, similar to UDP-glucose 4-epimerase related protein	housekeeping (II)	CGATTCAGCAACCTACGACA	TAGGATCAAATCCCCATTCG	598347	598835	489
au326	SA0533	hypothetical protein, similar to long chain fatty acid CoA ligase	housekeeping (II)	TATGAAATCACCGGTGCAAA	CGACCGGAACTTAAAGCAAA	627507	627914	408
au327	SA0534	acetyl-CoA c-acetyltransferase	housekeeping (II)	GAAATGAGCGACCCATCAAT	GTGCACTGAACGCTTCGTTA	629153	629651	499
au328	SA0548	product mevalonate diphosphate decarboxylase	housekeeping (II)	TTTGCAGAATGGGAAAAAGG	CCCGCATCCATCGTAAAATA	642124	642530	407
au329	SA0658	hypothetical protein, similar to plant-metabolite dehydrogenases	housekeeping (II)	AGGCTTCACTAGGACGAGCA	AGGCATCCATGCAGTCACTT	753411	753820	410
au330	SA0686	nrdE, ribonuceloside diphosphate reductase major subunit	housekeeping (II)	CGTGGTGAAGCAATTAAAGGA	ATTTCACGCGCATTCTTTTT	784098	784498	401
au331	SA0731	enolase	housekeeping (II)	ATGTTTACGCTCGCGAAGTC	GGAATGCAATTGGAGCATCT	837573	838049	477
au332	SA0776	aminotransferase NifS homologue	housekeeping (II)	AAAATGCTTGGACCAACAGG	CCCAGCTCTAACCGCTACAC	883076	883486	411
au333	SA0823	glucose-6-phosphate isomerase A	housekeeping (II)	ATGATGATTGGTGCTGCAAA	CCACCATCAGTATGCGCTAA	929535	929980	446
au334	SA0831	coenzyme A disulfide reductase	housekeeping (II)	TTGAATTGGGGTGAGTACCG	TTTTAAGCCCTGGTGCAAGT	942052	942453	402
au335	SA0837	hypothetical protein, similar to 2-isopropylmalate synthase	housekeeping (II)	AAGGGATGGTATGCAACAAAG	TCCTACAGTGTCCGCAAATG	950317	950794	478
au336	SA0917	phosphoribosylaminoimidazole carboxylase	housekeeping (II)	CCAAAGTTGTCCCGTTCATT	TGGTGCAATCTCGTTCACAT	1042431	1042882	452
au337	SA1045	carbamoyl-phosphate synthase small chain	housekeeping (II)	TCAAACGGACCAGGTAATCC	GGCATTGATATGACGCTCCT	1182692	1183135	444
au338	SA1046	carbamoyl-phosphate synthase large chain	housekeeping (II)	GAATCGGCCAAGGTGTAGAA	ATTGCGCGACCACCTAATAC	1184818	1185280	463
au339	SA1073	malonyl CoA-acyl carrier protein transacylase	housekeeping (II)	TGACGCATAGTTCGGCATTA	CTTAGCATCACGCCATTCAA	1214029	1214498	470
au340	SA1117	polyribonucleotide nucleotidyltransferase	housekeeping (II)	GTACGCTGCGGGTAAAATTC	TGTGGTCGACGATTTGTTGT	1266643	1267118	476
au341	SA1122	hypothetical protein, similar to precessing proteinase	housekeeping (II)	CGTGTTGATATTGCCGGTAG	GTAGCGCTTGTCACGATTGA	1275589	1276070	482
au342	SA1141	glycerol kinase	housekeeping (II)	GAAGGTGCACGAGAAAAAGC	GAACCCGAAACAAAGATGGA	1298079	1298566	488
au343	SA1142	aerobic glycerol-3-phosphate dehydrogenase	housekeeping (II)	ATTTCAGGTGGCGATGTAGG	GCGCTTCTTTAATTGCTTGC	1300525	1300930	406
au344	SA1165	threonine synthase	housekeeping (II)	ATATGCAGCGAGAGCAGGTT	CGCCTTATCCCAACTAGCAG	1327059	1327533	475
au345	SA1184	aconitate hydratase	housekeeping (II)	TGAAAAAGCGATTGCTGATG	CTGCTGGAGAGATGTGGTCA	1350857	1351356	500
au346	SA1197	prephenate dehydrogenase	housekeeping (II)	CACTCAATGCTTGCATACCG	AACTTGCAGCTGGTGGTTTT	1369809	1370243	435
au347	SA1198	hypothetical protein, similar to glucanase	housekeeping (II)	ATAACGCGATTGAGGGTGAA	ATTGATGCATTTGCACCGTA	1371316	1371718	403
au348	SA1230	hippurate hydrolase	housekeeping (II)	AACCCATTTGCTGATGAAGG	AAGAATGGCGTTGGTTTGTC	1404969	1405459	491
au349	SA1299	aroC, chorismate synthase	housekeeping (II)	GCATTGCTTGTGCGATACTG	CGCATGCAAATTGAGAAAGA	1506082	1506541	460
au350	SA1306	glycerol-3-phosphate dehydrogenase	housekeeping (II)	TTTTGATGATGCAGCAACTG	TGTTCTTGCAGAAAATGGACA	1511681	1512098	418
au351	SA1297	aroA, 3-phosphoshikimate 1-carboxyvinyltransferase	housekeeping (II)	TGGATATTACCGCCCATTTT	TTGGTAAAAGGCCAATGGAT	1503578	1504058	481
au352	SA1310	probable L-asparaginase	housekeeping (II)	ATGAAAAGGCCCGTCATAAA	AAAACCTTGTTGTGCGAGTT	1516729	1517186	458
au353	SA1342	phosphogluconate dehydrogenase	housekeeping (II)	CAAATGATGCTTTTGGACCAT	CAATTGCTGCTAAGGCAAAA	1551041	1551499	459
au354	SA1346	bmfBB, branched-chain alpha-keto acid dehydrogenase E2	housekeeping (II)	TGTAAATGTTCCACCTTGCAT	GCAATTGCGCAAAATATGGT	1555775	1556182	408
au355	SA1346	bmfBB, branched-chain alpha-keto acid dehydrogenase E2	housekeeping (II)	TGGTATTGTGCTGTTTTCACT	TTCCACGATATCAGGAACAA	1556201	1556621	421

au356	SA1347	bfmBAB, branched-chain alpha-keto acid dehydrogenase E1	housekeeping (II)	TAAACGCATGATTGGTGCAT	TTTGCTTCATCACCAGGTTT	1556915	1557382	468
au357	SA1348	bfmBAA, branched-chain alpha-keto acid dehydrogenase E1	housekeeping (II)	ATAATGCCAGCTGAAAGCAA	AAGAAAATTGCAACAGCAACTG	1557929	1558359	431
au358	SA1352	geranyltranstransferase homolog	housekeeping (II)	TTTATCTTCTGCGCCATCTTT	TCGACGAGGGAAATTAACAAA	1563060	1563555	496
au359	SA1354	hypothetical protein, similar to exodeoxyribonuclease large subunit	housekeeping (II)	TGCTTTTTCACCTTGAACCA	TTCAAAAAGCATTCAAGTGGTC	1564835	1565263	429
au360	SA1367	aminomethyltransferase	housekeeping (II)	AAAATGACGTTTGCTCCAAAT	CGATGCCTGTTCAATTTTCA	1576229	1576709	481
au361	SA1400	phosphate starvation-induced protein phoH homolog	housekeeping (II)	CCATTTTTGAGCCAAAACCTA	TAAGACGATTCGTGCGAAAA	1605000	1605451	452
au362	SA1427	5'-methylthioadenosine nucleosidase/S-adenosylhomocysteine nucleosidase	housekeeping (II)	TTGCAGTTGCTTCCATTTCA	AGCGAAATTTCAGTTGCACA	1630961	1631423	463
au363	SA1439	uridine kinase	housekeeping (II)	TCGGTTCAATAAATTGGTCA	ATCTGGCTCAGGAAAAACAA	1641785	1642272	488
au364	SA1491	glutamate-1-semialdehyde 2,1-aminomutase	housekeeping (II)	AAAATTAGCAATGCGCCGTA	AAAATAAATTGGCGCAGCTC	1699602	1700034	433
au365	SA1492	hemB, delta-aminolevulinic acid dehydratase	housekeeping (II)	TTCAGCAACAAAACCATCCA	ATTGAGATCATCAGCGACAA	1700815	1701313	499
au366	SA1493	uroporphyrinogen III synthase	housekeeping (II)	TGCATCGATTTGTTGATGTTC	CACAAACAAATGACATGCAAAG	1701528	1701985	458
au367	SA1510	glyceraldehyde 3-phosphate dehydrogenase 2	housekeeping (II)	AATGCCATGCCGTGTAATTT	TGATCGCAATCCTGAAAACTT	1719703	1720173	471
au368	SA1517	isocitrate dehydrogenase	housekeeping (II)	TTTCTGGGAATCGAATGTTTG	ATTGGACCGGATATTTGGAA	1730484	1730946	463
au369	SA1518	citrate synthase II	housekeeping (II)	TTACGATTCGTTTCGCCAAT	CTGAGATTGGGTCAATTGAAAA	1731107	1731509	403
au370	SA1521	6-phosphofructokinase	housekeeping (II)	TTTTGACAATCTTGCGCAGT	CGTAAAGTTGCAATCGAAAA	1736304	1736761	458
au371	SA1522	acetyl-CoA carboxylase carboxyl transferase subunit alpha	housekeeping (II)	TGTGCAACAAACGCTGATTT	AAATTTTGGTATGGCGCATC	1737361	1737849	489
au372	SA1523	acetyl-CoA carboxylase transferase beta subunit	housekeeping (II)	TGTTTGACGCATATCATTACGA	TATGAAATTTGGCGTTGCTG	1738259	1738715	457
au373	SA1533	ackA, acetate kinase homolog	housekeeping (II)	CCATTTGTGAACGTGCTTTA	TCCATCAAACAATGCCTGAA	1752185	1752634	450
au374	SA1537	hypothetical protein, similar to thiamine biosynthesis protein ThiI	housekeeping (II)	TTTATCAGCAACACGCATCA	TGCCGATAAAAATTTCCCAAT	1756048	1756534	487
au375	SA1538	hypothetical protein, similar to iron-sulfur cofactor synthesis protein nifZ	housekeeping (II)	ATTTTGCCGAATGCTTGAAC	GCAAAAGCCCAAATTAATGC	1757455	1757867	413
au376	SA1542	hypothetical protein, similar to glycerophosphoryl diester phosphodiesterase	housekeeping (II)	TCAACAAATTTTCTGGCCACT	TCATTTGACCAAAGACCAACA	1763385	1763867	483
au377	SA1548	hypothetical protein, similar to acylglycerol-3-phosphate O-acyltransfera homolog	housekeeping (II)	TTGTTTTTGCGCTTTACTGC	CGACAATTCGTGCGTTAAAA	1778549	1778970	422
au378	SA1548	hypothetical protein, similar to acylglycerol-3-phosphate O-acyltransfera homolog	housekeeping (II)	AAATCGCCATAATTTCAGACG	CCAAGAGAAAACAAAGGGAAA	1779340	1779810	471
au379	SA1566	endo-1,4-beta-glucanase homolog	housekeeping (II)	ACATTTGCGCCAACATACAA	AAAATCCAAATGCAAAACGTG	1797087	1797549	463
au380	SA1584	lysophospholipase homolog	housekeeping (II)	TTAATAGCGTTGGCATTGGTT	TGGTCAAGGTCAAACTTCAA	1823007	1823470	464
au381	SA1589	riboflavin specific deaminase	housekeeping (II)	CCAATTAATTTCGGGGCATA	CAAGCGAATGATAATGGACAAA	1827402	1827853	452
au382	SA1608	S-adenosylmethionine synthetase	housekeeping (II)	CGGTTGTTTCAAATCAAGCAT	ACATCAAAGCGCATGTCATT	1843323	1843768	446
au383	SA1614	menC, o-succinylbenzoic acid synthetase	housekeeping (II)	ACAACGATGTTGCCTTTTCA	AATTGAGCAATGGTTCGAAGA	1848623	1849098	476
au384	SA1615	menC, O-succinylbenzoic acid-CoA ligase	housekeeping (II)	TGCAATCAATTGTGCCTTTG	TTTAACAGCAACACCGGAAA	1849417	1849846	430
au385	SA1650	protoporphyrinogen oxidase	housekeeping (II)	TGTCCGAACCAATTCAAAAA	TGGTGCATTTTTCAGAGCAA	1884390	1884833	444
au386	SA1679	hypothetical protein, similar to D-3-phosphoglycerate	housekeeping (II)	TGAATCGTTTCTTGCGTTTC	TTACAACGTTGCCCGAATTT	1925498	1925949	452

		dehydrogenase						
au387	SA1694	conserved hypothetical protein	housekeeping (II)	ACGTGCTTTTCTTCGTTCAA	AAATCAGGACAGACCAATGAAA	1942222	1942687	466
au388	SA1729	nicotinate phosphoribosyltransferase homolog	housekeeping (II)	TTTTGCACCTTGTGCTTTCA	AAATTGATGCAGCATTGTGG	1980326	1980753	428
au389	SA1846	sucrose-6-phosphate hydrolase	housekeeping (II)	CATTATTTTGTGCGCCAATG	TGATCCCAACGGACTTATTTTT	2084448	2084872	425
au390	SA1862	2-isopropylmalate synthase	housekeeping (II)	TTGCCATGACGATTTAGGAA	TTTCTTTTTGTCCGCAATGG	2103107	2103605	499
au391	SA1913	UDP-GlcNAc 2-epimerase	housekeeping (II)	TTAAATGATGGCGCTTCTTC	ATGCTGCATCGCATTTGTTA	2162492	2162915	424
au392	SA1995	tagatose-6-phosphate kinase	housekeeping (II)	TTTCATCAAGTGGCTGGTTA	TGGTTTTATTGGCGGTGAAT	2267457	2267887	431
au393	SA2069	molybdenum cofactor biosynthesis protein C	housekeeping (II)	TGTCGCTCATTCAAGCATTA	ACCTGATTGGCGTTGAAAAT	2330886	2331291	406
au394	SA2078	hypothetical protein, similar to inosine-adenosine-guanosine-nucleoside hydrolase; IAG-nucleoside hydrolase	housekeeping (II)	CCCATCCAAACTAATTTTTCT	TGATGGTGGTGTAGATGATTT	2338344	2338778	435
au395	SA2084	urease alpha subunit	housekeeping (II)	ATATCGCATTCGCAGATTCA	ACCATTTGCATCGCCATTTA	2344580	2344983	404
au396	SA2129	hypothetical protein, similar to aldose 1-epimerase	housekeeping (II)	AATTGCGCATGTTGTGATGT	AAAACAACGGTGAACATCATTT	2394624	2395056	433
au397	SA2204	phosphoglycerate mutase, pgm homolog	housekeeping (II)	TTGAATTTCCGTGTGCAGAA	TTGCCATCGATGTAGCTTTT	2473703	2474116	414
au398	SA2248	hypothetical protein, similar to glutamate synthase (ferredoxin)	housekeeping (II)	TGCTTTTCTCCAACAATCAATG	TTGATTCGTTTCGTCGATCA	2524168	2524640	473
au399	SA2294	gluconokinase	housekeeping (II)	CATTTTCTTCAAACGGCGTA	TTGCAAAGCGTGTTAAACCA	2577068	2577505	438
au400	SA2420	alkaline phosphatase III precursor	housekeeping (II)	TGCTTGAACGTGCAAAAGAA	TTGGAATGCGCTGATTTATCT	2715420	2715885	466
au100	SA1142	glpD, aerobic glycerol-3-phosphate dehydrogenase	housekeeping (II)	AAAAACGATGTTTCCAGATCAA	TGAGATTGCTGATTGGTTCT	1317918	1318340	423
au101	SA1169	gamma-aminobutyrate permease	cell envelope (I)	TCATTGAATTTCACCCCTGA	AGGACCAATCATTGTCATCA	1330220	1330657	438
au102	SA1195	msrR, peptide methionine sulfoxide reductase regulator MsrR	information pathways (III)	ATGCCAAGAAACATGCGAAA	CCCTTTTTCAAAGATACACCAA	1367161	1367587	427
au103	SA1245	odhA, 2-oxoglutarate dehydrogenase E1	housekeeping (II)	CGTTTTTCAACCATTCTGCAA	TGGTGGATTTGAGCCAATTT	1419090	1419584	495
au105	SA1390	sigA, RNA polymerase sigma factor	information pathways (III)	GCTTTTGCTTCAATTTGTCG	ATGCAACATGGTGGATTAGA	1595439	1595931	493
au108	SA1488	valS, valine-tRNA ligase	information pathways (III)	ATGATACCAAGCCGGAATTT	TGAAATTGGTCAAAGACATCAA	1695320	1695728	409
au109	SA1531	ald, alanine dehydrogenase	housekeeping (II)	TCCCATACCACCATTAAGTTTT	TTCGCGTTAAGATGATTATGGA	1750526	1751025	500
au110	SA1537	hypothetical protein, similar to thiamine biosynthesis protein ThiI	housekeeping (II)	TTAATTGGTCCAACACGTTCA	ATTTGCGCAGCAATTTGAAG	1756157	1756585	429
au113	SA1815	hypothetical protein, similar to Na+-transporting ATP synthase	cell envelope (I)	TGGTTACATGGAATATTGACGA	TCGTGTTGTTACTGATTGGAAA	2055151	2055632	482
au115	SA1869	sigB, sigma factor B	information pathways (III)	TCATCTTGTTGCCCCATAAT	AAAGGACAATCACATCACGAA	2118355	2118767	413
au117	SA1945	hypothetical protein, similar to mannnose-6 phospate isomelase pmi	housekeeping (II)	TTTAATGAGGCGCTGATTTG	TGGTTAAATGGGAAATTTCTGG	2194352	2194754	403
au120	SA2123	hypothetical protein, similar to transcription regulator LysR family	information pathways (III)	CATCAAGATTACCGAAAAAGGA	TGCGCACTTGAGAATTTTTG	2388363	2388793	431
au124	SA2392	panB, 3-methyl-2-oxobutanoate hydroxymethyltransferase	housekeeping (II)	ACCAATCGGCATATCAACAA	TGTTATTGTTTTGGCGTGTCT	2680441	2680884	444
au131	SA0082	hypothetical protein	hypothetical (V)	TTCCATTTAGTTGCCCTTGT	ACCACCATCAAGATTTATAACG	91324	91796	473

au135	SA1220	hypothetical protein, similar to phosphate ABC transporter	cell envelope (I)	AAATCGCGAGAACGATTGAA	TTTGGTATCTGGGCATTGAT	1393204	1393648	445
au136	SA1577	hypothetical protein, similar to FmtB protein	other functions (IV)	TCGCATATGTTTGTTGAGCA	AAGCGAATATTCAAGCAACG	1809340	1809795	456
au137	SA1593	conserved hypothetical protein	hypothetical (V)	TGTATCCCAACCATGCTTTTT	CGATGCAGCAAAGAAACAAA	1832766	1833213	448
au23	none	non-coding	n. a.	TGTCGCAATGACAGTTGAAA	TGAAATCATACAAGTCGCTACA	1469725	1470183	459
au24	none	non-coding	n. a.	CGCAACCATAAATTTGCCTTT	GCACTTGTTTAACGGAATTCAA	1483427	1483925	499
au26	none	non-coding	n. a.	TGATGGATTCAATTGCTTGTTC	AAAAATCGCCGAAACACAAA	1670920	1671340	421
au27	none	non-coding	n. a.	CACCATTTGGGTTTGAAAGT	TTGATGCCGTAAATGTCGAA	1815418	1815916	499
au35	none	non-coding	n. a.	ACCTCTTGAGCCATATCATTT	ACTTCAAACCGCGCTAAATA	2175608	2176107	500
au36	none	non-coding	n. a.	AACTTGAAATGTTGCGTTGA	AAATGGCATGCGTTATGATTT	2348394	2348812	419
au37	none	non-coding	n. a.	TCAACACCTTCATTTGTAACGA	CATTATTTACCATGCGCCATT	2443855	2444349	495
au38	none	non-coding	n. a.	TCACTTCTTTTGATTGGACATT	TGAGTTGATGGGTTTTTCCA	2563607	2564032	426
au46	SA0232	hypothetical protein, similar to glutaryl-CoA dehydrogenase	housekeeping (II)	TGTAAATAATCACTGGCGAAG	TGTTGTTGGAGAATATGGTGT	281016	281523	508
au5	none	non-coding	n. a.	TGACAAACATTTGTTGGAAGC	TTTTTGTTTTGCTCGAAGTTCA	184251	184653	403
au55	SA0737	hypothetical protein	hypothetical (VI)	TGGATAAATGGCTACTGAAAAA	TCGCCTTTTAAAATTCGTTTG	844473	844887	415
au58	SA1188	parE, topoisomerase IV subunit B	information pathways (III)	TTTTAAAACAGCAATGACACG	TGGTAATATCGCTTGGAATTTG	1355191	1355686	496
au60	SA1394	glyS, glycyl-tRNA synthetase	information pathways (III)	TTGACGATGAAGGTATTGTTTG	CCCAACCAAATGGGAATTTA	1600764	1601240	477
au61	SA1397	cdd, cytidine deaminase	housekeeping (II)	CCAATTTCCTCATTGGCATT	ATGCAGATAAACCGTCATCA	1603292	1603754	463
au62	SA1409	DnaK protein	information pathways (III)	AAAATCATCACCGCCAAGTT	TGTTGTAGCTTTCAAAAATGGA	1614741	1615233	493
au64	SA1456	aspS, aspartyl-tRNA synthetase	information pathways (III)	TTCTGTTTCAAAGAAACGTCCA	AATCGTAAAATGCTTCCGTGA	1661432	1661922	491
au68	SA1859	acetolactate synthase large subunit	housekeeping (II)	TTGCCTTAATTGAAGATGGAGA	CTTGTTCGTGTCTTGCTAAAA	2099069	2099554	486
au81	SA0214	hexose phosphate transport protein uhpT	cell envelope (I)	TGGCCGATACTTAGGATTTT	GCAACTAATGCACCAATTTCAA	253735	254190	456
au82	SA0225	hypothetical protein, similar to glutaryl-CoA dehydrogenase	housekeeping (II)	CAATGCGAAGTGCGATTTTA	CTTTACAAGGATCGTCCAAA	271436	271889	454
au85	SA0442	probable DNA polymerase III, delta prime subunit	information pathways (III)	AAGCCAGTAGCTGAAATGATTA	TTTCCCGCTTTTTGAAACTG	515223	515695	473
au87	SA0594	teichoic acid translocation permease protei	cell envelope (I)	CACAAATGTTAATGCAAGCAA	CGATATGCGGTTTATCAATCA	687567	688039	473
au88	SA0610	hypothetical protein, similar to lipase LipA	hypothetical (VI)	TTCAAACGTAAAAATGCGGATA	TTATCACCCGCAATATAACCA	704489	704970	482
au89	SA0614	hypothetical protein, similar to two-component response regulator	information pathways (III)	GCGCAGATGATTATATGCAAAA	CAGTTCATGCGAGATTTCAA	708522	708961	440
au9	none	non-coding	n. a.	AACGCTTGAAAAGCGATGAA	TCATTAACCATCAACCGCATT	505090	505586	497
au91	SA0564	arginyl-tRNA synthetase argS	information pathways (III)	AAACAAATTGACATTGCTGGTC	TTCTTTCAAACGTGCTTCTTCA	657490	657948	459
au94	SA0781	hypothetical protein, similar to 2-nitropropan e dioxygenase	other functions (IV)	CGTCAAATTCTTTTGGCGTAA	AATGACCGGAATTGAAACGA	887890	888341	452
au95	SA0864	GTP pyrophosphokinase	cell envelope (I)	TCGCTGGTTTAAGAATGATG	TGCTTTAAGGCATTAGACTTG	978398	978888	491

au97	SA0935	ptsI, phosphoenolpyruvate-protein phosphatase	information pathways (III)	TGATATTCGCGACGTTTCTA	ACCTTGTGCACCATTTTCAA	1061317	1061806	490
au004 *	SA0084	hypothetical protein	hypothetical (VI)	AAGAAACAATGCCGTTTAATCA	TGGCAAGAAATGGAAATTGG	95053	95162	454
"	none	non-coding	n. a.			95163	95506	
au008 *	SA0353	ssb, single-strand DNA-binding protein	other functions (IV)	AAAATGCGCAACAAAATGGT	CATATGACGAGAACGTTTGAT	412508	412695	449
"	none	non-coding	n. a.			412696	412746	
"	SA0354	rpsR, 30S ribosomal protein S18	information pathways (III)			412747	412956	
au015 *	SA0702	llm, lipophilic protein	cell envelope (I)	TCGCATTTGCATCATAAACT	TGCTGGAAATTTCGATTTGA	801181	801435	488
"	none	non-coding	n. a.			801436	801599	
"	SA0703	hypothetical protein	hypothetical (V)			801600	801668	
au018 *	SA0993	uvrC, excinuclease ABC subunit C	information pathways (III)	TATTGCGTTCATTCGGTTCA	TTTTGATTGAGCCAAAAGGAGT	1126921	1127036	454
"	none	non-coding	n. a.			1127037	1127374	
au022 *	none	non-coding	n. a.	CGTAACGTGATATATGTTTCCA	AATTCATTCAGCAAGCACCA	1433703	1433964	414
"	SA1261	hypothetical protein	hypothetical (V)			1433965	1434116	
au025 *	SA1301	ndk, nucleoside diphosphate kinase	housekeeping (II)	ATAAAACGGTTTGCCTTGGT	TGAACGCAAAGAATTTGAAGAA	1507753	1507926	449
"	none	non-coding	n. a.			1507927	1508017	
"	SA1302	gercC, heptaprenyl diphosphate synthase component II	cell envelope (I)			1508018	1508201	
au033 *	SAS066	agrD, AgrD protein	information pathways (III)	CCCGCTGAATTAACGAATTT	TCGCTAATAATGAAACTGATGA	2080123	2080155	438
"	none	non-coding	n. a.			2080156	2080352	
"	SA1843	agrC, accessory gene regulator C	information pathways (III)			2080353	2080560	
au039 *	SA2408	cudT, choline transporter	cell envelope (I)	CGAACCAATTGAATCAGAAATG	AAATATTCTTAAATGCCGTCCT	2700197	2700346	411
"	none	non-coding	n. a.			2700347	2700607	
au043	SA0103	conserved hypothetical protein	hypothetical (V)	AAGCGATGAAGCCGTATTTTT	AATCATTGTCGGCATTTCGT	117750	118187	438
au047	SA0429	transcription activator of glutamate synthase operon	information pathways (III)	CAAATGGTGCCAAGCAATAA	TTTAAAAAGCAAGGACGCAAT	489886	490342	457
au050	SA0449	conserved hypothetical protein	hypothetical (V)	CCAGTTGACGCAATTGATTTT	TTCATTTCGCTTCCCTCTAT	521381	521848	468
au051	SA0501	RNA polymerase beta-prime chain	information pathways (III)	AAATCGGTCAACCAGTTCAA	TCACGCTTACCTTTAATTGC	586357	586786	430
au054	SA0689	ferrichrome ABC transporter permease	cell envelope (I)	TTTTAGTAGGTGCTGCCATT	ATTAATTGCTTCAGCACGAC	788137	788636	500
au057	SA1142	glpD, aerobic glycerol-3-phosphate dehydrogenase	housekeeping (II)	AAAAGGCGCAGAAATTATCAA	TGCCTTCTTCGTAAATTAATGG	1299840	1300339	500
au061	SA1397	cdd, cytidine deaminase	housekeeping (II)	CCAATTTCCTCATTGGCATT	ATGCAGATAAACCGTCATCA	1603292	1603754	463
au066	SA1614	menC	housekeeping (II)	TCTTGGCGATGATTCATTTTT	AAGTGCAAACGGCAATTGAT	1848056	1848544	489
au068 *	SA1859	ilvB, acetolactate synthase large subunit	housekeeping (II)	TTGCCTTAATTGAAGATGGAGA	CTTGTTCGTGTCTTGCTAAAA	2099069	2099280	486
"	none	non-coding	n. a.			2099281	2099308	
"	SA1858	ilvD, dihydroxy-acid dehydratase	housekeeping (II)			2099309	2099554	

au071 *	SA2167	scrA, sucrose-specific IIBC component	cell envelope (I)	AATGCGCCATTGCATCTAAA	TTCTTTCTTCTTCATTCGCACA	2435208	2435283	496
"	none	non-coding	n. a.			2435284	2435465	
"	SA2168	hypothetical protein	hypothetical (VI)			2435466	2435703	
au073 *	SA2214	bioA, aminotransferase	housekeeping (II)	GGGCAATTTTATTGAGTTGCTT	TCAGTGTGTTGCCATCAAAA	2485801	2486041	484
"	SA2215	bioD, dethiobiotin synthetase	housekeeping (II)			2486042	2486284	
au077	SA0022	probable 5'-nucleotidase precursor	housekeeping (II)	TGTCATCGGTATGGCTAAAT	GCCATGTTTCTTGTGTTGAA	31506	31999	494
au078	SA0140	phosphatase homologue	housekeeping (II)	TGCCATGTTAGAAGCGTTTT	AATAACATCTCGCATCGTCA	161073	161526	454
au080	SA0210	NADH-dependent dehydrogenase	cell envelope (I)	ATCAAAATCGCTTCAGACCA	TGAACATTTTGCCTCATCCA	249389	249807	419
au098	SA0956	Mn2+-transpor t protein	cell envelope (I)	TTGCGCCTTTTCTTCATTGT	TCTTTTGGATCATTGCAGAACT	1083110	1083534	425
au104 *	SA1342	gnd, phosphogluconate dehydrogenase	housekeeping (II)	TTAACGCTCCAATTTCACCA	CGACAATCGAAATGATTTAGTG	1551566	1551968	485
"	none	non-coding	n. a.			1551969	1552050	
au107	SA1460	relA, GTP pyrophosphokinase	housekeeping (II)	TTGCGTCTTTTGATTTTACCC	AAAAAGGCCGAATGATGGTT	1665882	1666352	471
au116	SA1923	transcription termination factor Rho	information pathways (III)	TTCTTCTGCGTTATGGTCATT	ATGCATTGTCATGCCTATCT	2171616	2172088	473
au132	SA0120	hypothetical protein	hypothetical (V)	TTCCAGTGCAAGAAATCCAT	TTCCAATTACGCAGTTGTTCA	139445	139919	475
au133	SA0321	carbohydrate kinase, PfkB family	information pathways (III)	TGCCTTTAAATGTGCATTGG	TCAATCAACAATCTCGTCAAAA	377922	378349	428

* some PCR-amplified fragments span borders of open reading frames as indicated

Gene categories: I, cell envelope; II, housekeeping; III, information pathways; IV, other functions; V, conserved hypothetical; VI, hypothetical
