# Supplementary material for: A Timescale for Evolution, Population Expansion, and Spatial Spread of an Emerging Clone of Methicillin-Resistant Staphylococcus aureus
Source: PLoS Pathog. 2010 Apr 8;6(4):e1000855. doi: 10.1371/journal.ppat.1000855 (PMC2851736; doi:10.1371/journal.ppat.1000855)
Supplement: Table S4 — Polymorphisms newly discovered in 269 genome fragments from 73 S. aureus isolates (0.01 MB PDF) [file ppat.1000855.s006.pdf]

## Table S4a. Polymorphisms newly discovered in 269 genome fragments from 73 *S. aureus* isolates

| Polymorphism | Quality | Ancestral | Derived | Position in N315 genome | ORF |
|---|---|---|---|---|---|
| ST225_au207-1 | non-synonymous | G | A | 263406 | SA0220 |
| ST225_au207-2 | non-synonymous | A | G | 263113 | SA0220 |
| ST225_au210-1 | synonymous | A | G | 369667 | SA0311 |
| ST225_au220-1 | non-synonymous | G | A | 631889 | SA0537 |
| ST225_au238-1 | non-synonymous | G | A | 2263605 | SA1991 |
| ST225_au247-1 | non-synonymous | A | G | 419087 | SA0361 |
| ST225_au248-1 | non-synonymous | A | T | 622391 | SA0527 |
| ST225_au261-1 | synonymous | C | T | 1824296 | SA1585 |
| ST225_au269-1 | non-synonymous | G | A | 2517195 | SA2240 |
| ST225_au291-1 | non-synonymous | G | T | 1001705 | SA0881 |
| ST225_au297-1 | non-synonymous | A | T | 1504686 | SA1298 |
| ST225_au312-1 | synonymous | G | A | 208745 | SA0178 |
| ST225_au313-1 | non-synonymous | C | A | 271206 | SA0225 |
| ST225_au319-1 | non-synonymous | A | G | 402572 | SA0344 |
| ST225_au320-1 | synonymous | G | A | 407135 | SA0347 |
| ST225_au323-1 | non-synonymous | G | A | 480034 | SA0418 |
| ST225_au326-1 | non-synonymous | A | G | 627571 | SA0533 |
| ST225_au329-1 | synonymous | T | C | 753556 | SA0658 |
| ST225_au345-1 | non-synonymous | T | C | 1351084 | SA1184 |
| ST225_au345-2 | non-synonymous | A | G | 1351210 | SA1184 |
| ST225_au346-1 | non-synonymous | C | T | 1370037 | SA1197 |
| ST225_au349-1 | non-synonymous | A | T | 1506401 | SA1299 |
| ST225_au352-1 | synonymous | T | C | 1517048 | SA1310 |
| ST225_au354-1 | synonymous | C | T | 1555901 | SA1346 |
| ST225_au359-1 | non-synonymous | A | G | 1564858 | SA1354 |
| ST225_au360-1 | synonymous | A | G | 1576528 | SA1367 |
| ST225_au362-1 | non-synonymous | C | T | 1631030 | SA1427 |
| ST225_au367-1 | non-synonymous | G | A | 1719763 | SA1510 |
| ST225_au367-2 | non-synonymous | T | C | 1719985 | SA1510 |
| ST225_au378-1 | non-synonymous | G | A | 1779511 | SA1548 |
| ST225_au385-1 | synonymous | C | T | 1884695 | SA1650 |

| | | | | | |
|---|---|---|---|---|---|
| ST225_au013-1 | intergenic | C | T | 605040 | none |
| ST225_au018-1 | intergenic | C | G | 1127102 | none |
| ST225_au018-2 | intergenic | C | T | 1127250 | none |
| ST225_au018-3 | intergenic | T | G | 1127326 | none |
| ST225_au023-1 | intergenic | T | C | 1470071 | none |
| ST225_au025-1 | non-synonymous | G | A | 1508046 | SA1302 |
| ST225_au038-1 | intergenic | T | C | 2563753 | none |
| ST225_au046-1 | intergenic | C | T | 274971 | none |
| ST225_au050-1 | synonymous | T | C | 521443 | SA0449 |
| ST225_au080-1 | non-synonymous | T | C | 249620 | SA0210 |
| ST225_au085-1 | non-synonymous | C | A | 515464 | SA0442 |
| ST225_au097-1 | non-synonymous | C | T | 1061339 | SA0935 |
| ST225_au131-1 | intergenic | G | T | 91487 | none |
| ST225_au131-2 | intergenic | C | A | 91452 | none |
| ST225_au131-3 | intergenic | A | G | 91465 | none |
| ST225_au133-1 | synonymous | T | A | 378156 | SA0321 |
| ST225_au135-1 | synonymous | A | G | 1393583 | SA1220 |

**Indels.**

| | | | | | |
|---|---|---|---|---|---|
| ST225_au102-1 | insertion (1 nt) | - | A | 1367331 | SA1195 |

## Table S4b. Sequence differences between ST225 and N315 (on branch ST5-K)

| Polymorphism | Quality | Ancestral | Derived | Position in N315 genome | ORF | |
|---|---|---|---|---|---|---|
| ST225_au210-1 | synonymous | G | A | 369667 | SA0311 | |
| ST225_au004-1 | intergenic | G | C | 95177 | none | |
| ST225_au008-1 | intergenic | G | A | 412719 | none | |
| ST225_au018-4 | intergenic | T | G | 1127179 | none | |
| ST225_au022-1 | intergenic | G | A | 1433941 | none | |
| ST225_au033-1 | intergenic | T | A | 2080266 | none | |
| ST225_au039-1 | intergenic | T | A | 2700440 | none | |
| ST225_au050-2 | synonymous | A | G | 521614 | SA0449 | |
| ST225_au068-1 | synonymous | T | C | 2099406 | SA1859 | |
| ST225_au116-1 | non-synonymous | T | G | 2171885 | SA1923 | |
| au228-1 | non-synonymous | T | C | 1264963 | SA1115 | ascertained previously (PNAS 105: 14130) |
