# Supplementary material for: A Timescale for Evolution, Population Expansion, and Spatial Spread of an Emerging Clone of Methicillin-Resistant Staphylococcus aureus
Source: PLoS Pathog. 2010 Apr 8;6(4):e1000855. doi: 10.1371/journal.ppat.1000855 (PMC2851736; doi:10.1371/journal.ppat.1000855)
Supplement: Table S6 — Additional PCR primers. (0.08 MB DOC) [file ppat.1000855.s008.doc]

| **Table S6. Additional PCR primers.** | | |  | |  | |  | |  | |  | |  | |  | |  | |  | |  | |
| --- | --- | --- | --- | --- | --- | --- | --- | --- | --- | --- | --- | --- | --- | --- | --- | --- | --- | --- | --- | --- | --- | --- |
| **Primer name** | **Primer sequence** | **Position in N315 genome** | | **Product length** | | **Target** | | **Reference** | |  | |  | |  | |  | |  | |  | |  |
| norG-L | aggttggattgttgcttctga | 119362 | |  | | norG | | this study | |  | |  | |  | |  | |  | |  | |  |
| norG-R-wt | GCGGGCtagtcaatttgcattcttacatc | 119405 | | 73 | | norG wildtype | | this study | |  | |  | |  | |  | |  | |  | |  |
| norG-R-Del | GCGGGCAGGGCGGCGGGGGCGGGGCCtagtcaatttgcattcttatgcc | 119401 | | 89 | | norG deletion | | this study | |  | |  | |  | |  | |  | |  | |  |
| dru3-L | atgtccctctgcatcaatgg | 42162 | | 823 | | dru deletion | | this study | |  | |  | |  | |  | |  | |  | |  |
| dru3/4-R | atttgccaactttcgatga | 43982 | |  | |  | | this study | |  | |  | |  | |  | |  | |  | |  |
| xsau325 | GGATCAAACGGCCTGCACA | 34016 | | 278 | | orfX | | J. Clin. Microbiol. 42: 1875 | |  | |  | |  | |  | |  | |  | |  |
| mecii574 | GTCAAAAATCATGAACCTCATTACTTATG | 34293 | |  | | SCCmec remnant | | J. Clin. Microbiol. 42: 1875 | |  | |  | |  | |  | |  | |  | |  |
| ccrB-F | TATCGTAAAATAGCSAATGCAYTVAATCACAAAGG | 64434 | | 513 | | ccrB (SCCmec recombinase) | | Clin. Microb. Inf. 12: 1175 | |  | |  | |  | |  | |  | |  | |  |
| ccrB-R | ACTTTATCACTTTTGACAATTTCRAGTATTTG | 63920 | |  | |  | | Clin. Microb. Inf. 12: 1175 | |  | |  | |  | |  | |  | |  | |  |
| cch-F | MAATCGTGAASAWGAAGTYATTMAATGGTT | 67325 | | 371 | | cch (SCCmec helicase) | | Clin. Microb. Inf. 12: 1175 | |  | |  | |  | |  | |  | |  | |  |
| cch-R | GCAATSATTTTBACYTSGATATGRTYATCTT | 66955 | |  | |  | | Clin. Microb. Inf. 12: 1175 | |  | |  | |  | |  | |  | |  | |  |
| **Primer name** | **Primer sequence** | **Position in 04-02981 genome** | | **Product length** | | **Target** | | **Reference** | |  | |  | |  | |  | |  | |  | |  |
| phage1-L | tgggattcatcgaaccattt | 876030 | | 249 | | non-coding region upstream of prophage | | this study | |  | |  | |  | |  | |  | |  | |  |
| phage1-R | tgctcaaatcactgaaacga | 876279 | |  | | phage integrase | | this study | |  | |  | |  | |  | |  | |  | |  |
| phage2-L | aaactgctttcgcagattta | 879859 | | 261 | | phage transcriptional regulator gene | | this study | |  | |  | |  | |  | |  | |  | |  |
| phage2-R | aaaaattgccgatcacttcg | 880119 | |  | | phage transcriptional regulator gene | | this study | |  | |  | |  | |  | |  | |  | |  |
| phage3-L | aaacaagaagtcgaccacaaaa | 892834 | | 227 | | gene for phage terminase, small subunit | | this study | |  | |  | |  | |  | |  | |  | |  |
| phage3-R | atttgcccaatcatatttca | 893060 | |  | | gene for phage terminase, large subunit | | this study | |  | |  | |  | |  | |  | |  | |  |
| phage4-L | aaaagctggcattcagtcaa | 916738 | | 245 | | holin gene | | this study | |  | |  | |  | |  | |  | |  | |  |
| phage4-R | agtgaaatcattccatgttgg | 916982 | |  | | gene encoding CHAP domain protein | | this study | |  | |  | |  | |  | |  | |  | |  |
| phage5-L | aaatgatcaaaccacaccac | 919996 | | 261 | | non-coding region, 3' end of prophage | | this study | |  | |  | |  | |  | |  | |  | |  |
| phage5-R | atttccggaagtcaagaatg | 920257 | |  | | non-coding region downstream of prophage | | this study | |  | |  | |  | |  | |  | |  | |  |
