# Supplementary material for: A Timescale for Evolution, Population Expansion, and Spatial Spread of an Emerging Clone of Methicillin-Resistant Staphylococcus aureus
Source: PLoS Pathog. 2010 Apr 8;6(4):e1000855. doi: 10.1371/journal.ppat.1000855 (PMC2851736; doi:10.1371/journal.ppat.1000855)
Supplement: Table S7 — (a) Oligonucleotide primers for genome sequence gap closure. (b) Sequencing primers for genome sequence gap closure. (0.20 MB DOC) [file ppat.1000855.s009.doc]

| **Table S7a. PCR primers for genome sequence gap closure.** | | | | | | |  |  | | | |  | |  | |  | |  |  |
| --- | --- | --- | --- | --- | --- | --- | --- | --- | --- | --- | --- | --- | --- | --- | --- | --- | --- | --- | --- |
| **gap** | **5' end of gap** | | **3' end of gap** | **left primer** | | **right primer** | | |  |  |  | |  | |  | |  | | |
| gap2 | 54569 | | 61288 | TGCCCATCATTTCACTAATCTCTT | | GACCGAGAGCATCTCATTCTCATA | | |  |  |  | |  | |  | |  | | |
| gap3 | 89809 | | 96115 | TCTTATGCCACGCACATAGATTTT | | CGAAATTGCACCAAATACATTGTT | | |  |  |  | |  | |  | |  | | |
| gap4 | 153503 | | 153736 | acatcggcgcattaaaagat | | caaggtttggtaaagtttgatcg | | |  |  |  | |  | |  | |  | | |
| gap5 | 188288 | | 188473 | AAAATATGGCATTGCACATTCAAC | | CGCATTTTCGTTCAGTCAACTACT | | |  |  |  | |  | |  | |  | | |
| gap6 | 452159 | | 452163 | CAAAGCAGACTTTGCCTTTATTCA | | AATAATACGTTCGACTTGGGCATC | | |  |  |  | |  | |  | |  | | |
| gap7 | 506053 | | 511809 | tgaggcaaacaaaacaactca | | gctgttcactttttataatacttctg | | |  |  |  | |  | |  | |  | | |
| gap8 | 550484 | | 555663 | AATAACGGGAAGTAGCTCAGCTTG | | ACTAAACTCGTTGCGCTCTTTTCT | | |  |  |  | |  | |  | |  | | |
| gap10 | 651334 | | 652174 | GTCGCGTAAAAGCTTTGGAA | | TCGGCAAATAGTCATCGTCA | | |  |  |  | |  | |  | |  | | |
| gap11 | 678225 | | 679727 | tcataagctttgcctaccgaata | | tgtgaatgcacttatcttcatcttt | | |  |  |  | |  | |  | |  | | |
| gap12 | 794600 | | 794610 | TTTGTAAATGGTGAAATGGTTTGG | | TTCTTATTGGATTCGTCGTTTTCC | | |  |  |  | |  | |  | |  | | |
| gap13 | 830900 | | 831180 | TTATTTTGAGTGAGGTGGGACAGA | | TAGTAGTTGACCGAACGAAAATGC | | |  |  |  | |  | |  | |  | | |
| gap14 | 874000 | | 874400 | AGATCAATCTAGGGAGTGGGACAG | | CAAACTCAATGTTTGACTAGCTGGA | | |  |  |  | |  | |  | |  | | |
| gap15 | 893780 | | 895300 | TGAAGATTAAGCCAACCATTCAAG | | CGAGCATAGTCGATATGATTTGATG | | |  |  |  | |  | |  | |  | | |
| gap16 | 1138950 | | 1140490 | AAGTCAGGAAAGCAAATCAAGTACA | | CTACCAATTGCATTGGACTTCAAC | | |  |  |  | |  | |  | |  | | |
| gap17 | 1526080 | | 1530502 | ATTCATTTTGATACCCGACGTTTT | | CATTAATTTTAGGGGGATGTTCCA | | |  |  |  | |  | |  | |  | | |
| gap18 | 1618570 | | 1618650 | AGTGGCTCTTACACATTAGCCAGA | | ACGTCCTGGGCTTTACAATGTTAT | | |  |  |  | |  | |  | |  | | |
| gap19 | 1684400 | | 1691150 | TCATCAAAGTAACCCGCTTCTACA | | TAGTATTGCGGTGAGAGAAAATGC | | |  |  |  | |  | |  | |  | | |
| gap20 | 1859530 | | 1859545 | ATCTCTTGTAATGTCGCGCTGTAG | | CAGTCGTACTCCCACATGGTGTAT | | |  |  |  | |  | |  | |  | | |
| gap21 | 1914670 | | 1923986 | GCTTAACTTCCAATATTGAACTCATCA | | TTTGACTATTGAAATTCGAACAAATACA | | |  |  |  | |  | |  | |  | | |
| gap22 | 2108520 | | 2114280 | ACATTTCTCGTTTCGTCAGATTCA | | GGGTGTAGTTTAATGGCAAAACCT | | |  |  |  | |  | |  | |  | | |
| gap23 | 2134330 | | 2135860 | CCGCATACAGATCTCAATCCAAT | | ACCCAAACTAAAGGAGACAAGTGC | | |  |  |  | |  | |  | |  | | |
| gap24 | 2188920 | | 2189157 | TCATCATGGCATTTATGATGTCTT | | ACAACAAGACATTGAAGCAGTGGT | | |  |  |  | |  | |  | |  | | |
| gap25 | 2230160 | | 2235979 | TTTTCTTTGTCCAAATGTTGATGG | | TAAAGTCGTCAAAAACGACGCAGT | | |  |  |  | |  | |  | |  | | |
| gap26 | 2315570 | | 2315780 | TGGCTCTAAAAACAAAACGCAATA | | ACGTGTATCAGGCAGTGTTTGTCT | | |  |  |  | |  | |  | |  | | |
| gap27 | 2560420 | | 2573180 | TTAATCACATCATCCACTGGCTCT | | TAGATGGTGAGGGTGGTTATGTTG | | |  |  |  | |  | |  | |  | | |
|  | |  | | |  | |  |  | | | |  | |  | |  | |  |  |
| **Table S7b. Sequencing primers for genome sequence gap closure** | | | | |  | |  |  | | | |  | |  | |  | |  |  |
| gap19-575-F | | TCTGAAGCTAAATGCTCAAGCCATGA | | |  | |  |  | | | |  | |  | |  | |  |  |
| gap19-1049-F | | AAGAATGAATAATCATTCAAAAGCTC | | |  | |  |  | | | |  | |  | |  | |  |  |
| gap19-1549-F | | TAATAAAGTTCGTTATCAACCAAATC | | |  | |  |  | | | |  | |  | |  | |  |  |
| gap19-2046-F | | GAGAAGGGATTTGCGAAAAGATTGC | | |  | |  |  | | | |  | |  | |  | |  |  |
| gap19-2550-F | | TTCATATACTTAACGAGTGCTTTCAC | | |  | |  |  | | | |  | |  | |  | |  |  |
| gap19-3052-F | | GTCTAACAGAATCCGTATTTCCAATC | | |  | |  |  | | | |  | |  | |  | |  |  |
| gap19-3551-F | | TTTAATAAGGATTTCCTCCGAACGAG | | |  | |  |  | | | |  | |  | |  | |  |  |
| gap19-4060-F | | CAATAACCATTTGGAAGCACTTGTGC | | |  | |  |  | | | |  | |  | |  | |  |  |
| gap19-4566-F | | TGATAATACTTTAAAAAGAAATCTCC | | |  | |  |  | | | |  | |  | |  | |  |  |
| gap19-5066-F | | TACAAGTTGTTTAAAATATGTATTCC | | |  | |  |  | | | |  | |  | |  | |  |  |
| gap19-5576-F | | GGATTCTTACTTAATTCTATGGCAGA | | |  | |  |  | | | |  | |  | |  | |  |  |
| gap19-6076-F | | CATAAGTTCTTTACTCACATGTATTG | | |  | |  |  | | | |  | |  | |  | |  |  |
| gap27-781-F | | TTTCTACAATCGAGTCTCCTTTTACA | | |  | |  |  | | | |  | |  | |  | |  |  |
| gap27-1280-F | | CTTTCTCTCCTGTTGGTAACTTCGGA | | |  | |  |  | | | |  | |  | |  | |  |  |
| gap27-1759-F | | TTTGTGATCTCTTCTTTCGATTCACC | | |  | |  |  | | | |  | |  | |  | |  |  |
| gap27-2381-F | | CAAGTCAGTTCTCATCCAGCCATTTG | | |  | |  |  | | | |  | |  | |  | |  |  |
| gap27-2879-F | | CCAACCATCAGCACCTGTGGTATTTG | | |  | |  |  | | | |  | |  | |  | |  |  |
| gap27-3380-F | | TACTTCTTCAATCTTCACTTCTTTTG | | |  | |  |  | | | |  | |  | |  | |  |  |
| gap27-3878-F | | GCACCAAGATATTAAAATCTCGAAAC | | |  | |  |  | | | |  | |  | |  | |  |  |
| gap27-4381-F | | CACTATATTTTGATTTTGTTCAATTG | | |  | |  |  | | | |  | |  | |  | |  |  |
| gap27-4879-F | | CAATACAACGTAAAAAGATTGCTTGT | | |  | |  |  | | | |  | |  | |  | |  |  |
| gap27-5382-F | | TCTCAATTTAGTATAAATAAAAATAC | | |  | |  |  | | | |  | |  | |  | |  |  |
| gap27-5880-F | | GTTAATGGATCAATAATTCCATAACG | | |  | |  |  | | | |  | |  | |  | |  |  |
| gap27-6365-F | | CCCTAAACCAGCAGCGGGAATGATTG | | |  | |  |  | | | |  | |  | |  | |  |  |
| gap27-6864-F | | AATAAATTTAGGCCAATTACATAACC | | |  | |  |  | | | |  | |  | |  | |  |  |
| gap27-7357-F | | AAAATGAAACTCATTGATCCAGTTAC | | |  | |  |  | | | |  | |  | |  | |  |  |
| gap27-7837-F | | TCTTGGTTTATATATTTTATATTTTC | | |  | |  |  | | | |  | |  | |  | |  |  |
| gap27-8330-F | | GGTTTTTTAGGTTCTTCTTTAGCAGG | | |  | |  |  | | | |  | |  | |  | |  |  |
| gap27-8831-F | | AAATCGATATCTACGATATTACCACC | | |  | |  |  | | | |  | |  | |  | |  |  |
| gap27-9335-F | | GAATCTTCTTCAGTTTCAATAATACC | | |  | |  |  | | | |  | |  | |  | |  |  |
| gap27-9829-F | | ATAAACACTTTCAGCTAATTCATCTG | | |  | |  |  | | | |  | |  | |  | |  |  |
| gap27-10337-F | | AATGTGAAATCAAAATAATCTCCTGC | | |  | |  |  | | | |  | |  | |  | |  |  |
| gap27-10825-F | | TGCTTTACTTTCAGTAGCTGAACTCC | | |  | |  |  | | | |  | |  | |  | |  |  |
| gap27-11306-F | | ACAAGTCACTGAAATGCCTTTCATTG | | |  | |  |  | | | |  | |  | |  | |  |  |
| gap27-11786-F | | GTAGTTCAGATTTCTTAGATTGTGCT | | |  | |  |  | | | |  | |  | |  | |  |  |
| gap22-42-F | | TCTAATTGAAATCATCTTATGACTGC | | |  | |  |  | | | |  | |  | |  | |  |  |
| gap22-363-F | | TCATTTAGCTCTACTAAACTCGTTGC | | |  | |  |  | | | |  | |  | |  | |  |  |
| gap22-741-F | | GTTTGCTTTTATTTTGACGTTTTAGAC | | |  | |  |  | | | |  | |  | |  | |  |  |
| gap22/25-1071-F | | CATCATCTTTGAGGGATCTTATAACC | | |  | |  |  | | | |  | |  | |  | |  |  |
| gap22/25-1547-F | | GCAGTCAAGCTCCCTTATGCCTTTAC | | |  | |  |  | | | |  | |  | |  | |  |  |
| gap22/25-2028-F | | AAGCTAACCACTCCTCTTAACCTTCC | | |  | |  |  | | | |  | |  | |  | |  |  |
| gap22/25-2515-F | | ACGCCTGTCGGCCTCAGCTTAGGACC | | |  | |  |  | | | |  | |  | |  | |  |  |
| gap22/25-3007-F | | CAAACAGTGCTCTACCTCCAATAATC | | |  | |  |  | | | |  | |  | |  | |  |  |
| gap22/25-3482-F | | ACGGAATTTCACGTGCTCCGTCGTAC | | |  | |  |  | | | |  | |  | |  | |  |  |
| gap22/25-3945-F | | AAGTCAAACGTTAACATGAAGTTACG | | |  | |  |  | | | |  | |  | |  | |  |  |
| gap22/25-4431-F | | TACCTTGTTACGACTTCACCCCAATC | | |  | |  |  | | | |  | |  | |  | |  |  |
| gap22/25-4912-F | | GGGAAGGCTCTATCTCTAGAGTTGTC | | |  | |  |  | | | |  | |  | |  | |  |  |
| gap22/25-5298-F | | TTTCCAATGACCCTCCACGGTTGAGC | | |  | |  |  | | | |  | |  | |  | |  |  |
| gap25-43-F | | ATTGCTGTTGGTCCTAAATCAACACC | | |  | |  |  | | | |  | |  | |  | |  |  |
| gap25-375-F | | AGCTGGATTCGAACCAACGAGTGACG | | |  | |  |  | | | |  | |  | |  | |  |  |
| gap25-763-F | | ATCGGTTAACAGCCGATAGCTCTACC | | |  | |  |  | | | |  | |  | |  | |  |  |
| gap17-10-F | | AATATAAAAATATTCATTTTGATACC | | |  | |  |  | | | |  | |  | |  | |  |  |
| gap17-490-F | | TGATTTTGTTTCATTGTTGGATTCGT | | |  | |  |  | | | |  | |  | |  | |  |  |
| gap17-1099-F | | TTTTTGCGCTCGATATTCAAAGCCAC | | |  | |  |  | | | |  | |  | |  | |  |  |
| gap17-1557-F | | ATTTATGTGTTTGTAAAACTTTTATG | | |  | |  |  | | | |  | |  | |  | |  |  |
| gap17-1995-F | | ACAAGTTACATTAGGAATCAGGGACA | | |  | |  |  | | | |  | |  | |  | |  |  |
| gap17-2500-F | | ATAAAAGATTTTATACCTTTAAACAAC | | |  | |  |  | | | |  | |  | |  | |  |  |
| gap17-3000-F | | GATTAGTGGTACAGTATCGTATGTGT | | |  | |  |  | | | |  | |  | |  | |  |  |
| gap17-3499-F | | TTTTGTAACCAATCATCTCCGATATC | | |  | |  |  | | | |  | |  | |  | |  |  |
| gap17-4003-F | | ATTACTATTAGCAGTATAATCTAACT | | |  | |  |  | | | |  | |  | |  | |  |  |
