# Supplementary material for: A Timescale for Evolution, Population Expansion, and Spatial Spread of an Emerging Clone of Methicillin-Resistant Staphylococcus aureus
Source: PLoS Pathog. 2010 Apr 8;6(4):e1000855. doi: 10.1371/journal.ppat.1000855 (PMC2851736; doi:10.1371/journal.ppat.1000855)
Supplement: Table S8 — Genomic differences between 04-02981, JH1/JH9, and N315. (a) Derived traits in the genome of 04-02981. (b) Derived traits in the JH strain. (c) Derived traits shared by 04-02981 and the JH strain. (0.09 MB PDF) [file ppat.1000855.s010.pdf]

Table S8a. Derived traits in the genome of 04-02981.

Polymorphism	Quality	Ancestral	Derived	Position in N315 genome	ORF	Product
**Substitutions.**						
225-1	non-synonymous	c	t	4570	SA0004	DNA repair and genetic recombination protein
225-2	non-synonymous	g	a	15421	SA0010	SA0010~hypothetical protein, similar to amino acid permease
225-3	non-synonymous	g	a	32836	SA0022	SA0022~hypothetical protein, similar to 5-nucleotidase
225-4	intergenic	c	a	33521	intergenic	
225-5	non-synonymous	c	t	37603	SA0028	truncated replication protein for pUB110 plasmid
225-6	non-synonymous	g	a	37636	SA0028	truncated replication protein for pUB110 plasmid
225-7	synonymous	c	a	41289	SA0033	kanamycin nucleotidyltransferase
225-8	intergenic	c	t	41688	intergenic	
225-9	non-synonymous	a	g	78271	SA0068	SA0068~potassium-transporting ATPase A chain homologue
225-10	non-synonymous	t	c	84064	SA0074	hypothetical protein
225-11	non-synonymous	a	c	105109	SA0093	hypothetical protein
225-12	non-synonymous	t	c	123016	SA0107	Immunoglobulin G binding protein A precursor
225-13	non-synonymous	t	c	123017	SA0107	Immunoglobulin G binding protein A precursor
225-14	non-synonymous	c	t	139345	SA0120	hypothetical protein
225-15	non-synonymous	a	g	190067	SA0166	SA0166~hypothetical protein, similar to nitrate transporter
225-16	intergenic	g	t	214755	intergenic	
225-17	synonymous	c	t	242164	SA0203	hypothetical protein
225-18	intergenic	c	a	325638	intergenic	
225-19	intergenic	c	t	349905	intergenic	
225-20	synonymous	a	g	369667	SA0311	SA0311~hypothetical protein, similar to trimethylamine dehydrogenase (EC
225-21	synonymous	t	a	377708	SA0320	SA0320~hypothetical protein, similar to PTS fructose-specific enzyme
225-22	non-synonymous	g	a	391775	SA0333	conserved hypothetical protein
225-23	intergenic	a	g	401139	intergenic	
225-24	synonymous	g	a	447392	SA0387	exotoxin 11
225-25	synonymous	t	c	455093	SA0394	hypothetical protein
225-26	synonymous	c	t	456114	SA0394	hypothetical protein

225-27	non-synonymous	t	c	561467	SA0481	conserved hypothetical protein
225-28	synonymous	c	t	579874	SA0500	RNA polymerase beta chain
225-29	intergenic	a	t	612692	intergenic	
225-30	synonymous	a	g	655592	SA0562	alcohol dehydrogenase I
225-31	intergenic	a	g	695890	intergenic	
225-32	non-synonymous	c	t	740824	SA0646	SA0646~hypothetical protein, similar to deoxyribodipyrimidine photolyase
225-33	intergenic	t	c	743320	intergenic	
225-34	synonymous	t	c	835610	SA0729	triosephosphate isomerase
225-35 [2]	intergenic	g	a	885664	intergenic	
225-36 [2]	intergenic	g	a	885671	intergenic	
225-37	non-synonymous	c	t	918988	SA0815	SA0815~peptidyl-prolyl cis-trans isomerase homologue
225-38	non-synonymous	c	t	1033916	SA0909	Fmt, autolysis and methicillin resistant-related protein
225-39	intergenic	t	c	1083917	intergenic	
225-40	non-synonymous	t	a	1089945	SA0962	conserved hypothetical protein
225-41	synonymous	a	t	1121192	SA0990	DNA-dependent DNA polymerase beta chain
225-42	intergenic	a	t	1138582	intergenic	
225-43	non-synonymous	c	t	1153216	SA1018	conserved hypothetical protein
225-44	non-synonymous	c	t	1236878	SA1092	SA1092~hypothetical protein, similar to DNA processing Smf
225-45	intergenic	g	a	1285045	intergenic	
225-46	non-synonymous	g	t	1304352	SA1147	SA1147~hypothetical protein, similar to GTP-binding protein proteinase
225-47	intergenic	t	a	1315671	intergenic	
225-48	non-synonymous	c	g	1321547	SA1160	thermonuclease
225-49	non-synonymous	t	c	1344552	SA1181	SA1181~hypothetical protein, similar to exonuclease SbcC
225-50	non-synonymous	c	t	1356086	SA1188	topoisomerase IV subunit B
225-51	non-synonymous	g	a	1366449	SA1194	SA1194~peptide methionine sulfoxide reductase homolog
225-52	non-synonymous	t	c	1380774	SA1207	FemB protein
225-53	non-synonymous	c	t	1430837	SA1257	peptide methionine sulfoxide reductase
225-54	non-synonymous	g	a	1452458	SA1267	SA1267~hypothetical protein, similar to streptococcal adhesin emb
225-55	non-synonymous	g	c	1467113	SA1268	SA1268~hypothetical protein, similar to streptococcal adhesin emb

225-56	intergenic	c	a	1475485	intergenic	
225-57	intergenic	t	c	1491089	intergenic	
225-58	non-synonymous	c	t	1505445	SA1298	3-dehydroquinate synthase
225-59	synonymous	a	g	1524257	SA1317	hypothetical protein
225-60	non-synonymous	c	t	1569865	SA1360	Xaa-Pro dipeptidase
225-61	synonymous	c	t	1577292	SA1368	SA1368~hypothetical protein, similar to shikimate kinase (SK)
225-62	synonymous	t	a	1597008	SA1391	DNA primase
225-63	non-synonymous	g	a	1627451	SA1421	conserved hypothetical protein
225-64	non-synonymous	t	c	1640605	SA1437	conserved hypothetical protein
225-65	non-synonymous	g	t	1644183	SA1441	SA1441~hypothetical protein, similar to protease
225-66	intergenic	t	c	1657571	intergenic	
225-67	non-synonymous	g	a	1692825	SA1487	folylpolyglutamate synthase
225-68	non-synonymous	t	a	1741284	SA1525	DNA polymerase III, alpha chain
225-69	synonymous	t	c	1773455	SA1550	tyrosyl-tRNA synthetase
225-70	non-synonymous	g	a	1775587	SA1552	hypothetical protein
225-71	intergenic	g	a	1804206	intergenic	
225-72	non-synonymous	g	a	1813596	SA1577	SA1577~hypothetical protein, similar to FmtB protein
225-73 [1]	intergenic	a	t	1823773	intergenic	
225-74	intergenic	g	a	1853433	intergenic	
225-75	intergenic	c	a	1861798	intergenic	
225-76	intergenic	t	g	1861834	intergenic	
225-77	intergenic	a	g	1865166	intergenic	
225-78	non-synonymous	c	a	1876774	SA1642	extracellular enterotoxin type G precursor
225-79	intergenic	c	t	1899592	intergenic	
225-80	non-synonymous	c	t	1932629	SA1685	SA1685~hypothetical protein, similar to A/G-specific adenine glycosylase
225-81	intergenic	c	t	1977862	intergenic	
225-82	non-synonymous	a	t	2020099	SA1766	hypothetical protein
225-83	non-synonymous	t	c	2073737	SA1836	GroEL protein
225-84	non-synonymous	a	g	2080800	SA1843	accessory gene regulator C
225-85	intergenic	t	c	2089271	intergenic	

225-86	synonymous	c	t	2137216	SA1885	SA1885~hypothetical protein, similar to ATP-dependent RNA helicase
225-87	synonymous	g	a	2185831	SA1935	SA1935~similar to amidase (HmrA)
225-88	synonymous	a	t	2256228	SA1985	hypothetical protein
225-89	intergenic	g	a	2278973	intergenic	
225-90 [1]	intergenic	t	a	2289677	intergenic	
225-91 [1]	intergenic	g	a	2289683	intergenic	
225-92 [1]	intergenic	g	a	2289684	intergenic	
225-93	non-synonymous	t	g	2298378	SA2028	preprotein translocase SecY subunit
225-94	synonymous	c	t	2323440	SA2059	hypothetical protein
225-95	non-synonymous	c	a	2363258	SA2102	SA2102~formate dehydrogenase homolog
225-96	non-synonymous	c	t	2385280	SA2121	imidazolonepropionase
225-97	synonymous	g	a	2396570	SA2131	conserved hypothetical protein
225-98	non-synonymous	t	a	2425926	SA2158	SA2158~hypothetical protein, similar to TpgX protein
225-99	non-synonymous	c	t	2446513	SA2177	conserved hypothetical protein
225-100	intergenic	a	g	2477881	intergenic	
225-101	non-synonymous	g	t	2495482	SA2222	SA2222~hypothetical protein, similar to bicyclomycin resistance protein
225-102	synonymous	t	c	2510392	SA2235	glycine betaine/carnitine/choline ABC transporter opuCC
225-103	intergenic	a	c	2544000	intergenic	
225-104	non-synonymous	a	g	2579960	SA2296	SA2296~hypothetical protein, similar to transcriptional regulator, MerR
225-105	synonymous	g	a	2605069	SA2320	SA2320~hypothetical protein, similar to regulatory protein pfoR
225-106	non-synonymous	g	a	2730381	SA2431	immunodominant antigen B
225-107	non-synonymous	g	t	2746905	SA2441	SA2441~hypothetical protein, similar to lipopolysaccharide biosynthesis protein
225-108	non-synonymous	g	a	2796206	SA2485	hypothetical protein
225-109	intergenic	g	a	2807494	intergenic	
225-110	synonymous	a	g	2813010	SA2501	possible thiophene and furan oxidation protein
**Indels.**						
225-111	deletion (4 nt)	-	-	119405	SA0104	SA0104~hypothetical protein, similar to transcription regulator GntR
225-112	deletion (48 nt)	-	-	123024	SA0107	Immunoglobulin G binding protein A precursor

225-113	deletion (43 nt)	-	-	888870	intergenic	
225-114	deletion (18 nt)	-	-	1021776	SA0901	serine protease V8 protease; glutamyl endopeptidase
225-115	deletion (134 nt)	-	-	1117974	intergenic	
225-116	deletion (231 nt)	-	-	1440180	SA1267	SA1267~hypothetical protein, similar to streptococcal adhesin emb
225-117	deletion (8 nt)	-	-	1441052	SA1267	SA1267~hypothetical protein, similar to streptococcal adhesin emb
225-118	deletion (1 nt)	-	-	1871250	intergenic	
225-119	insertion (2460 nt)	-	-	2108319	intergenic	
225-120	deletion (69 nt)	-	-	2349731	SA2091	hypothetical protein

[1] detected only by Solexa sequencing

[2] detected only by 454 sequencing

Table S8b. Derived traits in the JH strain.

Polymorphism	Quality	Ancestral	Derived	Position in N315 genome			Product
**Substitutions.**							
JH-1	silent	g	a	27546	SA0019	JH9 only	conserved hypothetical protein
JH-2	intergenic	t	c	102548	intergenic		
JH-3	missense	a	t	118953	SA0104		SA0104~hypothetical protein, similar to transcription regulator GntR
JH-4	missense	t	c	145331	SA0126		SA0126~hypothetical protein, similar to capsular polysaccharide synthesis
JH-5	silent	t	c	165974	SA0143		alcohol-acetaldehyde dehydrogenase
JH-6	missense	t	c	213845	SA0182		SA0182~hypothetical protein, similar to indole-3-pyruvate decarboxylas
JH-7	missense	c	a	218569	SA0185	JH9 only	conserved hypothetical protein
JH-8	missense	t	c	255175	SA0215	JH9 only	SA0215~hypothetical protein, similar to two-component response regulator
JH-9	missense	c	t	266561	SA0222		staphylocoagulase precursor
JH-10	silent	t	c	294331	SA0242		SA0242~hypothetical protein, similar to xylitol dehydrogenase
JH-11	missense	a	g	307523	SA0252		holin-like protein LrgA
JH-12	missense	g	a	332327	SA0275		conserved hypothetical protein
JH-13	silent	c	t	348810	SA0294		SA0294~hypothetical protein, similar to branched-chain amino acid
JH-14	missense	c	a	399348	SA0342		SA0342~acetyl-CoA C-acetyltransferase homologue
JH-15	silent	g	a	416360	SA0358		conserved hypothetical protein
JH-16	missense	a	c	493068	SA0430		glutamate synthase large subunit
JH-17	missense	g	a	504456	SA0437		conserved hypothetical protein
JH-18	intergenic	t	c	505890	intergenic		
JH-19	missense	a	g	545105	SA0471		SA0471~cysteine synthase (o-acetylserine sulfhydrylase) homologue
JH-20	intergenic	g	a	565586	intergenic		
JH-21	missense	g	t	581030	SA0500	JH9 only	RNA polymerase beta chain
JH-22	missense	g	t	581036	SA0500	JH9 only	RNA polymerase beta chain
JH-23	missense	g	t	581048	SA0500	JH9 only	RNA polymerase beta chain
JH-24	missense	g	t	581053	SA0500	JH9 only	RNA polymerase beta chain
JH-25	missense	g	a	585867	SA0501	JH9 only	RNA polymerase beta-prime chain

JH-26	silent	c	t	604546	SA0518		conserved hypothetical protein
JH-27	silent	t	a	607698	SA0519		Ser-Asp rich fibrinogen-binding, bone sialoprotein-binding protein
JH-28	silent	c	t	607755	SA0519		Ser-Asp rich fibrinogen-binding, bone sialoprotein-binding protein
JH-29	silent	c	t	607821	SA0519		Ser-Asp rich fibrinogen-binding, bone sialoprotein-binding protein
JH-30	intergenic	g	a	621945	intergenic	JH9 only	
JH-31	silent	t	c	674706	SA0582	JH9 only	SA0582~hypothetical protein, similar to Na+/H+ antiporter
JH-32	missense	c	t	711484	SA0617		ABC transporter permease
JH-33	missense	c	t	712573	SA0617	JH9 only	ABC transporter permease
JH-34	intergenic	a	g	716794	intergenic		
JH-35	intergenic	t	c	720375	intergenic		
JH-36	intergenic	t	c	727412	intergenic		
JH-37	intergenic	g	c	731774	intergenic		
JH-38	missense	g	a	743949	SA0650		quinolone resistance protein
JH-39	missense	a	g	764808	SA0673		conserved hypothetical protein
JH-40	missense	a	g	772550	SA0677		SA0677~hypothetical protein, similar to choline transport ATP-binding
JH-41	silent	t	c	784439	SA0686		ribonuceloside diphosphate reductase major subunit
JH-42	missense	g	c	849288	SA0742		fibrinogen-binding protein A, clumping factor
JH-43	missense	c	a	857780	SA0749		hypothetical protein
JH-44	missense	t	c	861844	SA0754		SA0754~hypothetical protein, similar to lactococcal prophage ps3
JH-45	silent	t	c	876044	SA0769		ABC transporter ATP-binding protein homologue~SA0769
JH-46	missense	t	a	884267	SA0778		conserved hypothetical protein
JH-47	missense	t	c	900904	SA0794		DltB membrane protein
JH-48	intergenic	t	c	902631	intergenic		
JH-49	missense	g	a	942600	SA0831		coenzyme A disulfide reductase
JH-50	missense	c	t	948664	SA0835		ClpB chaperone homologue~SA0835
JH-51	intergenic	c	t	965657	intergenic		
JH-52	missense	a	g	972030	SA0857		SA0857~hypothetical protein, similar to negative regulator of
JH-53	silent	t	a	981677	SA0867		SA0867~hypothetical protein, similar to Mg2+ transporter
JH-54	intergenic	a	g	1006949	intergenic		
JH-55	intergenic	g	a	1039232	intergenic		

JH-56	intergenic	t	c	1052987	intergenic		
JH-57	silent	g	t	1056696	intergenic		
JH-58	silent	t	c	1058468	intergenic		
JH-59	silent	t	c	1112797	SA0983		conserved hypothetical protein
JH-60	missense	c	a	1120582	SA0990		DNA-dependent DNA polymerase beta chain
JH-61	missense	g	a	1122903	SA0991		MutS-like protein
JH-62	missense	g	t	1165842	SA1029	JH1 only	cell division protein
JH-63	missense	c	t	1189892	SA1051		SA1051~hypothetical protein, similar to fibrinogen binding protein
JH-64	silent	t	g	1211429	SA1070		ATP-dependent DNA helicase
JH-65	missense	g	a	1240706	SA1094		glucose-inhibited division protein gid
JH-66	missense	c	t	1242039	SA1095		SA1095~site-specific recombinase XerC homolog
JH-67	missense	a	g	1245341	SA1099		30S ribosomal protein S2
JH-68	missense	g	t	1282852	SA1129	JH9 only	conserved hypothetical protein
JH-69	missense	g	a	1300480	SA1142		aerobic glycerol-3-phosphate dehydrogenase
JH-70	missense	a	g	1304064	SA1147	JH9 only	SA1147~hypothetical protein, similar to GTP-binding protein proteinase
JH-71	intergenic	g	t	1309884	intergenic		
JH-72	intergenic	a	g	1310275	intergenic		
JH-73	missense	a	g	1356575	SA1189		topoisomerase IV subunit A
JH-74	silent	c	t	1365399	SA1193		oxacillin resistance-related FmtC protein
JH-75	missense	g	c	1370028	SA1197		prephenate dehydrogenase
JH-76	silent	a	g	1376795	SA1203		phosphoriborylanthranilate isomerase
JH-77	missense	g	a	1384692	SA1211		oligopeptide transporter putative ATPase domain
JH-78	missense	c	a	1388648	SA1216		SA1216~hypothetical protein, similar to oligoendopeptidase
JH-79	missense	g	a	1412334	SA1239		branched-chain amino acid carrier protein
JH-80	missense	t	c	1467590	SA1268		SA1268~hypothetical protein, similar to streptococcal adhesin emb
JH-81	silent	a	g	1476627	SA1273		SA1273~hypothetical protein, similar to 5-3 exonuclease
JH-82	missense	g	a	1478486	SA1274		conserved hypothetical protein
JH-83	intergenic	c	a	1480267	intergenic		
JH-84	missense	g	t	1503480	SA1297		3-phosphoshikimate 1-carboxyvinyltransferase
JH-85	missense	a	g	1526319	SA1318		hypothetical protein

JH-86	missense	c	t	1526328	SA1318		hypothetical protein
JH-87	silent	a	g	1526582	SA1319		hypothetical protein
JH-88	missense	c	t	1556932	SA1347		branched-chain alpha-keto acid dehydrogenase E1
JH-89	missense	g	t	1564462	SA1354		SA1354~hypothetical protein, similar to exodeoxyribonuclease large subunit
JH-90	missense	t	a	1598094	SA1391		DNA primase
JH-91	silent	g	a	1624269	SA1416		SA1416~hypothetical protein, similar to ComEC late competence
JH-92	silent	t	a	1700992	SA1492		delta-aminolevulinic acid dehydratase
JH-93	silent	a	g	1719792	SA1510	JH9 only	glyceraldehyde 3-phosphate dehydrogenase 2
JH-94	missense	c	a	1720818	SA1511		conserved hypothetical protein
JH-95	missense	t	c	1752491	SA1533		SA1533~acetate kinase homolog
JH-96	missense	g	a	1753184	SA1534		conserved hypothetical protein
JH-97	silent	c	t	1790881	SA1562		DNA translocase stage III sporulation prot homolog~SA1562
JH-98	missense	t	c	1811845	SA1577		SA1577~hypothetical protein, similar to FmtB protein
JH-99	silent	t	c	1816821	SA1579		leucyl-rRNA synthetase
JH-100	intergenic	c	t	1850820	intergenic		
JH-101	silent	c	t	1854089	SA1619		hypothetical protein
JH-102	missense	a	t	1888014	SA1653		signal transduction protein TRAP
JH-103	missense	g	c	1893057	SA1659	JH9 only	SA1659~peptidyl-prolyl cis/trans isomerase homolog
JH-104	missense	c	t	1909088	SA1672		conserved hypothetical protein
JH-105	missense	t	c	1948612	SA1702	JH9 only	conserved hypothetical protein
JH-106	missense	a	t	1956946	SA1711		SA1711~hypothetical protein, similar to DNA-damage inducible protein
JH-107	silent	t	g	1965709	SA1718		high affinity proline permease
JH-108	missense	t	c	2002508	SA1749		SA1749~hypothetical protein, similar to aspartate transaminase protein
JH-109	missense	t	c	2074787	SA1838		conserved hypothetical protein
JH-110	silent	t	c	2077993	SA1841		hypothetical protein
JH-111	silent	t	c	2087598	intergenic		
JH-112	intergenic	t	c	2130557	intergenic		
JH-113	missense	g	t	2181781	SA1932		SA1932~hypothetical protein, similar to hypothetical protein T13D8.31
JH-114	intergenic	c	a	2225718	intergenic		
JH-115	intergenic	c	t	2243315	intergenic		

JH-116	missense	a	t	2264897	SA1992		PTS system, lactose-specific IIBC component
JH-117	intergenic	a	g	2273403	intergenic		
JH-118	silent	c	t	2277234	SA2005		conserved hypothetical protein
JH-119	missense	c	t	2307851	SA2047	JH9 only	50S ribosomal protein L3
JH-120	missense	c	t	2307876	SA2047	JH9 only	50S ribosomal protein L3
JH-121	silent	t	c	2349313	SA2091	JH9 only	hypothetical protein
JH-122	missense	c	t	2354954	SA2094	JH9 only	SA2094~hypothetical protein, similar to Na+/H+ antiporter
JH-123	silent	a	t	2363617	SA2102		SA2102~formate dehydrogenase homolog
JH-124	silent	t	c	2382895	SA2119	JH9 only	SA2119~hypothetical protein, similar to dehydrogenase
JH-125	intergenic	t	c	2391175	intergenic	JH9 only	
JH-126	missense	t	a	2405262	SA2140		SA2140~hypothetical protein, similar to esterase
JH-127	missense	g	a	2405263	SA2140		SA2140~hypothetical protein, similar to esterase
JH-128	missense	c	a	2412803	SA2146		TcaA protein
JH-129	silent	g	a	2455248	SA2185		respiratory nitrate reductase alpha chain
JH-130	intergenic	g	t	2456525	intergenic		
JH-131	missense	a	c	2462243	SA2191		SA2191~hypothetical protein, similar to NirC
JH-132	intergenic	t	c	2507705	intergenic	JH9 only	
JH-133	intergenic	g	a	2536664	intergenic		
JH-134	silent	a	g	2571397	intergenic		
JH-135	missense	g	a	2584776	SA2300		SA2300~hypothetical protein, similar to glucarate transporter
JH-136	missense	c	a	2596593	SA2311		SA2311~hypothetical protein, similar to NAD(P)H-flavin oxidoreductase
JH-137	silent	a	g	2604820	SA2320	JH9 only	SA2320~hypothetical protein, similar to regulatory protein pfoR
JH-138	silent	c	t	2624449	SA2337		SA2337~ferrous iron transport protein B homolog
JH-139	silent	g	a	2636833	SA2347		SA2347~hypothetical protein, similar to aspartate aminotransferase
JH-140	missense	g	a	2640311	SA2349		squalene desaturase
JH-141	silent	a	g	2667614	SA2379		SA2379~hypothetical protein, similar to transcriptional regulator tetR-family
JH-142	intergenic	c	t	2693646	intergenic		
JH-143	missense	t	c	2703993	SA2411		SA2411~hypothetical protein, similar to magnesium citrate secondary
JH-144	silent	a	g	2719048	SA2423		Clumping factor B
JH-145	missense	g	t	2734245	SA2434		SA2434~fructose phosphotransferase system enzyme fruA homolog

JH-146	intergenic	c	t	2765563	intergenic		
JH-147	missense	a	c	2765943	SA2453		SA2453~hypothetical protein, similar to peptide methionine sulfoxide
JH-148	intergenic	g	a	2774212	intergenic		
JH-149	missense	c	t	2795273	SA2483		hypothetical protein
JH-150	intergenic	t	c	2800096	intergenic		
JH-151	silent	c	t	2808325	SA2497		hypothetical protein
**Indels.**							
JH-152	deletion (5 nt)	-	-	100147	SA0089		SA0089~hypothetical protein, similar to DNA helicase
JH-153	deletion (59 nt)	-	-	188439	SAS005		hypothetical protein
JH-154	deletion (1 nt)	-	-	194003	SA0171	JH9 only	NAD-dependent formate dehydrogenase
JH-155	deletion (1 nt)	-	-	226043	intergenic		
JH-156	insertion (1 nt)	-	-	285716	intergenic		
JH-157	deletion (110 nt)	-	-	311527	intergenic		
JH-158	Insertion (43106 nt)	-	-	367380	SA0309		glycerol ester hydrolase
JH-159	insertion (1 nt)	-	-	382385	SA0324		hypothetical protein
JH-160	insertion (1 nt)	-	-	393979	intergenic		
JH-161	deletion (1 nt)	-	-	470470	intergenic	JH9 only	
JH-162	deletion (109 nt)	-	-	507773	intergenic		
JH-163	deletion (1 nt)	-	-	577659	intergenic		
JH-164	deletion (1 nt)	-	-	604200	intergenic		conserved hypothetical protein
JH-165	insertion (1 nt)	-	-	605021	intergenic		
JH-166	insertion (1 nt)	-	-	666140	intergenic		
JH-167	insertion (73 nt)	-	-	678226	intergenic		
JH-168	insertion (1520 nt)	-	-	712801	intergenic		
JH-169	insertion (2 nt)	-	-	713010	intergenic		
JH-170	deletion (1 nt)	-	-	725837	SA0629	JH1 only	conserved hypothetical protein
JH-171	insertion (4 nt)	-	-	743408	intergenic		
JH-172	insertion (1 nt)	-	-	928680	intergenic	JH9 only	
JH-173	insertion (45503 nt)	-	-	997069	intergenic		

JH-174	deletion (1 nt)	-	-	1052889	intergenic		
JH-175	insertion (1 nt)	-	-	1294678	SA1138	JH9 only	DNA mismatch repair protein
JH-176	deletion (1 nt)	-	-	1413187	intergenic		
JH-177	insertion (2 nt)	-	-	1424822	SA1249	JH1 only	hypothetical protein
JH-178	insertion (1 nt)	-	-	1424822	SA1249	JH9 only	hypothetical protein
JH-179	insertion (1 nt)	-	-	1634817	SA1431		conserved hypothetical protein
JH-180	deletion (25 nt)	-	-	1685817	intergenic		
JH-181	deletion (114 nt)	-	-	1804088	intergenic		
JH-182	insertion (1 nt)	-	-	1822742	intergenic		
JH-183	deletion (1 nt)	-	-	1823773	intergenic		
JH-184	insertion (1 nt)	-	-	1851581	intergenic		
JH-185	deletion (1 nt)	-	-	1893514	SA1659	JH9 only	SA1659~peptidyl-prolyl cis/trans isomerase homolog
JH-186	insertion (1520 nt)	-	-	1901479	intergenic		
JH-187	deletion (72 nt)	-	-	1916199	intergenic		
JH-188	insertion (1 nt)	-	-	1917002	intergenic	JH9 only	
JH-189	deletion (59 nt)	-	-	1950673	intergenic		
JH-190	replacement (43799 nt)	-	-	2005721	SAS058		hypothetical protein
JH-191	deletion (1 nt)	-	-	2080665	SA1843	JH9 only	accessory gene regulator C
JH-192	deletion (2 nt)	-	-	2289683	intergenic	JH1 only	
JH-193	deletion (3 nt)	-	-	2289683	intergenic	JH9 only	
JH-194	insertion (1520 nt)	-	-	2378387	intergenic		
JH-195	deletion (60 nt)	-	-	2389170	intergenic		
JH-196	insertion (1 nt)	-	-	2398032	intergenic		
JH-197	deletion (3 nt)	-	-	2413041	intergenic		
JH-198	deletion (1 nt)	-	-	2429817	intergenic		
JH-199	deletion (50 nt)	-	-	2452914	SA2185		respiratory nitrate reductase alpha chain
JH-200	insertion (1 nt)	-	-	2466192	SA2195		conserved hypothetical protein
JH-201	deletion (1 nt)	-	-	2499489	intergenic		
JH-202	deletion (1520 nt)	-	-	2566722	intergenic		
JH-203	deletion (1 nt)	-	-	2647605	SA2355		conserved hypothetical protein

JH-204	deletion (2 nt)	-	-	2774079	SAS090	hypothetical protein

Table S8c. Derived traits shared by 04-02981 and the JH strain.

Polymorphism	Quality	Ancestral	Derived	Position in N315 genome	ORF	Product
**Substitutions.**						
225/JH1/JH9-1	non-synonymous	c	t	7255	SA0006	DNA gyrase subunit A
225/JH1/JH9-2	intergenic	g	t	36145	intergenic	
225/JH1/JH9-3	synonymous	g	a	48885	SA0039	methicillin resistance protein
225/JH1/JH9-4	intergenic	g	t	53495	intergenic	
225/JH1/JH9-5	synonymous	g	t	54572	intergenic	
225/JH1/JH9-6	synonymous	t	c	54574	intergenic	
225/JH1/JH9-7	synonymous	g	a	54575	intergenic	
225/JH1/JH9-8	synonymous	a	t	67944	SA0059	hypothetical protein
225/JH1/JH9-9	non-synonymous	t	c	67947	SA0059	hypothetical protein
225/JH1/JH9-10	non-synonymous	t	c	67948	SA0059	hypothetical protein
225/JH1/JH9-11	non-synonymous	a	t	67953	SA0059	hypothetical protein
225/JH1/JH9-12	non-synonymous	t	c	67955	SA0059	hypothetical protein
225/JH1/JH9-13	non-synonymous	c	t	67967	SA0059	hypothetical protein
225/JH1/JH9-14	non-synonymous	a	c	67971	SA0059	hypothetical protein
225/JH1/JH9-15	non-synonymous	t	c	68010	SA0059	hypothetical protein
225/JH1/JH9-16	non-synonymous	t	g	68011	SA0059	hypothetical protein
225/JH1/JH9-17	non-synonymous	c	t	68029	SA0059	hypothetical protein
225/JH1/JH9-18	synonymous	a	g	68103	SA0059	hypothetical protein
225/JH1/JH9-19	synonymous	c	t	68169	SA0059	hypothetical protein
225/JH1/JH9-20	synonymous	t	g	68181	SA0059	hypothetical protein
225/JH1/JH9-21	non-synonymous	t	a	68224	SA0059	hypothetical protein
225/JH1/JH9-22	intergenic	t	a	76834	intergenic	
225/JH1/JH9-23	non-synonymous	a	g	114285	SA0100	conserved hypothetical protein
225/JH1/JH9-24	intergenic	a	g	120568	intergenic	
225/JH1/JH9-25	intergenic	a	c	142077	intergenic	

225/JH1/JH9-26	synonymous	g	a	145095	SA0125	SA0125~hypothetical protein, similar to Eps(Exopolysaccharide)G
225/JH1/JH9-27	intergenic	c	a	150897	intergenic	
225/JH1/JH9-28	intergenic	c	a	167251	intergenic	
225/JH1/JH9-29	synonymous	a	g	175947	SA0152	capsular polysaccharide synthesis enzyme Cap5I
225/JH1/JH9-30	non-synonymous	g	a	192968	SA0169	SA0169~hypothetical protein, similar to acyl-CoA dehydrogenase family
225/JH1/JH9-31	non-synonymous	g	a	196669	SA0172	SA0172~hypothetical protein, similar to integral membrane protein
225/JH1/JH9-32	non-synonymous	t	g	209160	SA0178	N-acetylglutamate gamma-semialdehyde dehydrogenase
225/JH1/JH9-33	intergenic	t	g	221793	intergenic	
225/JH1/JH9-34	intergenic	a	g	229242	intergenic	
225/JH1/JH9-35	non-synonymous	a	g	268323	SA0223	SA0223~acetyl-CoA acetyltransferase homologue
225/JH1/JH9-36	intergenic	g	t	311848	intergenic	
225/JH1/JH9-37	intergenic	c	t	350165	intergenic	
225/JH1/JH9-38	synonymous	c	t	358996	SA0303	SA0303~hypothetical protein, similar to sodium-coupled permease
225/JH1/JH9-39	synonymous	t	a	381103	SA0323	conserved hypothetical protein
225/JH1/JH9-40	intergenic	g	a	388022	intergenic	
225/JH1/JH9-41	intergenic	g	t	388777	intergenic	
225/JH1/JH9-42	non-synonymous	t	g	403354	SA0344	5-methyltetrahydropteroyltriglutamate-homocystei ne methyltransferase
225/JH1/JH9-43	non-synonymous	g	t	431698	SA0373	xanthine phosphoribosyltransferase
225/JH1/JH9-44	synonymous	c	t	432241	SA0374	xanthine permease
225/JH1/JH9-45	non-synonymous	c	t	443385	SA0384	exotoxin 8
225/JH1/JH9-46	non-synonymous	t	c	452159	SA0391	probable type I site-specific deoxyribonuclease LldI chain
225/JH1/JH9-47	synonymous	g	t	479340	SA0417	SA0417~hypothetical protein, similar to sodium-dependent transporter
225/JH1/JH9-48	synonymous	t	c	488552	SA0428	conserved hypothetical protein
225/JH1/JH9-49	non-synonymous	a	g	519966	SA0448	methionyl-tRNA synthetase
225/JH1/JH9-50	intergenic	c	t	526898	intergenic	
225/JH1/JH9-51	non-synonymous	c	a	534044	SA0461	transcription-repair coupling factor
225/JH1/JH9-52	non-synonymous	a	t	537348	SA0463	conserved hypothetical protein
225/JH1/JH9-53	synonymous	t	c	615807	SA0521	Ser-Asp rich fibrinogen-binding, bone sialoprotein-binding protein
225/JH1/JH9-54	synonymous	t	c	615810	SA0521	Ser-Asp rich fibrinogen-binding, bone sialoprotein-binding protein
225/JH1/JH9-55	synonymous	c	t	615816	SA0521	Ser-Asp rich fibrinogen-binding, bone sialoprotein-binding protein

225/JH1/JH9-56	synonymous	c	t	615840	SA0521	Ser-Asp rich fibrinogen-binding, bone sialoprotein-binding protein
225/JH1/JH9-57	synonymous	c	t	615846	SA0521	Ser-Asp rich fibrinogen-binding, bone sialoprotein-binding protein
225/JH1/JH9-58	synonymous	t	c	615861	SA0521	Ser-Asp rich fibrinogen-binding, bone sialoprotein-binding protein
225/JH1/JH9-59	synonymous	t	c	615864	SA0521	Ser-Asp rich fibrinogen-binding, bone sialoprotein-binding protein
225/JH1/JH9-60	synonymous	t	c	615882	SA0521	Ser-Asp rich fibrinogen-binding, bone sialoprotein-binding protein
225/JH1/JH9-61	synonymous	t	c	615888	SA0521	Ser-Asp rich fibrinogen-binding, bone sialoprotein-binding protein
225/JH1/JH9-62	synonymous	c	t	615924	SA0521	Ser-Asp rich fibrinogen-binding, bone sialoprotein-binding protein
225/JH1/JH9-63	synonymous	a	g	615927	SA0521	Ser-Asp rich fibrinogen-binding, bone sialoprotein-binding protein
225/JH1/JH9-64	synonymous	c	t	615936	SA0521	Ser-Asp rich fibrinogen-binding, bone sialoprotein-binding protein
225/JH1/JH9-65	synonymous	g	a	615963	SA0521	Ser-Asp rich fibrinogen-binding, bone sialoprotein-binding protein
225/JH1/JH9-66	synonymous	c	t	616005	SA0521	Ser-Asp rich fibrinogen-binding, bone sialoprotein-binding protein
225/JH1/JH9-67	synonymous	t	c	616008	SA0521	Ser-Asp rich fibrinogen-binding, bone sialoprotein-binding protein
225/JH1/JH9-68	synonymous	t	c	616032	SA0521	Ser-Asp rich fibrinogen-binding, bone sialoprotein-binding protein
225/JH1/JH9-69	synonymous	t	c	616056	SA0521	Ser-Asp rich fibrinogen-binding, bone sialoprotein-binding protein
225/JH1/JH9-70	synonymous	c	t	616062	SA0521	Ser-Asp rich fibrinogen-binding, bone sialoprotein-binding protein
225/JH1/JH9-71	synonymous	c	t	616068	SA0521	Ser-Asp rich fibrinogen-binding, bone sialoprotein-binding protein
225/JH1/JH9-72	synonymous	c	t	616074	SA0521	Ser-Asp rich fibrinogen-binding, bone sialoprotein-binding protein
225/JH1/JH9-73	synonymous	t	c	616077	SA0521	Ser-Asp rich fibrinogen-binding, bone sialoprotein-binding protein
225/JH1/JH9-74	synonymous	c	t	616086	SA0521	Ser-Asp rich fibrinogen-binding, bone sialoprotein-binding protein
225/JH1/JH9-75	synonymous	a	g	616089	SA0521	Ser-Asp rich fibrinogen-binding, bone sialoprotein-binding protein
225/JH1/JH9-76	synonymous	c	t	616095	SA0521	Ser-Asp rich fibrinogen-binding, bone sialoprotein-binding protein
225/JH1/JH9-77	synonymous	t	c	616104	SA0521	Ser-Asp rich fibrinogen-binding, bone sialoprotein-binding protein
225/JH1/JH9-78	synonymous	g	a	616125	SA0521	Ser-Asp rich fibrinogen-binding, bone sialoprotein-binding protein
225/JH1/JH9-79	synonymous	t	c	616131	SA0521	Ser-Asp rich fibrinogen-binding, bone sialoprotein-binding protein
225/JH1/JH9-80	synonymous	t	c	616134	SA0521	Ser-Asp rich fibrinogen-binding, bone sialoprotein-binding protein
225/JH1/JH9-81	synonymous	a	g	616161	SA0521	Ser-Asp rich fibrinogen-binding, bone sialoprotein-binding protein
225/JH1/JH9-82	non-synonymous	a	g	708026	SA0613	hypothetical protein
225/JH1/JH9-83	intergenic	c	a	731851	intergenic	
225/JH1/JH9-84	intergenic	t	c	823708	intergenic	
225/JH1/JH9-85	intergenic	a	g	826105	intergenic	

225/JH1/JH9-86	non-synonymous	c	t	858626	SA0750	conserved hypothetical protein
225/JH1/JH9-87	non-synonymous	a	g	922427	SA0818	ornithine aminotransferase
225/JH1/JH9-88	non-synonymous	a	t	942047	SA0831	coenzyme A disulfide reductase
225/JH1/JH9-89	non-synonymous	c	g	943797	SA0833	conserved hypothetical protein
225/JH1/JH9-90	synonymous	t	c	974926	SA0859	SA0859~thimet oligopeptidase homologue
225/JH1/JH9-91	non-synonymous	c	t	979946	SA0866	conserved hypothetical protein
225/JH1/JH9-92	intergenic	t	c	1003591	intergenic	
225/JH1/JH9-93	non-synonymous	c	t	1011207	SA0890	conserved hypothetical protein
225/JH1/JH9-94	non-synonymous	g	a	1041880	SA0916	SA0916~hypothetical protein, similar to phosphoribosylaminoimidazole carboxylase PurE
225/JH1/JH9-95	non-synonymous	a	g	1056488	SA0930	hypothetical protein
225/JH1/JH9-96	synonymous	g	a	1056495	SA0930	hypothetical protein
225/JH1/JH9-97	non-synonymous	a	t	1056498	SA0930	hypothetical protein
225/JH1/JH9-98	non-synonymous	c	t	1087124	SA0959	GTP-binding elongation factor homolog~SA0959
225/JH1/JH9-99	non-synonymous	t	a	1112554	SA0983	conserved hypothetical protein
225/JH1/JH9-100	intergenic	a	t	1134428	intergenic	
225/JH1/JH9-101	intergenic	c	a	1141915	intergenic	
225/JH1/JH9-102	non-synonymous	t	a	1146175	SA1012	ornithine carbamoyltransferase
225/JH1/JH9-103	synonymous	a	g	1205544	SA1065	SA1065~ribulose-5-phosphate 3-epimerase homolog
225/JH1/JH9-104	intergenic	g	t	1215512	intergenic	
225/JH1/JH9-105	non-synonymous	t	c	1264963	SA1115	riboflavin kinase / FAD synthase ribC
225/JH1/JH9-106	intergenic	g	t	1309079	intergenic	
225/JH1/JH9-107	non-synonymous	g	t	1313764	SAS039	hypothetical protein
225/JH1/JH9-108	non-synonymous	t	a	1327469	SA1165	threonine synthase
225/JH1/JH9-109	intergenic	t	a	1346524	intergenic	
225/JH1/JH9-110	non-synonymous	c	t	1356563	SA1189	topoisomerase IV subunit A
225/JH1/JH9-111	synonymous	t	c	1356700	SA1189	topoisomerase IV subunit A
225/JH1/JH9-112	non-synonymous	c	g	1383243	SA1209	conserved hypothetical protein
225/JH1/JH9-113	non-synonymous	g	t	1400220	SA1225	aspartokinase II
225/JH1/JH9-114	non-synonymous	g	a	1403180	SA1228	dihydrodipicolinate reductase
225/JH1/JH9-115	non-synonymous	t	c	1416680	SA1243	ABC transporter homolog~SA1243

225/JH1/JH9-116	synonymous	g	a	1439637	SA1267	SA1267~hypothetical protein, similar to streptococcal adhesin emb
225/JH1/JH9-117	synonymous	c	t	1440457	SA1267	SA1267~hypothetical protein, similar to streptococcal adhesin emb
225/JH1/JH9-118	non-synonymous	c	a	1457298	SA1267	SA1267~hypothetical protein, similar to streptococcal adhesin emb
225/JH1/JH9-119	non-synonymous	t	c	1459382	SA1268	SA1268~hypothetical protein, similar to streptococcal adhesin emb
225/JH1/JH9-120	non-synonymous	t	c	1467201	SA1268	SA1268~hypothetical protein, similar to streptococcal adhesin emb
225/JH1/JH9-121	non-synonymous	c	t	1488775	SA1283	PBP2
225/JH1/JH9-122	intergenic	t	c	1515295	intergenic	
225/JH1/JH9-123	intergenic	a	t	1548690	intergenic	
225/JH1/JH9-124	synonymous	g	a	1551033	SA1342	phosphogluconate dehydrogenase
225/JH1/JH9-125	non-synonymous	c	t	1584734	SA1379	conserved hypothetical protein
225/JH1/JH9-126	synonymous	c	t	1594582	SA1389	conserved hypothetical protein
225/JH1/JH9-127	non-synonymous	c	t	1606599	SA1401	conserved hypothetical protein
225/JH1/JH9-128	non-synonymous	g	a	1658713	SA1454	conserved hypothetical protein
225/JH1/JH9-129	non-synonymous	c	a	1720010	SA1510	glyceraldehyde 3-phosphate dehydrogenase 2
225/JH1/JH9-130	non-synonymous	t	c	1756121	SA1537	SA1537~hypothetical protein, similar to thiamine biosynthesis protein
225/JH1/JH9-131	synonymous	t	a	1763660	SA1542	SA1542~hypothetical protein, similar to glycerophosphoryl diester phosphodiesterase
225/JH1/JH9-132	non-synonymous	g	a	1781077	SA1554	acetyl-CoA synthetase
225/JH1/JH9-133	intergenic	c	a	1799357	intergenic	
225/JH1/JH9-134	non-synonymous	t	g	1811463	SA1577	SA1577~hypothetical protein, similar to FmtB protein
225/JH1/JH9-135	intergenic	g	a	1815605	intergenic	
225/JH1/JH9-136	synonymous	a	t	1840149	SA1604	truncated transposase
225/JH1/JH9-137	non-synonymous	g	c	1895316	SA1661	conserved hypothetical protein
225/JH1/JH9-138	non-synonymous	c	t	1925373	SA1679	SA1679~hypothetical protein, similar to D-3-phosphoglycerate dehydrogenase
225/JH1/JH9-139	non-synonymous	c	a	1926464	SA1680	conserved hypothetical protein
225/JH1/JH9-140	synonymous	c	t	1933684	SA1686	conserved hypothetical protein
225/JH1/JH9-141	non-synonymous	t	c	1939119	SA1691	SA1691~hypothetical protein, similar to penicillin-binding protein 1A/1B
225/JH1/JH9-142	intergenic	g	t	1955289	intergenic	
225/JH1/JH9-143	non-synonymous	a	t	1989982	SA1736	aldehyde dehydrogenase
225/JH1/JH9-144	intergenic	a	g	1995643	intergenic	
225/JH1/JH9-145	non-synonymous	g	a	2049937	SA1811	truncated beta-hemolysin

225/JH1/JH9-146	intergenic	c	t	2052862	intergenic	
225/JH1/JH9-147	intergenic	g	a	2079428	intergenic	
225/JH1/JH9-148	synonymous	t	c	2104813	SA1863	3-isopropylmalate dehydrogenase
225/JH1/JH9-149	non-synonymous	c	t	2120561	SA1872	sigmaB regulation protein RsbU
225/JH1/JH9-150	non-synonymous	t	c	2129221	SA1881	probable potassium-transporting ATPase A chain
225/JH1/JH9-151	synonymous	a	g	2133544	SA1883	hypothetical protein
225/JH1/JH9-152	intergenic	g	c	2145642	intergenic	
225/JH1/JH9-153	synonymous	c	t	2146242	SA1893	lipoprotein precursor
225/JH1/JH9-154	intergenic	c	t	2182564	intergenic	
225/JH1/JH9-155	non-synonymous	g	a	2208217	SA1957	conserved hypothetical protein
225/JH1/JH9-156	non-synonymous	g	a	2223669	SA1964	FmtB protein
225/JH1/JH9-157	intergenic	t	c	2249040	intergenic	
225/JH1/JH9-158	non-synonymous	g	a	2253816	SA1983	hypothetical protein
225/JH1/JH9-159	synonymous	t	c	2262362	SA1990	conserved hypothetical protein
225/JH1/JH9-160	non-synonymous	t	c	2292592	SA2020	SA2020~hypothetical protein, similar to ABC transporter (ATP-binding
225/JH1/JH9-161	intergenic	g	t	2308775	intergenic	
225/JH1/JH9-162	synonymous	a	g	2339649	SA2079	SA2079~hypothetical protein, similar to ferrichrome ABC transporter
225/JH1/JH9-163	non-synonymous	a	c	2345241	SA2084	urease alpha subunit
225/JH1/JH9-164	intergenic	t	c	2348642	intergenic	
225/JH1/JH9-165	synonymous	g	a	2448777	SA2180	SA2180~hypothetical protein, similar to two component sensor
225/JH1/JH9-166	non-synonymous	g	a	2499035	SA2226/SA2227	truncated hypothetical protein, similar to ABC transporter (permease)
225/JH1/JH9-167	intergenic	a	g	2546437	intergenic	
225/JH1/JH9-168	non-synonymous	g	a	2555965	SA2279	SA2279~hypothetical protein, similar to phosphomannomutase
225/JH1/JH9-169	non-synonymous	g	t	2576747	SA2293	gluconate permease
225/JH1/JH9-170	non-synonymous	t	c	2628043	SA2339	SA2339~hypothetical protein, similar to antibiotic transport-associated protein
225/JH1/JH9-171	intergenic	g	a	2634823	intergenic	
225/JH1/JH9-172	intergenic	c	t	2634964	intergenic	
225/JH1/JH9-173	non-synonymous	a	c	2655416	SA2365	SA2365~hypothetical protein, similar to short chain oxidoreductase
225/JH1/JH9-174	synonymous	g	a	2681783	SA2394	SA2394~hypothetical protein, similar to alpha-acetolactate decarboxylase
225/JH1/JH9-175	non-synonymous	t	c	2710694	SA2415	SA2415~hypothetical protein, similar to ABC transporter (permease)

225/JH1/JH9-176	synonymous	g	a	2715136	SA2420	alkaline phosphatase III precursor
225/JH1/JH9-177	synonymous	c	a	2733152	SA2433	SA2433~hypothetical protein, similar to transcription antiterminator BglG
225/JH1/JH9-178	intergenic	g	t	2769109	intergenic	
225/JH1/JH9-179	intergenic	g	t	2769511	intergenic	
225/JH1/JH9-180	non-synonymous	t	g	2772534	SA2461	intercellular adhesion protein B
225/JH1/JH9-181	non-synonymous	c	t	2785873	SA2474	conserved hypothetical protein
**Indels.**						
225/JH1/JH9-182	deletion (997 nt)	-	-	42512	intergenic	*dru* deletion
225/JH1/JH9-183	insertion (36 nt)	-	-	615798	SA0521	Ser-Asp rich fibrinogen-binding, bone sialoprotein-binding protein
225/JH1/JH9-184	deletion (59 nt)	-	-	823709	intergenic	
225/JH1/JH9-185	deletion (252 nt)	-	-	850625	SA0742	fibrinogen-binding protein A, clumping factor
225/JH1/JH9-186	insertion (44088 nt)	-	-	885609	intergenic	insertion of prophage ΦSaST5K
225/JH1/JH9-187	insertion (2 nt)	-	-	1236474	SA1092	SA1092~hypothetical protein, similar to DNA processing Smf
225/JH1/JH9-188	insertion (100 nt)	-	-	2145667	intergenic	
225/JH1/JH9-189	insertion (45 nt)	-	-	2477341	SA2206	IgG-binding protein SBI
225/JH1/JH9-190	deletion (127 nt)	-	-	2634978	SAS092	hypothetical protein

Table S8d. Derived traits in N315.

Polymorphism	Quality	Ancestral	Derived	Position in N315 genome	ORF	Product
**Substitutions.**						
N315-1	intergenic	G	A	22264	intergenic	
N315-2	synonymous	T	G	46963	SA0038	penicillin binding protein 2 prime
N315-3	intergenic	C	G	95177	intergenic	
N315-4	intergenic	G	T	113292	intergenic	
N315-5	non-synonymous	T	G	129370	SA0112	SA0112~hypothetical protein, similar to cysteine synthase
N315-6	non-synonymous	T	G	129371	SA0112	SA0112~hypothetical protein, similar to cysteine synthase
N315-7	non-synonymous	C	T	168333	SA0145	capsular polysaccharide synthesis enzyme Cap5B
N315-8	intergenic	G	T	187180	intergenic	
N315-9	synonymous	A	G	203046	SA0173	SA0173~hypothetical protein, similar to surfactin synthetase
N315-10	intergenic	G	A	229156	intergenic	
N315-11	non-synonymous	C	T	263130	SA0220	SA0220~hypothetical protein, similar to glycerophosphodiester phosphodiesterase
N315-12	non-synonymous	T	A	271705	SA0225	SA0225~hypothetical protein, similar to glutaryl-CoA dehydrogenase
N315-13	non-synonymous	C	A	271986	SA0225	SA0225~hypothetical protein, similar to glutaryl-CoA dehydrogenase
N315-14	intergenic	T	C	281256	intergenic	
N315-15	non-synonymous	C	T	290472	SA0239	sorbitol dehydrogenase
N315-16	synonymous	A	G	352733	SA0297	SA0297~hypothetical protein, similar to ABC transporter ATP-binding
N315-17	non-synonymous	G	T	355647	SA0299	SA0299~hypothetical protein, similar to carbohydrate kinase, PfkB
N315-18	non-synonymous	C	T	384600	SA0326	conserved hypothetical protein
N315-19	intergenic	A	G	412719	intergenic	
N315-20	non-synonymous	G	A	419598	SA0362	hypothetical protein
N315-21	non-synonymous	G	T	432398	SA0374	xanthine permease
N315-22	non-synonymous	C	T	494193	SA0430	glutamate synthase large subunit
N315-23	synonymous	A	G	495702	SA0431	NADH-glutamate synthase small subunit
N315-24	non-synonymous	G	C	498615	SA0432	PTS enzyme II, phosphoenolpyruvate-dependent, trehalose-specific
N315-25	intergenic	C	T	505349	intergenic	

N315-26	non-synonymous	A	T	515553	SA0442	probable DNA polymerase III, delta prime subunit
N315-27	synonymous	G	A	521614	SA0449	conserved hypothetical protein
N315-28	intergenic	A	G	547168	intergenic	
N315-29	non-synonymous	G	A	577020	SA0496	50S ribosomal protein L1
N315-30	synonymous	C	T	578043	SA0497	50S ribosomal protein L10
N315-31	intergenic	G	A	579439	intergenic	
N315-32	synonymous	C	T	617499	SA0522	SA0522~hypothetical protein, similar to poly (glycerol-phosphate) alpha-glucosyltransferase
N315-33	non-synonymous	T	G	656531	SA0562	alcohol dehydrogenase I
N315-34	non-synonymous	C	T	663575	SA0569	hypothetical protein similar to 2-hydroxy-6-oxo-6-phenylhexa-2,4-dienoic acid hydrolase
N315-35	non-synonymous	C	T	674395	SA0581	MnhD homologue, similar to Na+/H+ antiporter subunit~SA0581
N315-36	non-synonymous	C	T	687696	SA0594	teichoic acid translocation permease protein
N315-37	intergenic	A	G	722570	intergenic	
N315-38	non-synonymous	G	A	732508	SA0639	SA0639~hypothetical protein, similar to ABC transporter required
N315-39	non-synonymous	T	A	736539	SA0642	SA0642~hypothetical protein, similar to cobalamin synthesis related
N315-40	intergenic	T	C	751602	intergenic	
N315-41	non-synonymous	T	C	754961	SA0659	SA0659~hypothetical protein, similar to CsbB stress response
N315-42	synonymous	T	A	756294	SA0660	histidine protein kinase
N315-43	intergenic	G	A	757254	intergenic	
N315-44	non-synonymous	A	T	762615	SA0669	SA0669~hypothetical protein, similar to para-aminobenzoate synthase component
N315-45	non-synonymous	C	T	817503	SA0714	exinuclease ABC subunit A
N315-46	non-synonymous	G	A	836657	SA0730	2, 3-diphosphoglycerate-independentphosphoglycer ate mutase
N315-47	non-synonymous	G	A	852025	SA0743	SA0743~hypothetical protein, similar to staphylocoagulase precursor
N315-48	intergenic	A	C	855633	intergenic	
N315-49	intergenic	C	T	856541	intergenic	
N315-50	non-synonymous	G	A	875851	SA0769	ABC transporter ATP-binding protein homologue~SA0769
N315-51	intergenic	C	A	885644	intergenic	
N315-52	non-synonymous	A	T	902157	SA0796	poly D-alanine transfer protein
N315-53	intergenic	C	A	941416	intergenic	
N315-54	non-synonymous	A	G	1064930	SA0938	SA0938~cytochrome D ubiquinol oxidase subunit II homolog
N315-55	non-synonymous	G	C	1084465	SA0957	conserved hypothetical protein

N315-56	synonymous	C	T	1089845	SA0962	conserved hypothetical protein
N315-57	synonymous	A	G	1122849	SA0991	MutS-like protein
N315-58	intergenic	G	T	1127179	intergenic	
N315-59	non-synonymous	A	C	1148754	SA1014	conserved hypothetical protein
N315-60	intergenic	C	T	1195974	intergenic	
N315-61	non-synonymous	G	C	1242927	SA1097	heat shock protein HslU
N315-62	synonymous	T	A	1265987	SA1116	30Sribosomal protein S15
N315-63	synonymous	C	T	1272672	SA1119	SA1119~sporulation-related protein SpoIIIE homolog
N315-64	non-synonymous	A	T	1273131	SA1120	SA1120~hypothetical protein, similar to transcription regulator GntR
N315-65	non-synonymous	C	A	1316374	SA1155	SA1155~cardiolipin synthetase homolog
N315-66	synonymous	T	C	1405911	SA1231	SA1231~hypothetical protein, similar to alanine racemase
N315-67	non-synonymous	G	T	1411253	SA1238	SA1238~hypothetical protein, similar to tellurite resistance protein
N315-68	non-synonymous	G	T	1419383	SA1245	2-oxoglutarate dehydrogenase E1
N315-69	non-synonymous	C	A	1428413	SA1253	probable carboxy-terminal processing proteinase ctpA
N315-70	non-synonymous	C	T	1431531	SA1258	conserved hypothetical protein
N315-71	intergenic	A	G	1433941	intergenic	
N315-72	non-synonymous	C	T	1436004	SA1264	conserved hypothetical protein
N315-73	non-synonymous	G	A	1444580	SA1267	SA1267~hypothetical protein, similar to streptococcal adhesin emb
N315-74	non-synonymous	G	A	1458132	SA1268	SA1268~hypothetical protein, similar to streptococcal adhesin emb
N315-75	non-synonymous	A	G	1487245	SA1283	PBP2
N315-76	synonymous	T	C	1501671	SA1295	conserved hypothetical protein
N315-77	non-synonymous	T	A	1519779	SA1312	elastin binding protein
N315-78	non-synonymous	G	A	1525060	SA1317	hypothetical protein
N315-79	non-synonymous	G	T	1546565	SA1338	alpha-D-1,4-glucosidase
N315-80	non-synonymous	T	C	1592159	SA1387	SA1387~hypothetical protein, similar to ATP-dependent RNA helicase
N315-81	non-synonymous	T	C	1595731	SA1390	RNA polymerase sigma factor
N315-82	synonymous	T	A	1601071	SA1394	glycyl-tRNA synthetase
N315-83	non-synonymous	C	T	1608155	SA1403	conserved hypothetical protein
N315-84	non-synonymous	G	A	1609428	SA1405	conserved hypothetical protein
N315-85	synonymous	T	C	1619150	SA1413	GTP-binding protein

N315-86	non-synonymous	G	T	1622147	SA1415	conserved hypothetical protein
N315-87	synonymous	C	T	1665174	SA1458/SA1459	N-acetylmuramoyl-L-alanine amidase/conserved hypothetical protein
N315-88	intergenic	T	A	1710667	intergenic	
N315-89	non-synonymous	T	C	1715782	SA1506	threonyl-tRNA synthetase 1
N315-90	intergenic	G	A	1720424	intergenic	
N315-91	intergenic	T	A	1720487	intergenic	
N315-92	non-synonymous	A	G	1750617	SA1531	alanine dehydrogenase
N315-93	non-synonymous	G	C	1756434	SA1537	SA1537~hypothetical protein, similar to thiamine biosynthesis protein
N315-94	non-synonymous	G	A	1758442	SA1539	SA1539~hypothetical protein, similar to septation ring formation
N315-95	non-synonymous	C	G	1763490	SA1542	SA1542~hypothetical protein, similar to glycerophosphoryl diester phosphodiesterase
N315-96	non-synonymous	A	T	1776646	SA1552	hypothetical protein
N315-97	non-synonymous	T	A	1783401	SA1556	acetoin utilization protein
N315-98	non-synonymous	T	C	1800117	SA1569	conserved hypothetical protein
N315-99	intergenic	C	T	1830770	intergenic	
N315-100	non-synonymous	A	G	1837019	SA1599	SA1599~hypothetical protein, similar to transaldolase
N315-101	intergenic	C	T	1871939	intergenic	
N315-102	intergenic	A	G	1873989	intergenic	
N315-103	non-synonymous	G	A	1914044	SA1676	SA1676~hypothetical protein, similar to regulatory protein (pfoS/R)
N315-104	non-synonymous	A	G	1964481	SA1716	glutamyl-tRNAGln amidotransferase subunit A
N315-105	intergenic	A	T	2080266	intergenic	
N315-106	non-synonymous	T	G	2094604	SA1854	SA1854~hypothetical protein, similar to O-sialoglycoprotein endopeptidase
N315-107	synonymous	C	T	2099406	SA1859	acetolactate synthase large subunit
N315-108	non-synonymous	A	T	2107968	SA1866	thereonine dehydratase
N315-109	intergenic	T	C	2145572	intergenic	
N315-110	intergenic	T	C	2145582	intergenic	
N315-111	intergenic	A	T	2145611	intergenic	
N315-112	intergenic	G	A	2145636	intergenic	
N315-113	intergenic	T	C	2145669	intergenic	
N315-114	non-synonymous	G	T	2171885	SA1923	transcription termination factor Rho
N315-115	synonymous	G	A	2228111	SA1966	conserved hypothetical protein

N315-116	non-synonymous	C	T	2264705	SA1992	PTS system, lactose-specific IIBC component
N315-117	intergenic	T	C	2269182	intergenic	
N315-118	synonymous	C	T	2293874	SA2021	SA2021~hypothetical protein, similar to ABC transporter (ATP-binding
N315-119	non-synonymous	A	T	2303560	SA2038	30S ribosomal protein S17
N315-120	non-synonymous	C	G	2402254	SA2136	isopentenyl diphosphate isomerase
N315-121	intergenic	C	T	2405712	intergenic	
N315-122	non-synonymous	G	A	2412317	SA2146	TcaA protein
N315-123	synonymous	A	G	2422321	SA2156	L-lactate permease lctP homolog~SA2156
N315-124	synonymous	G	A	2451980	SA2184	nitrate reductase beta chain narH
N315-125	non-synonymous	A	G	2506840	SA2232	SA2232~hypothetical protein, similar to 2-dehydropantoate 2-reductase
N315-126	non-synonymous	C	T	2513726	SA2238	conserved hypothetical protein
N315-127	synonymous	T	C	2550966	SA2275	hypothetical protein
N315-128	non-synonymous	G	T	2553138	SA2277	conserved hypothetical protein
N315-129	non-synonymous	G	A	2573237	SA2291	SA2291~fibronectin-binding protein homolog
N315-130	non-synonymous	A	G	2586185	SA2301	SA2301~hypothetical protein, similar to alkaline phosphatase
N315-131	non-synonymous	C	G	2609706	SA2326	PTS system, glucose-specific IIABC component
N315-132	non-synonymous	C	T	2670294	SA2381	hypothetical protein
N315-133	intergenic	A	T	2700440	intergenic	
N315-134	intergenic	G	C	2708904	intergenic	
N315-135	intergenic	C	A	2729661	intergenic	
N315-136	non-synonymous	A	C	2731038	SA2432	conserved hypothetical protein
N315-137	non-synonymous	C	T	2753061	SA2445	hypothetical protein
N315-138	synonymous	G	A	2755100	SA2446	SA2446~hypothetical protein, similar to preprotein translocase secY
N315-139	synonymous	G	A	2755727	SA2447	SA2447~hypothetical protein, similar to streptococcal hemagglutinin protein
N315-140	intergenic	T	C	2785787	intergenic	
N315-141	synonymous	G	T	2787033	SA2475	conserved hypothetical protein

Indels.

N315-142	deletion (174 nt)	-	-	123300	SA0107	Immunoglobulin G binding protein A precursor
N315-143	insertion (1520 nt)	-	-	426552	intergenic	

N315-144	insertion (168 nt)	-	-	830908	intergenic	
N315-145	insertion (6712 nt)	-	-	866874	SA0761	conserved hypothetical protein, truncated by insertion of Tn554-b1
N315-146	deletion (190 nt)	-	-	924619	intergenic	
N315-147	deletion (66 nt)	-	-	1421764	SA1245	2-oxoglutarate dehydrogenase E1
N315-148	deletion (16 nt)	-	-	1423801	SA1248	truncated (putative response regulator ArlR [S
N315-149	deletion (116 nt)	-	-	1437166	intergenic	
N315-150	deletion (1 nt)	-	-	1437291	intergenic	
N315-151	insertion (1520 nt)	-	-	1761585	intergenic	insertion of IS 1181
N315-152	insertion (15659 nt)	-	-	2056682	intergenic	
N315-153	deletion (12)	-	-	2076132	SA1839	SA1839~hypothetical protein, similar to SdrH
N315-154	insertion (6712 nt)	-	-	2198760	SA1949	lytic regulatory protein; truncated by insertion of Tn554-b2
N315-155	deletion (63 nt)	-	-	2244296	intergenic	
N315-156	deletion (191 nt)	-	-	2475668	intergenic	
N315-157	insertion (14 nt)	-	-	2499013	SA2226	SA2226~truncated hypothetical protein, similar to ABC transporter
N315-158	insertion (6712 nt)	-	-	2671104	intergenic	insertion of Tn554-c
